# Revised Lithostratigraphy of the Sonsela Member (Chinle Formation, Upper Triassic) in the Southern Part of Petrified Forest National Park, Arizona

**DOI:** 10.1371/journal.pone.0009329

**Published:** 2010-02-19

**Authors:** Jeffrey W. Martz, William G. Parker

**Affiliations:** Division of Resource Management, Petrified Forest National Park, Petrified Forest, Arizona, United States of America; Raymond M. Alf Museum of Paleontology, United States of America

## Abstract

**Background:**

Recent revisions to the Sonsela Member of the Chinle Formation in Petrified Forest National Park have presented a three-part lithostratigraphic model based on unconventional correlations of sandstone beds. As a vertebrate faunal transition is recorded within this stratigraphic interval, these correlations, and the purported existence of a depositional hiatus (the Tr-4 unconformity) at about the same level, must be carefully re-examined.

**Methodology/Principal Findings:**

Our investigations demonstrate the neglected necessity of walking out contacts and mapping when constructing lithostratigraphic models, and providing UTM coordinates and labeled photographs for all measured sections. We correct correlation errors within the Sonsela Member, demonstrate that there are multiple Flattops One sandstones, all of which are higher than the traditional Sonsela sandstone bed, that the Sonsela sandstone bed and Rainbow Forest Bed are equivalent, that the Rainbow Forest Bed is higher than the sandstones at the base of Blue Mesa and Agate Mesa, that strata formerly assigned to the Jim Camp Wash beds occur at two stratigraphic levels, and that there are multiple persistent silcrete horizons within the Sonsela Member.

**Conclusions/Significance:**

We present a revised five-part model for the Sonsela Member. The units from lowest to highest are: the Camp Butte beds, Lot's Wife beds, Jasper Forest bed (the Sonsela sandstone)/Rainbow Forest Bed, Jim Camp Wash beds, and Martha's Butte beds (including the Flattops One sandstones). Although there are numerous degradational/aggradational cycles within the Chinle Formation, a single unconformable horizon within or at the base of the Sonsela Member that can be traced across the entire western United States (the “Tr-4 unconformity”) probably does not exist. The shift from relatively humid and poorly-drained to arid and well-drained climatic conditions began during deposition of the Sonsela Member (low in the Jim Camp Wash beds), well after the Carnian-Norian transition.

## Introduction

Geologists and paleontologists are ultimately historians whose objective is to construct an accurate narrative of the history of the Earth and its living organisms, and to understand why these events occurred. Biostratigraphy, magnetostratigraphy, radioisotopic dating, the interpretation of depositional systems and paleoclimatology, are all tools for deriving a historical narrative from the rock record. However, if the basic superpositional relationships of the fossils, mag-strat samples, volcanic minerals, and lithologic units used to acquire this information are misunderstood, the interpretation derived from them will be inaccurate. The order and timing of events will be wrong, and any attempt to understand cause and effect will be in vain. Lithostratigraphy is therefore the foundation of paleontology as a historical science. Developing an accurate and detailed lithostratigraphic framework is the first and most essential step before anything collected from these strata can be used to construct a narrative.

**Figure 1:**
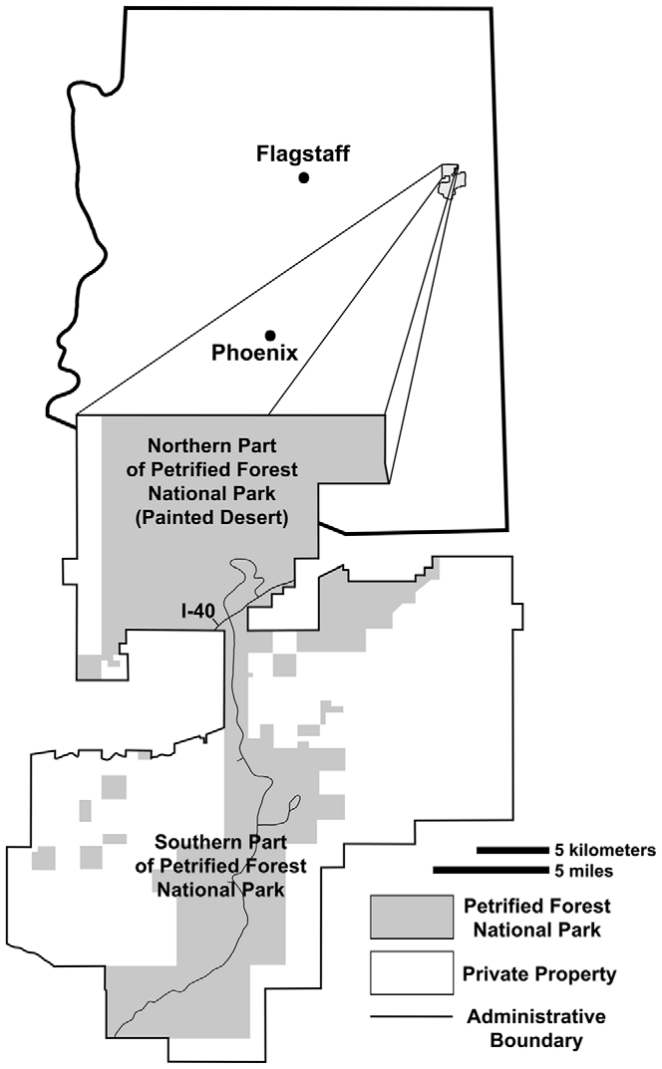
Map of PEFO and its location in northeastern Arizona.

**Figure 2:**
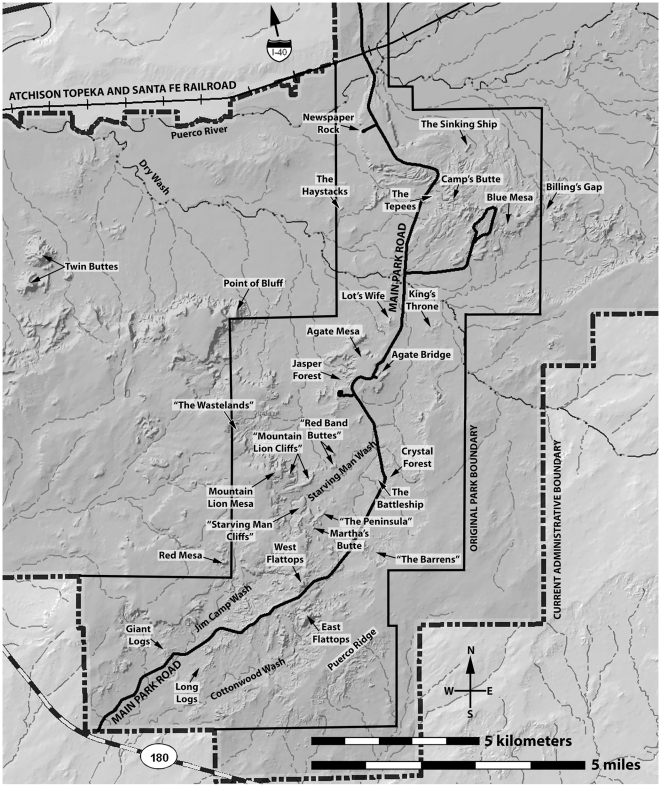
Important geographic features in the southern part of PEFO. Features named in for the first time in this paper in quotation marks.

**Figure 3:**
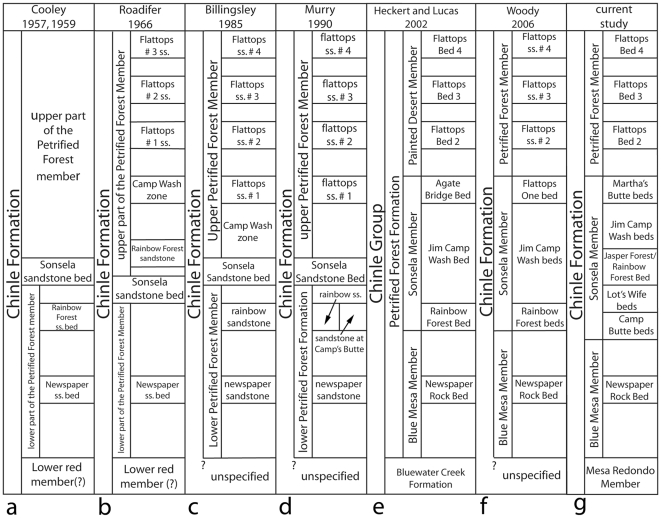
Lithostratigraphic nomenclature for the southern part of Petrified Forest National Park. Stratigraphic models shown for Cooley [16] (a); Roadifer [17] (b); Billingsley [18] (c); Murry [20] (d); Heckert and Lucas [11] (e); Woody [12] (f); and the current study (g). Due to the fact that these models differ in terms of lithostratigraphic correlation as well as nomenclature, not all units shown in adjacent columns can be shown as truly equivalent. For example, the Agate Bridge Bed of Heckert and Lucas [11], which is also the Flattops One Bed of Woody [12], is actually equivalent to both the Jasper Forest bed/Rainbow Forest Bed and Martha's Butte beds of the current study. Figure 4 better illustrates these correlation differences.

The Chinle Formation of the Colorado Plateau, and related strata throughout the western United States, preserve some of the most extensively exposed and well-studied Late Triassic continental deposits in the world [1]–[3]. These strata also preserve one of the best-studied terrestrial vertebrate faunas from this critical period in the Earth's history (e.g., [4]). The Upper Triassic strata and vertebrate fossils in Petrified Forest National Park (hereafter PEFO) in northeastern Arizona (Figures 1–2) are arguably the most intensively studied in the Western Interior for several reasons:

1. PEFO and the surrounding area has had a long history of research, with significant investigations into the sedimentary geology and paleontology of the Chinle Formation dating back to the first half of the 20^th^ century (e.g., [5]–[6]). The Chinle Formation remains a rich source of plant and animal fossils, the collection and description of which is ongoing by researchers from various institutions, including the park staff (e.g., [7]–[9]).
2. Almost the full section of the Chinle Formation is exposed within PEFO. Most of the park has excellent exposures of the middle part of the Chinle Formation, which has traditionally been referred to as the Petrified Forest Member, and has more recently been formally divided into the Blue Mesa, Sonsela, and Petrified Forest (or Painted Desert) Members [3], [10]–[12] (Figure 3). Lowermost Chinle Formation strata (variously referred to as the Monitor Butte, Bluewater Creek, or Mesa Redondo Members or Formations; [3], [7], [11], [13], [14]) are well-exposed just south of the Puerco River in the recent PEFO boundary expansion. Upper Chinle Formation strata (the Owl Rock Member) are exposed at Chinde Mesa and Pilot Rock in the Painted Desert region of the park [15], although the uppermost Chinle Formation (the Rock Point Member) is not preserved within the park boundaries.
3. As a national park, PEFO is fully accessible to researchers. A strong effort has been made in recent years by one of us (WGP) not only to facilitate geological and paleontological research within the park, but to orchestrate efforts by numerous researchers at various institutions in order to help construct a comprehensive synthesized model of Chinle Formation lithostratigraphy, depositional systems, magnetostratigraphy, chronostratigraphy, and biostratigraphy.

From the 1950s through the end of the 20^th^ century, the basic lithostratigraphic framework of the Chinle Formation within the southern part of PEFO was thought to be well understood, with only minor disagreements (e.g., [5], [16]–[22]), and a significant turnover of the vertebrate fauna was recognized as occurring within the beds most workers called the Petrified Forest Member [3], [5], [23]–[25]. However, recent revisions to the lithostratigraphy of the traditional Petrified Forest Member within PEFO [11]–[12] have made this turnover appear to be more gradual than previously thought, with a period of overlap between the faunas [7], [26]–[28]. Geologic mapping has revealed problems with the new correlations on which this revised lithostratigraphic model is based (e.g.,[29]), indicating that older lithostratigraphic models may have been more accurate. Careful lithostratigraphic revisions are therefore required to clarify the nature of the faunal turnover. Additionally, existence of the Tr-4 unconformity [3], [30], [31], an alleged erosional hiatus marking the faunal turnover, has been called into question [12], [32].

After almost a century of research, it is astonishing that controversy remains about the basic lithostratigraphic framework of the Chinle Formation in Petrified Forest National Park, and it is absolutely essential to resolve these controversies before the nature and timing of faunal and floral change during the Late Triassic in northeastern Arizona can be understood. The lithostratigraphy of the Chinle Formation, particularly those strata recently assigned to the Sonsela Member by Heckert and Lucas [11] and Woody [12], has been carefully re-examined. The goal of this study is to precisely assess the correlation of lithologic units within this interval, and therefore the basic lithostratigraphic framework, which in critical for understanding both depositional and biotic change during the Late Triassic of western North America. This paper deals with lithostratigraphic revisions within the southern part of the park (Figure 2), and forthcoming papers will revise the lithostratigraphy of the northern part of the park and consider the implications of these revisions for biostratigraphy.

### Previous Studies of the Lithostratigraphy and Stratigraphic Nomenclature of the Traditional Petrified Forest Member in the Southern Part of Petrified Forest National Park

The reader is referred to Stewart et al. [1], [33] for detailed reviews of early studies of the Upper Triassic rocks of the Colorado Plateau, including in northeast Arizona, in the late 19^th^ and early 20^th^ century. However, the modern nomenclature applied to the Chinle Formation began with Gregory [34], who named the unit for exposures in the Navajo Indian Reservation north of present-day Petrified Forest National Park. Gregory also recognized a separate lower unit, the Shinarump conglomerate (originally named by J.W. Powell), which is now considered to be a basal member of the Chinle Formation [35]. Gregory subdivided the Chinle Formation above the Shinarump conglomerate into four “divisions”, numbered, from highest to lowest, A, B, C, and D. Upper Divisions A and B correspond respectively to what are now called the Rock Point Member [36], which is not present within the park boundaries, and the Owl Rock Member [37]. The lowermost Division D corresponds to strata in the park variously correlated, with much disagreement, to the Monitor Butte Member, Mesa Redondo Member, lower red member, and/or Bluewater Creek Members of the Chinle Formation, or to the older Moenkopi Formation [3], [11], [13], [14], [21], [38]–[40].

Gregory's [34] “Division C” of the Chinle Formation, consisting of variegated mudstone with interbedded lenses of sandstone and conglomerate, is the most widely exposed unit of the Chinle Formation within Petrified Forest National Park. These strata were later named the Petrified Forest Member of the Chinle Formation by Gregory [41], although his type section is actually located within Zion National Park in southwestern Utah, and probably only correlative to the upper part of the unit traditionally assigned this name in PEFO [12], [42]. Correlative strata throughout northern Arizona, southern Utah, northwestern and north-central New Mexico, and southern Nevada, have also been assigned to the Petrified Forest Member (e.g., [1]–[3], [10], [43]).

In northern Arizona, the Petrified Forest Member of Gregory [41] was divided into three parts by Akers et al. [13] and Repenning et al. [10]. The lower Petrified Forest Member and upper Petrified Forest Member are both mudstone-dominated units with interbedded sandstone and conglomerate, but are distinct from each other in terms of coloration and lithology. Dividing the lower and upper parts of the Petrified Forest Member is a package of siliceous conglomeratic sandstones and interbedded mudstone called the Sonsela sandstone bed (or sometimes simply the Sonsela sandstone) (Figure 3). The type area of the Sonsela sandstone bed is along the east flank of the Defiance Uplift north of Petrified Forest, near the Arizona-New Mexico state line, where the unit is 120–200 feet thick and consists of two conglomeratic sandstone beds separated by siltstone [13].

**Figure 4:**
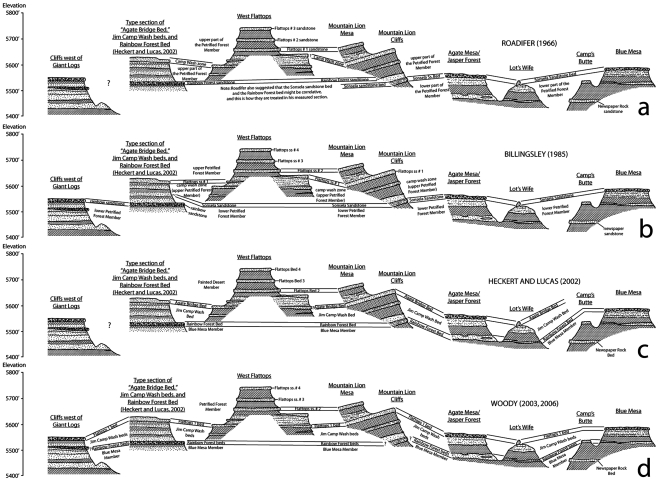
Prior correlations and nomenclature for the Chinle Formation in the southern part of PEFO. Correlations shown between Blue Mesa, Agate Mesa and Lot's Wife, Mountain Lion Cliffs and Mountain Lion Mesa, the Flattops, the cliffs north of Giant Logs, and the cliffs near the south entrance station. Correlations shown for Roadifer [17] (a); Billingsley [18] (b); Heckert and Lucas [11] (c); and Woody [12] (d).

Within PEFO itself, the name “Sonsela sandstone bed” has long been assigned to a siliceous conglomeratic sandstone that caps Agate Mesa, Blue Mesa, and the bluffs north of Crystal Forest (Figure 2, Figure 4) in the southern part of the park, north of the mesas known as the Flattops [16], [17], [19], [20]. Southwest of the Flattops, Cooley [16] identified a second siliceous conglomeratic sandstone unit, the Rainbow Forest sandstone bed (sometimes simply called the Rainbow Forest sandstone or Rainbow sandstone) (Figure 3a). Cooley recognized that the Rainbow Forest sandstone bed and Sonsela sandstone bed are similar in their lithology and bedding structures, that both contain large gravel-sized clasts of silicified Paleozoic limestone, and that both produce abundant colorful petrified wood.

It is curious therefore that Cooley [16] identified the Sonsela sandstone bed and Rainbow Forest sandstone bed as being stratigraphically distinct within Petrified Forest National Park, with the Rainbow Forest sandstone bed occurring slightly lower in the section, near the top of the lower Petrified Forest Member. This convention has been followed by most subsequent workers [11], [12], [18]–[20], [29] (Figure 3, Figure 4) even though neither unit can be traced continuously across the Flattops. Most of these authors claimed to be able to identify the Sonsela sandstone bed southwest of the Flattops above the Rainbow Forest sandstone bed, usually as a thinner and finer-grained unit than the thicker and more conglomeratic bed capping Blue Mesa and Agate Mesa north of the Flattops. Important exceptions are Roadifer [17], who claimed that the Rainbow Forest sandstone bed could be identified north of the Flattops about 20 feet *above* the Sonsela sandstone bed (Figure 3b, Figure 4a), and several authors [44]–[46], who suggested that the Sonsela sandstone bed and Rainbow Forest sandstone bed were correlative. This latter possibility was also suggested by Roadifer [17] and Murry [20], although they seem to have favored the interpretation that they are separate units.

Several prominent sandstone layers are present in both the lower and upper parts of the Petrified Forest Member within PEFO. These sandstones are generally finer-grained and less conglomeratic than the Sonsela sandstone bed and Rainbow Forest sandstone bed. The Newspaper Rock sandstone ([6]; the “Pictograph Sandstone” of Camp [5]) lies within the lower Petrified Forest Member in the southern part of PEFO, stratigraphically below the level of the Rainbow Forest sandstone bed [6], [16], [17], [47], [48] (Figure 3).

**Figure 5:**
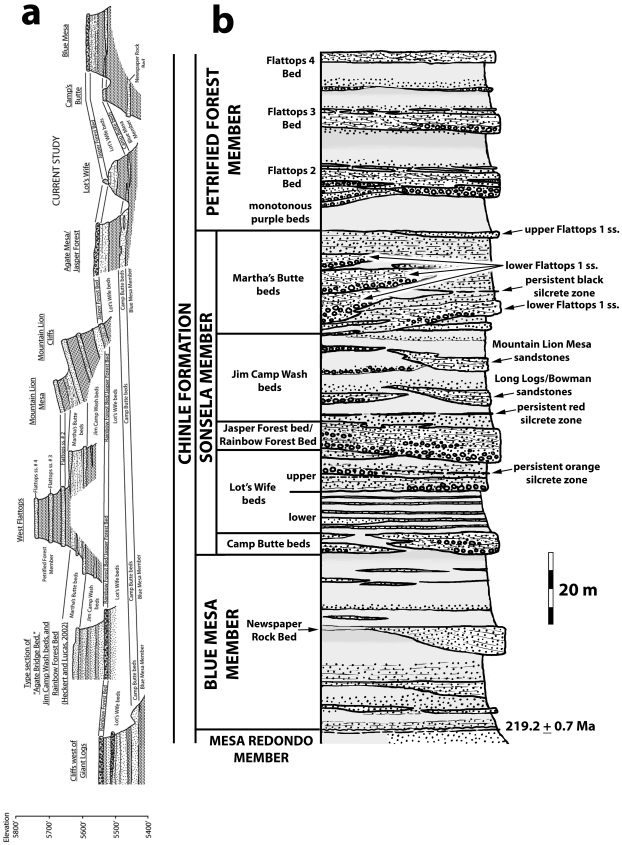
Revised lithostratigraphic model for the Chinle Formation in the southern part of PEFO. Revised correlations between Blue Mesa, Agate Mesa and Lot's Wife, Mountain Lion Cliffs and Mountain Lion Mesa, the Flattops, the cliffs north of Giant Logs, and the cliffs near the south entrance station (a); composite lithostratigraphic model (b).

Roadifer [17] provided the first detailed discussion of the sandstones in the upper Petrified Forest Member in the Flattops region, in addition to attempting to correlate these sandstones to those exposed in the upper Petrified Forest Member in the Painted Desert. Roadifer ([17]p.20–21) described the “Camp Wash zone” as “a series of sandstone lenses that generally contain basal limestone-pebble conglomerates and that are separated by layers of mudstone and siltstone” with a thickness “generally between five and twenty feet.” These thicknesses, and the fact that he identified the unit as occurring “approximately 90 feet above the Sonsela sandstone bed,” suggest he was restricting the term to the package of resistant, cliff-forming sandstones later re-named Flattops sandstone number 1 by Billingsley [18] (Figures 3b–c, Figures 4a–b), and not to the generally more friable and slope-forming sandstones and mudstones exposed below, directly above the Sonsela sandstone bed. This is confirmed by examining his stratigraphic sections ([17]figs. 3, 5, 25). Roadifer [17] also provided names for the prominent ledge-forming sandstones lying stratigraphically above the “Camp Wash zone” at the Flattops and the surrounding areas, designating them (from lowest to highest) the Flattops number 1 sandstone, Flattops number 2 sandstone, and Flattops number 3 sandstone, with the last capping the highest tier of mesas at the Flattops.

Billingsley [18] provided some major revisions to Roadifer's [17] nomenclature (Figures 3b–c, Figures 4a–b), particularly in re-numbering the Flattops sandstones. Billingsley [18] re-named the “Camp Wash zone” as “Flattops sandstone number 1”, and consequently also re-numbered Roadifer's [17] Flattops 1–3 sandstones as Flattops sandstones numbers 2–4. Billingsley ([18]p. 6, fig. 2) also applied Roadifer's [17] term “Camp Wash zone” to the slope-forming strata lying between his re-numbered Flattops number 1 sandstone and the Sonsela sandstone bed. Billingsley's ([18]p.6) explanation, that Roadifer [17] had allegedly included the upper part of the Sonsela sandstone bed in the Camp Wash zone was probably based on Roadifer's ([17]p.16) tentative suggestion that the Camp Wash zone might be an upper tongue of the Sonsela sandstone bed. However, although this would make the intervening strata also part of the Sonsela sandstone bed, it would not make these same strata part of the “Camp Wash zone”, as Roadifer's usage of the term in his sections and correlations makes clear. Billingsley's revised lithostratigraphic nomenclature has been followed by most subsequent workers (Figures 3d–f). Billingsley [49] also mapped PEFO, clearly showing how he correlated units throughout the park.

Beginning in the early 1990s, several important changes to the lithostratigraphy and lithostratigraphic nomenclature of the Chinle Formation, including within Petrified Forest National Park, were made by Spencer Lucas and his colleagues. Lucas [3] elevated the Chinle Formation to group status, and more significantly, extended its usage to all Upper Triassic continental strata in the western United States. This had the consequence of elevating all members of the Chinle “Group” to formation status, including the Mesa Redondo Formation, Petrified Forest Formation, and Owl Rock Formation within the park. Lucas [3] also applied formal names to the lower, middle, and upper parts of the Petrified Forest Formation, naming them the Blue Mesa Member, Sonsela Member, and Painted Desert Member respectively. It is important to note that Lucas did not (at this time) modify the basic lithostratigraphic framework of the Petrified Forest Formation in the park established by previous workers, only the nomenclature. Many workers (e.g., [2], [7], [12] have rejected Lucas' elevation of the Chinle Formation to group status, and the more traditional ranking of the Chinle Formation will be used here.

In recent years, Heckert and Lucas [11], based mostly on Heckert's [50] master's thesis and Woody [12], based on his own [42] thesis, have correlated some of the prominent ledge-forming sandstones in the traditional Petrified Forest Member in the southern part of PEFO in a very different way than recognized by previous workers, with accompanying modifications to the nomenclature (Figures 3e–f, Figures 4c–d):

1. Heckert and Lucas [11] formalized the Rainbow Forest sandstone bed as the Rainbow Forest Bed, and they and Woody [12] correlated the unit across the Flattops with the sandstones exposed at the base of Agate Mesa (Figures 4c–d). Woody [12] likewise correlated the same sandstones at the base of Agate Mesa with those exposed at the base of Blue Mesa, including the prominent conglomeratic sandstone capping Camp's Butte [20]. Woody [12] referred to these sandstones as the “Rainbow Forest beds,” and noted that there were at least two discontinuous sandstones exposed in this interval at Lot's Wife, just north of Agate Mesa. He identified these two discontinuous sandstones of the Rainbow Forest beds as the “Rainbow Forest sandstone” and the “Camp Butte sandstone.” These correlations are fairly conservative in identifying the Rainbow Forest Bed as lying below the Sonsela sandstone bed, although this was the first time the former had actually been identified in this stratigraphic position *north* of the Flattops.
2. Whereas most previous workers had correlated the conglomeratic sandstones capping Agate Mesa and Blue Mesa, referring to both as the Sonsela sandstone bed, Heckert and Lucas ([11]fig. 4B, sections 18 and 23) correlated the sandstones exposed at the base of Agate Mesa that they identified as the Rainbow Forest Bed with the unit capping Blue Mesa (*contra* [50]figs. 2.5A–B), suggesting that the section exposed at Blue Mesa was stratigraphically below that exposed in the vicinity of Agate Bridge, Jasper Forest, and the bluffs to the west of Jasper Forest. Woody [12] rejected this particular correlation, noting that a distinctive silcrete horizon was present in the strata exposed below the Sonsela sandstone bed at both Agate Mesa and Blue Mesa which supported the more traditional correlation, which was later acknowledged by Lucas et al. [51].
3. Whereas most workers had considered Flattops sandstone number 1 (*sensu* [18]; the “Camp Wash zone” *sensu* [17]) to lie stratigraphically above the Sonsela sandstone bed, Heckert and Lucas ([11]fig. 4, sections 1–10 vs. sections 11–18) considered these sandstones correlative. They named this unit the “Agate Bridge Bed”, and identified the type section just northwest of Rainbow Forest, about seven miles southwest of Agate Bridge (which is at Agate Mesa). Woody [12] agreed with this revised correlation, but simply continued to refer to this unit as “Flattops One bed.” This correlation of the Sonsela sandstone bed with Flattops sandstone number one is the most unconventional presented by these workers, and the most significant for reasons discussed later.
4. Based on these revised correlations, these workers re-characterized the Sonsela Member as a package of two conglomeratic sandstones sandwiching a section of interbedded sandstone and mudstone (Figure 3e–f) that had previously been considered part of the Blue Mesa Member and/or upper Petrified Forest (Painted Desert) Member. The “Agate Bridge Bed” (Flattops sandstone number 1/former Sonsela sandstone bed) represents the upper conglomeratic sandstone, and Rainbow Forest Bed (including the Camp Butte sandstone) the lower. The section of interbedded sandstone and mudstone in between was named the “Jim Camp Wash Bed” by Heckert and Lucas [11], based on the misunderstanding (originating with Billingsley [18]) that the term “Camp Wash zone” of Roadifer [17] referred to the strata *below* Flattops sandstone number one. Heckert and Lucas [11] designated the same type locality for the Jim Camp Wash Bed as for the Agate Bridge Bed and Rainbow Forest Bed, northwest of Rainbow Forest. Woody [12] referred to this package by the more informal name “Jim Camp Wash beds.” These workers correlated this package across the Flattops to the section exposed at Agate Mesa, and Woody [12], [42] also correlated it to the section exposed at Blue Mesa.
5. Although (with the exception of the correlations between Blue Mesa and Agate Mesa), the revised lithostratigraphic models of Heckert and Lucas [11] and Woody [42] are the same, one important nomenclatural difference should be noted (Figures 3e–f). Heckert and Lucas [11] referred to the Blue Mesa Member, Sonsela Member, and Painted Desert Member as being part of the Petrified Forest Formation, which was in turn part of the Chinle Group. However, Woody [12] ceased the practice of uniting these units within a larger Petrified Forest Member or Formation altogether and simply made them independent members of the Chinle Formation. Moreover, Woody [12], noting that the type section of the Petrified Forest Member in Zion National Park is probably only correlative with the Painted Desert Member (upper Petrified Forest Member) in PEFO, suggested referring to the latter simply as the Petrified Forest Member of the Chinle Formation, a considerable restriction of the term within the park from its traditional usage. Woody's nomenclature will be used here.

Particularly in correlating the Sonsela sandstone bed and Flattops sandstone number 1, which had previously been considered to be superpositionally distinct, Heckert and Lucas [11] and Woody [12] considerably condensed the section in the middle of the traditional Petrified Forest Member. However, Raucci et al. [29] and Parker [7] raised questions regarding these revised correlations, claiming that Flattops sandstone number 1 exposed at the Flattops and just west of Mountain Lion Mesa is stratigraphically higher than the traditional Sonsela sandstone bed capping Agate Mesa, as previously alleged by most workers (e.g., [17], [20], [49], [52]) (Figures 4a–b).

### The Tr-4 Unconformity

Pipiringos and O'Sullivan [53] recognized several major and regionally widespread unconformities within Triassic and Jurassic strata of the western United States. Their Tr-1 and Tr-2 unconformities lie at the base of and within Lower-Middle Triassic strata such as the Moenkopi Formation, while their Tr-3 unconformity lies at the base of the Upper Triassic section, including at the base of the Chinle Formation. Following this numbering scheme, Lucas [3], [30] identified two additional major unconformities within the Upper Triassic strata of the western United States, which he called the Tr-4 and Tr-5 unconformities. Lucas interpreted the Tr-3, Tr-4, and Tr-5 unconformities as representing low-stand erosion across the entire Western Interior due to a eustatic drop in sea level. Within Petrified Forest National Park, the Tr-4 unconformity was identified by Lucas [3] as occurring at the base of the “Sonsela Member,” which at the time referred only to the Sonsela sandstone bed [22], [46].

Lucas [3], [30] and Heckert and Lucas [31] offered several lines of evidence that the Tr-4 unconformity represents a major erosional hiatus which extended across the Western Interior. These included: 1) evidence of downcutting into and reworking of strata immediately below the unconformities (including the top of the Blue Mesa Member), 2) the presence of a major lithological change in strata above the unconformity from that below it, and 3) evidence for an abrupt reorganization of the vertebrate fauna occurring across the unconformity (specifically between the “Adamanian” and “Revueltian” faunas of Lucas and Hunt [54]). This abrupt faunal change was interpreted to represent a considerable gap in time being represented by the Tr-4 unconformity.

In their stratigraphic and nomenclatural revisions of the Chinle Formation, Heckert and Lucas [11] stratigraphically relocated the Tr-4 unconformity at Petrified Forest National Park. Under Heckert and Lucas' [11] revised stratigraphy and nomenclature, the “Agate Bridge Bed”, which represents both the Sonsela sandstone bed and Flattops sandstone number one, lies near the top of their revised Sonsela Member. However, Heckert and Lucas ([11]p.13) continue to describe the Tr-4 unconformity as occurring at the base of the Sonsela Member, which in their revised nomenclature would place it well down section from the Agate Bridge Bed, at the base of the Rainbow Forest Bed. Given that the Tr-4 unconformity allegedly represents a major erosional event tied to eustatic sea-level change, and also marks a significant break in the vertebrate fossil record, relocating it stratigraphically is a move of real significance. Moreover, this relocation of the Tr-4 unconformity implies slightly revised lithostratigraphic and possibly chronostratigraphic correlations with other Upper Triassic strata of the western United States where the unconformity is allegedly present [3]. Unfortunately, Heckert and Lucas [11] do not provide an explanation from why they relocated the Tr-4 unconformity stratigraphically within PEFO. Furthermore, Hunt et al. [27] reinterpreted the Adamanian-Revueltian faunal transition as being more gradational than previously thought within the Sonsela Member, but did not discuss the implications of this towards the Tr-4 unconformity representing a major erosional hiatus.

Herrick [52], Woody [12], and Martz [32] questioned the existence of the Tr-4 unconformity, at least as a single regionally widespread erosional surface, based on their investigations in the Chinle Formation of PEFO and the Dockum Group of West Texas. Herrick [52] noted that there is no evidence of extensive paleosol formation below the Sonsela sandstone bed in PEFO, as would be expected from an extended depositional hiatus. Woody [12] determined that the base of the Sonsela Member (*sensu* [11]) consists of a series of discontinuous sheet sandstones that individually incise the underlying Blue Mesa Member, but do not rest on a single erosional unconformity. Woody ([12]p. 29) concluded therefore that “the Tr-4 unconformity must either be limited in distribution to areas north and west of PEFO, or is not a regionally significant surface.” Moreover, May ([55]fig. 2.15) and Martz [32] traced sandstones indentified as the Trujillo Sandstone of the Dockum Group (e.g., [56]), which also allegedly lies above the Tr-4 unconformity [3], along the eastern edge of the High Plains of West Texas. They demonstrated that these blanket sandstones are laterally extensive but ultimately discontinuous, so that the boundary between the mudstones of the underlying Tecovas Formation and those interbedded with these blanket sandstones is locally gradational, falsifying the existence of a single unconformable surface at the base of the Trujillo Sandstone.

### The Age of the Chinle Formation

The numeric ages of boundaries between the Carnian, Norian, and Rhaetian stages of the Upper Triassic have undergone recent re-appraisal. The Carnian-Norian and Norian-Rhaetian boundaries, which were previously thought to occur at about 216 Ma and 203 Ma respectively (e.g., [57]), have been recently re-dated to about 228 Ma and to between 207–210 Ma respectively [58], [59]. These revised dates, which extend the duration of the Norian to about 20 Ma and that of the Rhaetian to 6 Ma or more, have had important implications for the age of the Chinle Formation.

Correlation of the Chinle Formation of PEFO to marine strata forming the basis for the Carnian, Norian, and Rhaetian stages, has been based primarily on pollen [60]–[62]. These pollen-based correlations have generally yielded a late Carnian age for the Blue Mesa Member, and a Norian age for the Petrified Forest Member, with the Carnian-Norian boundary being placed at about the level of the traditional Sonsela sandstone. These age determinations have been used to assign late Carnian and Norian ages to the Adamanian and Revueltian vertebrate faunas contained within the Blue Mesa and Petrified Forest ( = Painted Desert) Members respectively [54], [63]. The pollen correlations have received weak corroboration from isolated occurrences of vertebrate taxa in marine strata in Europe which are also known from Otischalkian (pre-Adamanian) and Revueltian faunas outside of the park (e.g., [63], [64].

However, recent magnetostratigraphic and radioisotopic data have revised these age assignments for the Chinle Formation. Channell et al. [65] and Muttoni et al. [66] used magnetostratigraphy to correlate strata within the Newark Supergroup to marine strata in Europe and Asia, and placed the Carnian-Norian boundary below the Lockatong Formation in the Newark Basin. Cornet [62] had previously used palynology to correlate the Lockatong Formation to both the Blue Mesa Member and Carnian marine strata in Austria. If the “Carnian” palynoflora of the Lockatong Formation is actually Norian in age, then the Carnian age for the Blue Mesa Member is also in doubt.

Recent radioisotopic dates suggest a Norian age for the Blue Mesa Member. Irmis and Mundil [67] provided a radioisotopic date for the base of the Blue Mesa Member in western New Mexico of 219.2±0.7 Ma. Riggs et al. [68] and Heckert et al. [69] obtained maximum ages of 213±1.7 Ma and 211±0.7 Ma respectively for the Black Forest Bed, near the top of the Petrified Forest Member. These dates, compared with the revised dates for the Carnian-Norian and Norian-Rhaetian boundaries, suggest that most, if not all of the Blue Mesa, Sonsela, and Petrified Forest Members are Norian, including that containing a “Carnian” palynoflora [67], and that the Owl Rock and Rock Point Members are mostly if not entirely Rhaetian.

## Materials and Methods

**Figure 6 pone-0009329-g006:**
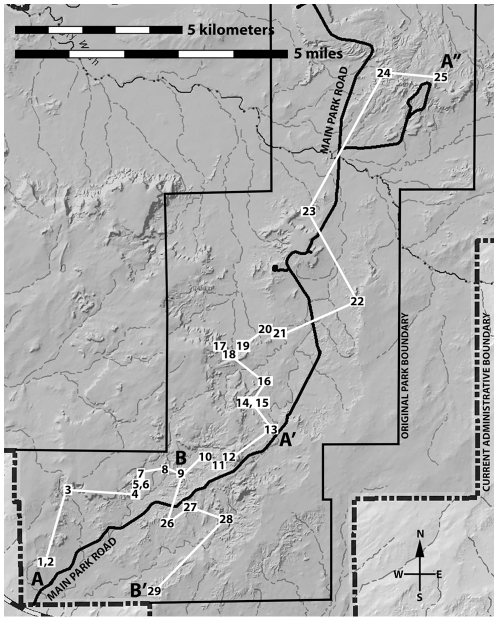
Map showing location of measured sections correlated in Figures 7–9. Sections described and illustrated in Appendix S1 and Figures S3–S10.

**Figure 7 pone-0009329-g007:**
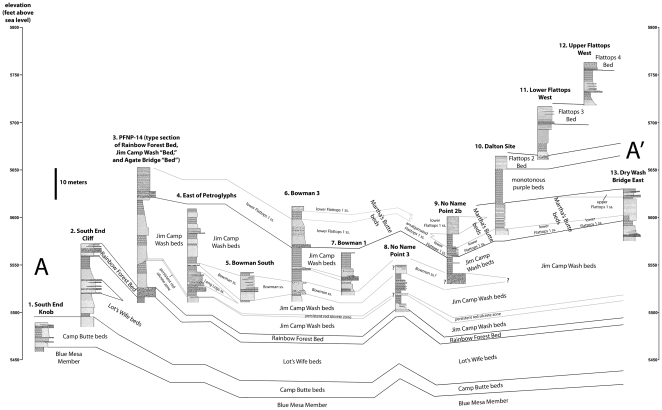
Transect A-A′. Correlation of measured sections 1–13.

**Figure 8 pone-0009329-g008:**
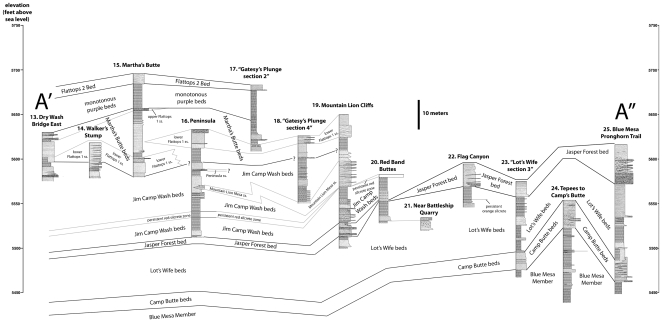
Transect A′-A″. Correlation of measured sections 13–25.

**Figure 9:**
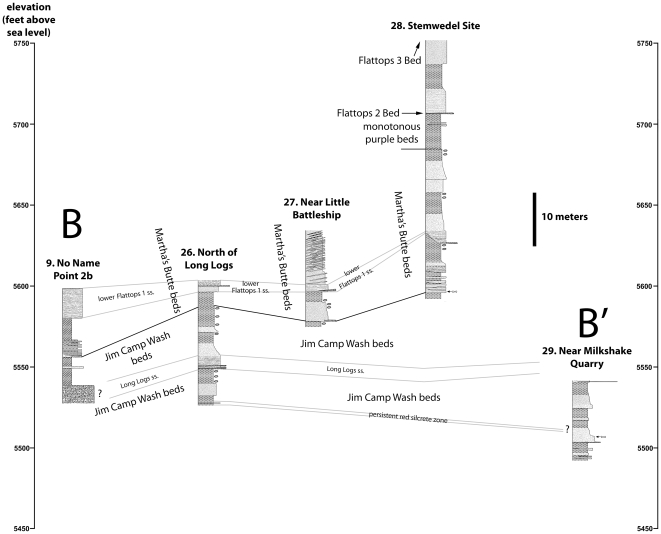
Transect B-B′. Correlating measured sections 9 and 26–29.

**Figure 10:**
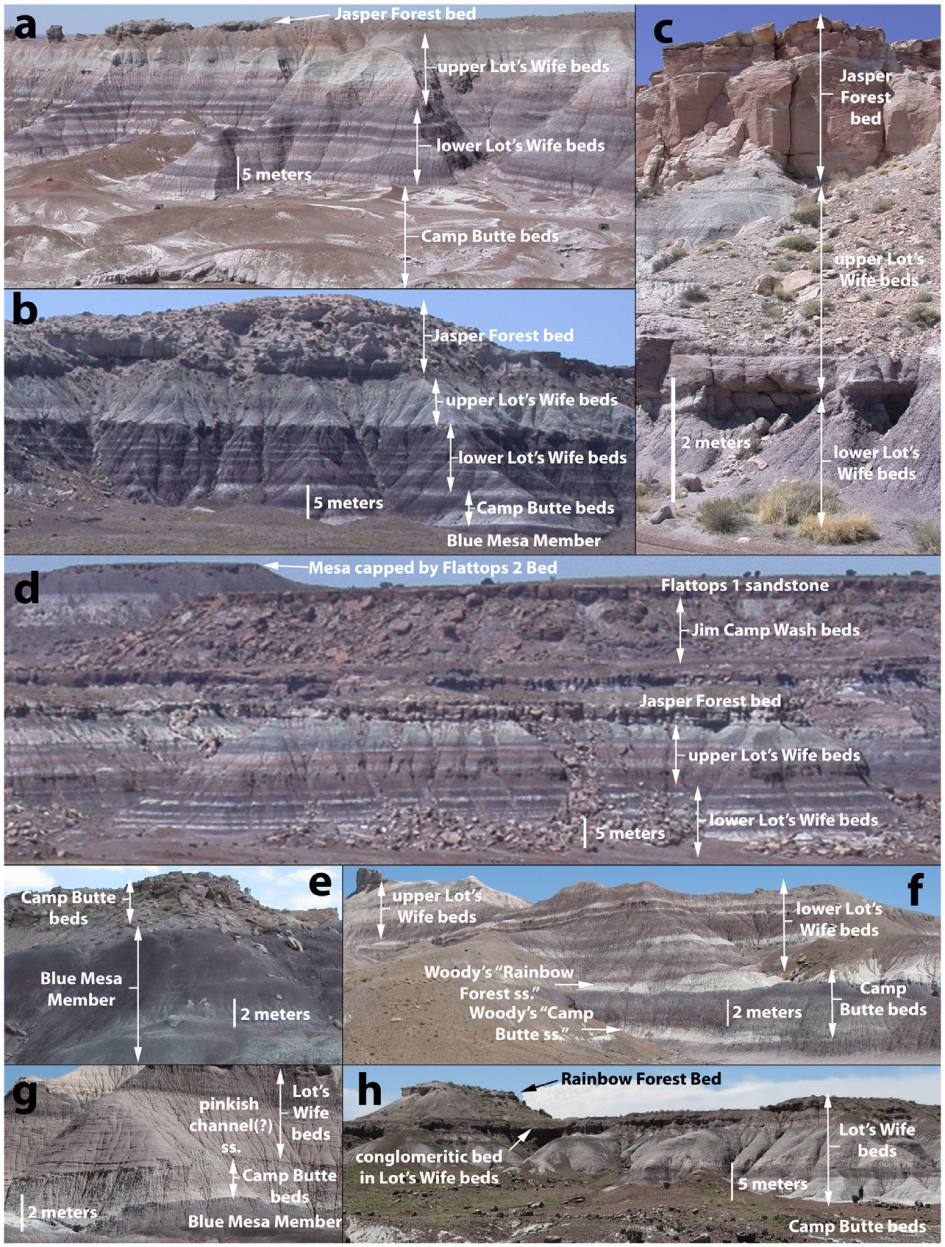
Lower part of the Sonsela Member. Section near Blue Mesa trail, photographed from trail overlook (a); North side of Agate Mesa near Lot's Wife section 3, photographed from about 12S E0610150 N3863360 NAD 27 facing south (b); Main Park Road roadcut on side of Agate Mesa just west of Agate Bridge at about 12S E0610390 N3862110 NAD 27 (c); Exposures west of Jasper Forest, just south of Point of Bluff, photographed from Jasper Forest overlook (d); Camp's Butte at 12S E0612547 N3867188 NAD 27 (e); Southern end of Lot's Wife at 12S E0610580 N3863210 NAD 27 (f); Exposures along south side of Blue Mesa taken 12S E0612674 N3866454 NAD 27 (g); Cliffs near south entrance station at 12S E0602063 N3851762 NAD 27 (h).

**Figure 11 pone-0009329-g011:**
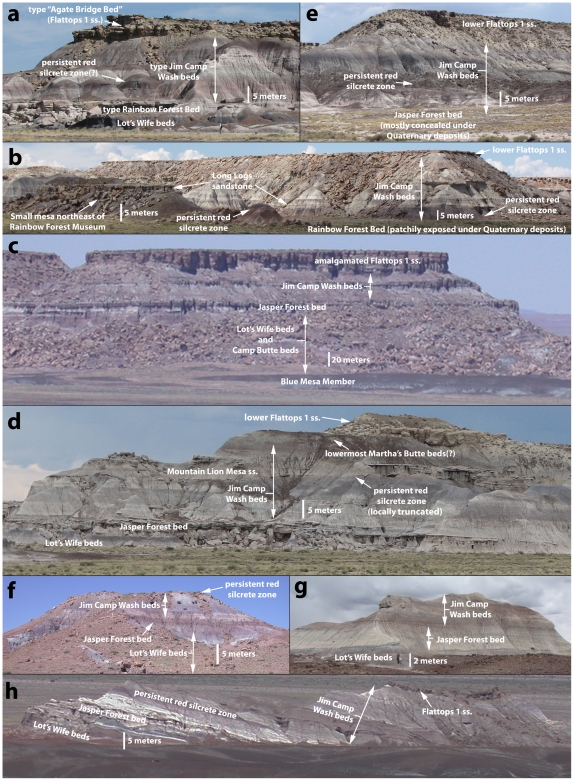
Jim Camp Wash beds. Heckert and Lucas' [11] type section of the “Agate Bridge Bed”, Jim Camp Wash beds, and Rainbow Forest Bed northwest of Giant Logs at 12S E0602800 N3854095 NAD 27 (a); Small mesa capped by Long Logs sandstone, and cliff where Roadifer [17] measured his PFNP-6 section, northeast of Rainbow Forest Museum, photographed from the main park road at 12S E0604460 N3853057 NAD 27 (b); Point of Bluff, photographed from the Jasper Forest overlook (c); Mountain Lion Cliffs section (also where Roadifer, [17] measured section PFNP-7) at 12S E0608065 N3858693 NAD 27 (d); Escarpment at “the Barrens” south of Crystal Forest on the east side of the Main Park Road at 12S E0609673 N3856770 NAD 27 (e); Red Band Butte section (also where Heckert and Lucas [11] measured their Hill 5573 section) photo taken from about 12S E0609050 N3859178 NAD 27 (f); The Battleship at 12S E0610410 N3858220 NAD 27(g); The Sinking Ship photographed from Blue Mesa overlook (h).

**Figure 12:**
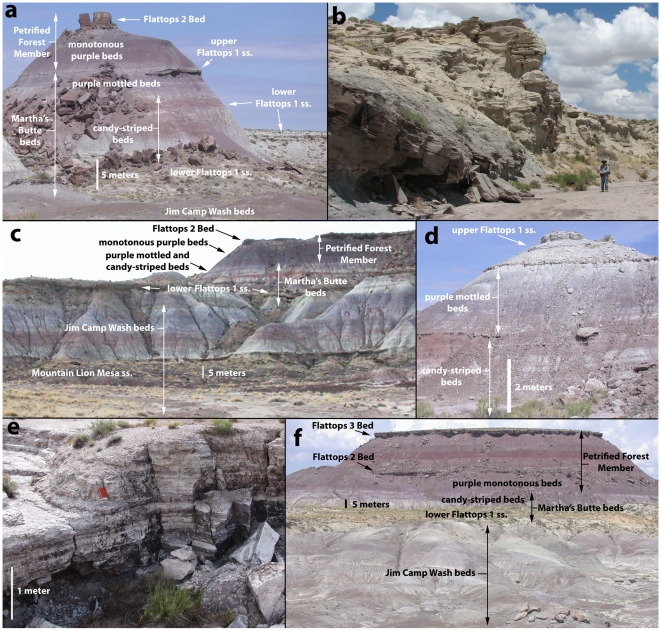
The Martha's Butte beds. Martha's Butte 12S 608235 3856775 NAD 27 with cliffs in the background capped by a lower Flattops One sandstone that is slightly higher than the one forming the base of Martha's Butte (a); Amalgamated Flattops One sandstone making up most of the Martha's Butte beds along Jim Camp Wash at 12S E0606245 N3855030 NAD 27 (b); exposures at Mountain Lion Mesa, the top of the section is Herrick's [52] “Gatesy's Plunge section 2” at 12S E0607320 N3858600 NAD 27 (c); Small mesa near Dry Wash bridge at 12S E0608848 N3855861 NAD 27 (d); Well-lithified siltstone possibly representing playa lacustrine deposits described by Espregen [70] near the Flattops at 12S E0607095 N3854893 NAD 27 (e); Red Butte just outside the traditional park boundary, and exposures of the Martha's Butte beds and Jim Camp Wash beds just inside the boundary along Jim Camp Wash, photographed from 12S E605599 N3854920 NAD 27 (f).

**Figure 13:**
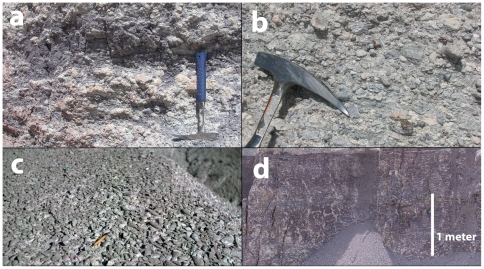
Paleosols and related features of the Sonsela Member. Pedogenic carbonate nodules in the Jim Camp Wash beds at the East of Petroglyphs section (a); Conglomeratic bed composed of reworked pedogenic carbonate nodules in Jim Camp Wash beds (b); Bed composed almost entirely of unionid bivalves in Jim Camp Wash beds (c); Vertic mottling in mudstones of the lower Lot's Wife beds at 12S E0605301 N3861618 NAD 27 (d).

**Figure 14:**
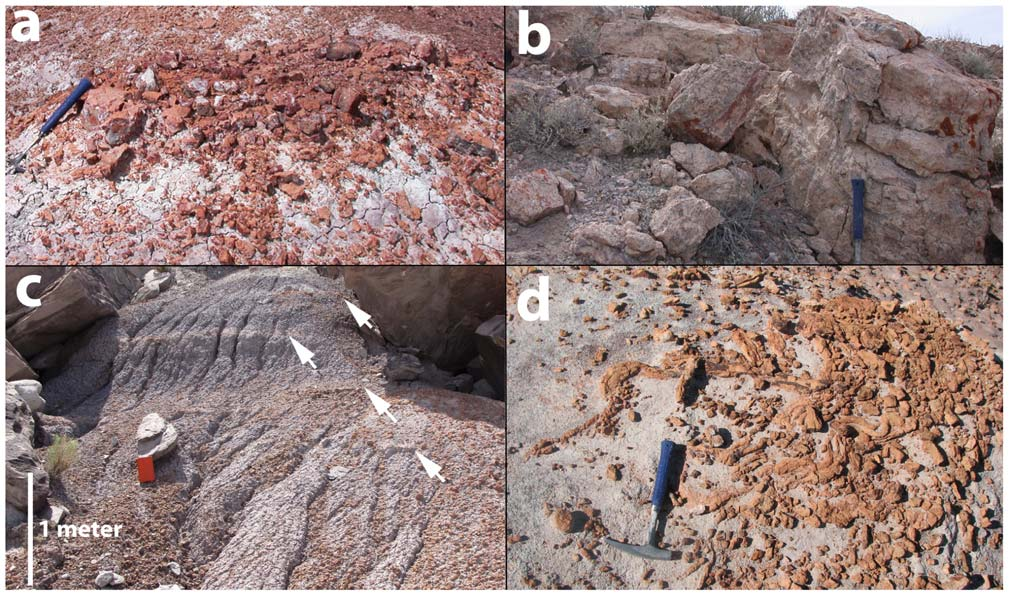
Silicified horizons in the Sonsela Member. Silcrete horizon in the persistent red silcrete zone at East of Petroglyphs section at 12S E0604665 N3854142 NAD 27 (a); Massive silcrete horizon in persistent red silcrete zone capping Red Band Butte at 12S E0608869 N3859185 NAD 27 (b); Multiple silcrete horizons (individual horizons marked by arrows) in persistent red silcrete zone near Roadifer's [17] PFNP-6 section at 12S E0604607 N3853944 NAD 27 (c); Persistent orange silcrete zone in Billing's Gap area showing distinct root traces at 12S E0616827 N3866023 NAD 27 (d).

The bulk of our efforts were devoted to carefully examining the lithostratigraphy of the Sonsela Member (*sensu* [11], [12]) in the southern region of Petrified Forest National Park (Figure 5). Our primary objective was to test the lithostratigraphic models of previous workers by establishing the precise superpositional relationships between various sandstone and mudstone-dominated units (Figures 6, 7, 8, 9, 10, 11, and 12), so that the superpositional relationships of vertebrate localities could likewise be established with as much precision as possible [70]. We have also identified some key lithologic features and traceable marker beds which help to characterize these units (Figures 13–14). Additionally, we have attempted to determine whether a single traceable erosional hiatus (the Tr-4 unconformity) really exists at the base of the Sonsela Member (*sensu* [11]), the base of the traditional Sonsela sandstone bed, or Flattops sandstone number one. We also have strived to improve the scientific testability of our lithostratigraphic model for the Sonsela Member through mapping (Figure S1) and the use of labeled outcrop photographs for all measured sections (Appendix S1, Figures S2, S3, S4, S5, S6, S7, S8, S9, and S10), methods that we feel are rarely employed with sufficient rigor.

In order to illustrate lithostratigraphic correlations explicitly and provide a basis for other researchers to test them, it is best to provide a visual record through mapping of how unit contacts were traced geographically. As noted by Raucci et al. ([29]p.157), “when a stratigraphy is constructed without comprehensive mapping, the tendency is to infer the distribution of key intervals based on correlations between stratigraphic columns, without fully confirming these correlations.” Geologic mapping ideally represents a claim by the researcher to have personally traced contacts on the ground, and to have confirmed visually that units have the geographic distribution necessary to make a lithostratigraphic model work. Tracing contacts in this manner also allows one to determine whether a single unconformable surface (such as the Tr-4) really exists throughout an entire area. A detailed geologic map was drawn for the particularly critical region between the Flattops and Jasper Forest (Figure S1).

Dividing up a measured section into different units is an inherently subjective enterprise. Moreover, there are frequently lateral changes in thickness and lithology within units, particularly in a complex fluvial system like the Chinle Formation. Consequently, identifying the units measured and described by a previous researcher on the outcrop is often extremely difficult. In additional to measured sections (Appendix S1), we provide labeled photographs of *all* outcrops where the sections were measured, clearly showing all the units we identified (Figures 6–9, Figures S2–S10). This removes all subjectivity for future workers attempting to identify units in our measured sections on the outcrop. We have also provided labeled photographs for measured sections of previous researchers we used in our correlations, with their units identified to the best of our ability.

## Results

The work of Heckert and Lucas [11] and Woody [12], while containing some errors in lithostratigraphic correlation, is nonetheless important in recognizing the presence of a thick package of sandstone-dominated strata in the middle of the Chinle Formation within Petrified Forest National Park that includes strata that previous workers included in the Blue Mesa and Petrified Forest Members. In accepting their assignment of strata to an expanded Sonsela Member, but correcting their correlations within this member, we present a model for an even thicker and more complex unit than recognized by any previous workers. Our revised model and correlations are summarized in Figures 5–9. This model recognizes five major packages of strata within the Sonsela Member, as opposed to the three of Heckert and Lucas [11] and Woody [12]. From lowest to highest these are: the Camp Butte beds (Figure 10), the Lot's Wife beds, Jasper Forest bed/Rainbow Forest Bed (Figures 10–11), the Jim Camp Wash beds, and the Martha's Butte beds (Figures 10–12). These lithostratigraphic revisions, combined with the recognition that Chinle deposition probably occurred almost entirely during the Norian and Rhaetian, has helped clarify the nature and timing of the vertebrate faunal transition [70], as well as changes in the depositional system and climate, within the Chinle Formation of Petrified Forest National Park. All sections referred to in the text are described and illustrated in Appendix S1 (Figures S2–S10) unless otherwise specified.

Our revised model hinges mainly on the superpositional relationships of the Jasper Forest bed and Flattops One sandstones. Therefore, the following discussion will begin by discussing the relationships between the Flattops One sandstones and Jasper Forest bed, and then the implications for other lithostratigraphic units, rather than proceeding in stratigraphic order from lowest to highest.

### The Flattops One Sandstones

Roadifer ([17]p.20–21) identified the “Camp Wash zone” (Flattops sandstone number one of Billingsley [18]) as a series of sandstone lenses that, although stratigraphically closely associated, nonetheless occur at slightly different stratigraphic levels. Espregen [71] also noted that several stratigraphically distinct sandstones were identified as Flattops sandstone number one. Our investigations have confirmed that Billingsley [49] mapped several sandstones at slightly different stratigraphic levels as Flattops sandstone number one, though all are above the Jim Camp Wash beds. Roadifer's identification of these beds as a “zone,” rather than as a single sandstone, was therefore telling. However, as the name “Camp Wash” has been formally appropriated for a different stratigraphic unit by Heckert and Lucas [11], the name “Camp Wash zone” should no longer be applied to the package containing these sandstones. We instead informally refer to this package above the Jim Camp Wash beds composed of interbedded resistant ledge-forming sandstone, friable slope-forming sandstone, and mudstone, as the “Martha's Butte beds” (Figure 5), and the resistant ledge-forming sandstones within this package as the “Flattops One sandstones.” Our primary reference section is at Martha's Butte (Figure S1, Figure 12a, see section in Appendix S1, Figures S6c,e), where three different Flattop One sandstones at slightly different levels occur in the same area.

Our tracing of the Martha's Butte beds on both sides of the Flattops indicates that all of the sandstones mapped as Flattops One sandstones by Billingsley [49] are either correlative with the type “Agate Bridge Bed” of Heckert and Lucas [11] northeast of Giant Logs (Figures 11a, and PFNP-14 section, Figures S3e–f), or slightly higher stratigraphically, and that all fall below the Flattops Two Bed. We informally refer to resistant sandstones occurring below the top of the Martha's Butte beds, including at the base as of the unit, as “lower Flattops One sandstones”, and to resistant sandstones at the top of the Martha's Butte beds as “upper Flattops One sandstones”. Distinguishing at which level the discontinuous lower Flattops One sandstones occur at relative to each other within the Martha's Butte beds is often very difficult, except at particular locations where several co-occur, such as at Martha's Butte. The discontinuous nature of the lower Flattops One sandstones causes some difficulty with tracing a precise contact between the Martha's Butte beds and Jim Camp Wash beds in particular areas, especially along the east side of Jim Camp Wash. We reject the use of the term “Agate Bridge Bed” for any of these sandstones for reasons explained in the next section.

The lithology of the Martha's Butte beds, particularly the Flattops One sandstones, was described by Roadifer ([17]; his “Camp Wash zone”), Espregen [71], Herrick ([52], at her “Gatesy's Plunge” study area), and Woody ([42], his “Facies G”). The Flattops One sandstones are commonly blocky weathering, yellowish, cliff-forming sandstones (Figures 11a–c, Figure S4c) that are texturally and compositionally immature. They often weather into a substrate particularly attractive to vegetation, and often stand out from stratigraphically lower and higher beds as yellowish sandstones covered with bushes (Figures 11d–e, Figure 12f). Conglomerates are composed primarily of reworked pedogenic carbonate clasts, although clasts of chert and quartzite are also common. Trough and planar cross-bedding are the most common sedimentary structures, although horizontal planar bedding and ripple cross-lamination is also present [12], [52], [71]. Lateral accretion bedding and “ridge and swale” topography also occurs; the distinctive scroll bar complex visible on the southwestern side of Flattops West from the main park road described by Woody ([12]fig.6) is in a lower Flattops One sandstone. Just south of Red Butte in the Jim Camp Wash drainage, and at Point of Bluff, lower Flattops One sandstones merge to form a massive amalgamated sandstone units more than 20 meters (60 feet) thick (Figure 11c, Figure 12b).

The slope-forming beds of the Martha's Butte beds are mostly sandy, and commonly exhibit the red and gray “candy-striping” described by Espregen [71] (Figure 12a,c–d,f). True mudstones also occur and are most commonly gray in color although they may also be dark purple, and are composed primarily of smectite [71]. Near the head of Starving Man Wash (Figure S1), the lower Flattops One sandstone capping the Peninsula, Starving Man Cliffs, and Mountain Lion Cliffs (Figure 11d, Figure 12c; see Peninsula and Mountain Lion Cliffs sections, Figures S7h-i, Figures S8a–b), grades laterally into dark gray mudstone, containing non-agatized petrified wood. Mudstones usually show evidence of pedogenic alteration [12], [52], and are commonly full of well-developed (plum to orange-sized) pedogenic carbonate nodules (Figure 13b). These are particularly well-developed in a zone at the very top of the Martha's Butte beds referred to informally as the “purple mottled beds” (Figure 12a,c–d see Martha's Butte and “Gatesy's Plunge 2” sections, Figures S6c,e, Figures S7f,g). Between the main park road and the Peninsula, these purple mottled beds are usually separated from the rest of the Martha's Butte beds by an erosional contact (Figure 12d). In one confined area on the west side of the West Flattops, on the north side of the main park road, there is a thick sequence of well-lithified purple and gray mottled siltstone (Figure S1, Figure 12e) showing considerable variation in thickness, lying low in the Martha's Butte beds just above the lower Flattops One sandstone. This unit was identified by Espregen ([71]p. 73–90) as a playa lake deposit.

### The Stratigraphic Relationship of the Flattops One Sandstones to the Jasper Forest Bed (Traditional Sonsela Sandstone Bed)

Following most previous workers (e.g., [5]p. 15,[12], [16], [17], [19], [20], [44]) we accept the correlation of the conglomeratic sandstones capping Blue Mesa (Figure 10a), Agate Mesa (Figures 10b–c), and the bluffs north of Crystal Forest (Figure S1, Figure 5a, Figure 8, see Flag Canyon, “Lot's Wife 3”, and Blue Mesa Pronghorn sections, Figures S8f–g, Figures S9a–b, e–f). This is due to the lithologically distinctive nature of the traditional Sonsela sandstone bed itself, and also the distinctive stratigraphic sequence exposed below it identified erroneously (as will be discussed later) as the Jim Camp Wash beds by Heckert and Lucas [11] and Woody [12]. This is why we disagree with Heckert and Lucas' [11] correlation with the sandstone capping Blue Mesa with those at the base of Agate Mesa. The two mesas in fact expose roughly the same stratigraphic interval (Figures 4a–b, d, 5a); the sandstones capping these mesas (the traditional Sonsela sandstone) are correlative, as are the sandstones at their bases (discussed below). We also agree with the identification by Roadifer ([17], his section PFNP-10) and Woody ([42], his sections Agate Mesa West 1 and 2) of the sandstone capping the lower tier of bluffs a kilometer west of Jasper Forest is also the Sonsela sandstone bed as these cliffs also clearly expose the same distinctive section (Figure 10d).

From the cliffs west of Jasper Forest, the Sonsela sandstone bed is easily traced south to where it crops out at the base of the Mountain Lion Cliffs (Figure S1, Figure 5a, Figure 11d, see Mountain Lion Cliffs section, Figures S8a–b). There, it clearly lies about 20 meters below a Flattops One sandstone capping the cliffs, as previously noted and mapped by Roadifer [17], Billingsley [49], and Herrick [52]. Woody ([12]fig. 4.2) erroneously figured the Sonsela sandstone bed here as a sandstone “tier” within the Jim Camp Wash beds. West of Jasper Forest, where the cliffs capped by the Jasper Forest bed approach Point of Bluff, a Flattops One sandstone forms a higher tier of cliffs and the Jasper Forest bed locally thins in the cliff side, briefly pinches out just south of Point of Bluff, but then reappears to form the lowest of the major ledge-forming sandstones exposed at Point of Bluff itself (Figure 11c). On the west side of the main park road, the traditional Sonsela sandstone bed can be traced across flats south of Crystal Forest to the area called “the Barrens”, where it dives to the base of an escarpment capped by a lower Flattops One sandstone (Figure S1, Figure 11e).

Tracing the traditional Sonsela sandstone bed below Flattops One sandstones (Figure 5) contradicts the claims of Heckert and Lucas [11] and Woody [12] that these units are correlative. This is unfortunate as it means Heckert and Lucas' [11] type section for their “Agate Bridge Bed” (Figure 11a, Figures 16e–f, a Flattops One sandstone) is not only several kilometers from the petrified log natural bridge called Agate Bridge, but stratigraphically much higher than the sandstone capping Agate Mesa that contains the log (the traditional Sonsela sandstone). For this reason, we do not apply the name “Agate Bridge Bed” to any Flattops One sandstones. However, the expansion of the term “Sonsela” by Heckert and Lucas [11] to include a thicker package of interbedded sandstone and mudstone still requires a new name for the traditional Sonsela sandstone bed, which only occupies a part of this interval, as a lithostratigraphic unit may not bear the same name as a part of it (North American Stratigraphic Code, Article 19f). Although, for the sake of simplicity, we would prefer to retain the name “Agate Bridge Bed” by reassigning the type section to the traditional Sonsela sandstone bed, it is unfortunately also not permissible to relocate the type section of a lithostratigraphic unit (NASC, Article 22c). The name “Agate Bridge Bed” and its type section must therefore be abandoned for what we consider “widespread misuse in diverse ways that compound confusion” (NASC, Article 20a). We propose substituting Raucci et al.'s [29] term “Jasper Forest bed” as an informal name for the traditional Sonsela sandstone bed north of the Flattops, with main reference section being the capping sandstone at Agate Mesa, best exposed on the northern face (Figure 10b, see “Lots Wife section 3”, Figures S9a–b).

In addition to stratigraphic separation, there are lithologic differences between the Flattops One sandstones and the Jasper Forest bed. In fact, Woody [12] identified two distinct lithologic facies within the “Agate Bridge Bed,” and his descriptions of these facies, as well as his locality photographs, makes clear that “Facies F” is the Jasper Forest bed, while “Facies G” is the Flattops One sandstones ([12]figs.5–7). The lithology and sedimentology of the Jasper Forest bed has been extensively described (e.g., [12], [16], [17], [42], [45], [52]). The unit is composed of texturally mature, extremely siliceous conglomeratic sandstone, where the gravel-sized clasts are dominated by extrabasinal chert (silicified Paleozoic limestone), quartzite, and reworked volcanic clasts of Triassic age [72], and there is abundant well-preserved reddish and multicolored “jasperized” petrified wood preserved by replacement (see [73]p.54). The sand bodies are usually multi- storied, and the dominant bedform is planar cross-bedding, with lesser trough cross-bedding and horizontal planar-bedding. In contrast, the Flattops One sandstones are texturally and compositionally immature sandstones, where conglomerate tends to be a relatively minor component, gravel clasts are dominated by re-worked intrabasinal pedogenic carbonate (though the sandstones and conglomerates are both still very siliceous compared to the Jim Camp Wash beds), individual sand bodies are mostly single-storied, and the petrified logs are white or orange “non-jasperized” wood preserved by permineralization ([73]p. 54).

### The Stratigraphic Relationship of the Jasper Forest Bed (Traditional Sonsela Sandstone Bed) and Rainbow Forest Bed

The Jasper Forest bed and Rainbow Forest Bed have long been recognized as extremely important stratigraphic marker beds within the PEFO. Most of the major studies have identified the Rainbow Forest Bed and Jasper Forest bed as being slightly stratigraphically distinct [11], [12], [16]–[20], [29], [40], with many of these workers claiming to be able to identify the Rainbow Forest Bed and Jasper Forest bed occurring together as stratigraphically distinct units either west or north of the Flattops.

However, it seems more likely that the Jasper Forest bed and Rainbow Forest Bed are stratigraphically equivalent as advocated by several workers over the years [17], [20], [44]–[46]. Heckert and Lucas [11], although they erroneously correlated the sandstone capping Blue Mesa with that at the base of Agate Mesa, were correct in correlating the former with the Rainbow Forest Bed. The Jasper Forest bed and Rainbow Forest Bed are lithologically almost identical, being siliceous conglomeratic sandstones with gravel dominated by silicified Paleozoic limestone and volcanic clasts, and containing dark red and multi-colored agatized petrified wood. These characteristics distinguish these beds from all other sandstone units in the Chinle Formation within the park. Both lie about the same stratigraphic distance below the Martha's Butte beds/Flattops One sandstones on either side of the Flattops (about 25–30 meters; see Figure 5, Figures 11a–b, Figures d–e and PFNP-14, East of Petroglyphs, Peninsula, and Mountain Lion Cliffs sections, Figures S3e–h, Figures S4c–d, Figures S7a–e, Figures S8a–b). Moreover, there is a distinctive reddish silicified horizon a few meters above both units (discussed below).

The Jasper Forest bed is mostly complete where it forms the caps on Blue Mesa, Agate Mesa, and the cliffs north of Crystal Forest and west of Jasper Forest, but the top of the unit is nonetheless eroded and draped in Quaternary deposits. Where it forms these resistant ledge-forming caps, the Jasper Forest bed is at its thickest (5–10 meters or more), and has thick conglomeratic beds, especially in the lower part of the unit (Figures 10a–d, sections for “Lot's Wife section 3”, Blue Mesa Pronghorn, and Flag Canyon, Figures S8f–g, Figures S9a–b,e–f). Further west at Ramsey Slide and Twin Buttes, the Jasper Forest bed becomes massive conglomeratic sandstone with cobble-sized clasts [45], which includes volcanic clasts that are Triassic in age [72].

In contrast to the usually cliff-capping Jasper Forest bed, the Rainbow Forest Bed is mostly exposed at close to ground level throughout south of the Flattops in the area north of Rainbow Forest and in the drainages of Jim Camp Wash and Cottonwood Wash. This might partially account for why most workers considered it stratigraphically lower than the Jasper Forest bed, and why Heckert and Lucas [11] correlated the latter with the cliff-capping Flattops One sandstones south of the Flattops. However, tracing the Rainbow Forest Bed south from Heckert and Lucas' [11] type locality (Figures 6–7) reveals that it rises to cap the bluffs west of Long Logs (Figure 11h, South End Cliff section, Figures S3c–d), as recognized by Billingsley [49]. This is contra Roadifer [17] and Woody [42], who mistakenly identified this bluff-capping sandstone as a Flattops One sandstone. These bluffs are the only area south of the Flattops where the lower part of the Sonsela Member (the Lot's Wife beds and Camp Butte beds, discussed below) are well-exposed.

Although they are probably stratigraphically equivalent, it is not known for certain that the Jasper Forest bed and Rainbow Forest Bed are physically continuous, given the inherently discontinuous nature of fluvial sand bodies, and the fact that neither unit can be traced across or around the Flattops. Moreover, there are distinct facies changes in the Jasper Forest bed south of Agate Mesa as it approaches Mountain Lion Mesa west of the main park road, and in the Crystal Forest area east of the main park road. The facies change at the base of Mountain Lion Cliffs was noted by both Roadifer [17] and Herrick [52]. Here, the Jasper Forest bed thins and becomes a “hoodoo”-weathering sandstone with relatively little conglomerate (Figure 11d, see Mountain Lion Cliffs section, Figures S8a–b) which disappears into the subsurface further south. At Red Band Buttes (Figure 11f), the Jasper Forest bed almost completely pinches out, and forms only the reddish bed which gives the buttes their name (see Red Band Buttes section, Figure S1, Figures S8c–d). A similar facies change is also observed east of the main park road in the Crystal Forest area, where the Jasper Forest bed becomes a friable (though still locally conglomeratic) sandstone which caps the low hills of Crystal Forest itself, and forms most of the section at the Battleship (Figure S1, Figure 11g). These facies changes may indicate that this is the edge of the channel system that produced the Jasper Forest bed, and that it may pinch out beneath the Flattops.

### The Stratigraphic Relationship of the Rainbow Forest Bed and the Camp Butte Beds

The name “Rainbow Forest beds” was coined by Woody [12] for sandstones exposed at the base of the Sonsela Member at Agate Mesa, Blue Mesa, and the surrounding area. Woody [12] considered these beds to be composed of two sandstone lenses. He used the name “Camp Butte sandstone,” following Long and Murry ([4]p. 214) for one of these sandstones, which was previously identified (but not named) by Murry and Long [74] and Murry [20] capping Camp's Butte just west of Blue Mesa (Figure 2, Figure 10e, see Tepees to Camp's Butte section, Figures S9c–d). Woody [12], [42] claimed this sandstone could be traced throughout the area, and identified a lens of white sandstone pinching out on the north end of Lot's Wife (Figure 10f) as its southern termination. Woody [42] identified the second sandstone making up the “Rainbow Forest beds” as the “Rainbow Forest sandstone”, and identified it at Lot's Wife as another white sandstone lens pinching out to the north, a few meters above the lens he identified as the Camp Butte sandstone.

We agree with Heckert and Lucas [11] and Woody [12] that this distinctive package of pale sandstone and conglomerate interbedded with mudstone, including the conglomeratic sandstone capping Camp's Butte, should mark the base of the Sonsela Member. However, we disagree with the precise local correlations of individual sandstone lenses within this package advocated by Woody [12]. This package, and the uppermost Blue Mesa Member below it, are especially well exposed around Blue Mesa, the north side of Agate Mesa, and Lot's Wife (Figures 10a–b,f, see “Lot's Wife section 3” and Blue Mesa Pronghorn sections, Figures S9a–b,e–f). However, further south this package if often partly or entirely concealed by Quaternary deposits. It is also removed by erosion or buried by Quaternary deposits in between these geographic features. It is therefore extremely difficult, if not impossible, to trace individual sand bodies within this package with confidence. This is especially true of the sandstone Woody [12] identified at Lot's Wife as the “Rainbow Forest sandstone,” which cannot even be traced continuously all the way around Agate Mesa, much less southwest of the Flattops. These sandstones are also certainly stratigraphically lower than the Rainbow Forest Bed, because (discussed above) the Rainbow Forest Bed and the Jasper Forest bed capping Agate Mesa and Blue Mesa are stratigraphically equivalent.

Furthermore, tracing individual sandstones within the “Rainbow Forest beds” at Blue Mesa and Agate Mesa reveals that it is an even more complex package than described by Woody [12]. For example, the multi-storied conglomeratic sandstone exposed at Camp's Butte can only be traced about a half kilometer to the south, where it thins out into the overlying strata (the Lot's Wife beds, discussed below), and another white sandstone lenses in below it on the south side of Blue Mesa. Moreover, just south of Lot's Wife, another sandstone lenses in and becomes a thick and resistant conglomeratic unit at about the same stratigraphic level as the lens pinching out at Lot's Wife that Woody [12] identified as the Camp Butte sandstone. For these reasons, we prefer to simply treat Woody's “Rainbow Forest beds” as a package of discontinuous but closely associated sandstones and conglomeratic sandstones interbedded with the uppermost Blue Mesa Member and lower Lot's Wife beds (Figure 5). As the name “Rainbow Forest beds” is stratigraphically misleading, we refer to this package as the “Camp Butte beds”.

The Camp Butte beds have been described in particular by Woody ([12], his “Facies B”) and Herrick ([52], her “Facies A” at her “Lot's Wife” locality on the north side of Agate Mesa). The unit is composed of light-colored compositionally and texturally mature siliceous sandstone dominated by trough cross bedding with lesser planar cross-bedding and horizontal planar bedding. The unit is locally conglomeratic with gravel-sized clasts composed mostly of reworked mudstone from the Blue Mesa Member, although chert and even (locally at King's Throne) volcanic clasts may be present [12]. It therefore has lithologic similarities with the Jasper Forest bed/Rainbow Forest Bed.

The sand bodies locally consist of single-storied lenses, individually usually not more than a meter thick, interbedded with the Blue Mesa Member and Lot's Wife beds (Figure 10f). Around Point of Bluff along the western park boundary, and near the southern entrance to the park, the Camp Butte beds are only few meters thick (see South End Knob section, Figures S4a–b, and unit 1 in Roadifer's [17] PFNP-11 section). Locally, they form a more massive multi-storied ledge-forming conglomeratic sandstone 5–10 meters thick (Figure 10e; see “Lot's Wife section 3”, Tepees to Camp's Butte, and Blue Mesa Pronghorn sections, Figure S9). At King's Throne the Camp Butte beds are an unusually well-cemented ledge-forming conglomerate, with particularly massive (cobble-sized) clasts often exceeding 10 cm in diameter [42].

### The Jim Camp Wash Beds and Lot's Wife Beds

Having identified the Flattops One sandstones, Jasper Forest bed/Rainbow Forest Bed, and Camp Butte beds as all being stratigraphically distinct, it becomes clear that the strata referred to as the “Jim Camp Wash beds” by Heckert and Lucas [11] and Woody [12] actually occur at two separate stratigraphic levels (Figure 5, Figure 7–9). The type section of the Jim Camp Wash “Bed” (Figure 11a, see PFNP-14 section, Figures S3e–f) was designated by Heckert and Lucas ([12]; their “Giant Logs” section) near the extreme south end of the park, and lies above the Rainbow Forest Bed (the type section of which is at the same locality). These strata can be traced along the cliffs north of Rainbow Forest, and around the drainages of Jim Camp Wash and Cottonwood Wash. In this area, the total thickness of the Jim Camp Wash beds is about 25–30 meters thick although the very base of the unit is only intermittently exposed (Figure 11b; see East of Petroglyphs, Bowman sections, No Name Point 2b, No Name Point 3, North of Long Logs, and Near Milkshake Quarry sections, Figures S3g–h, Figure S4, Figures S5a–b, Figures S10a–b,h–i).

This same package of sediment reappears below the Martha's Butte beds north of the Flattops along the main park road (Figure S1, Figure 11e, Figure 12a; see Dry Wash Bridge East and Martha's Butte sections, Figure S6), and can be traced north along the east facing escarpment below Starving Man Cliffs and Mountain Lion Cliffs (Figure S1, Figure 8, Figure 11d; see Peninsula section, “Gatesy's Plunge section 4”, and Mountain Lion Cliffs section, Figures S7a–e,h–i, Figures S8a–b), and form the area of badlands called the “Wastelands” just north of Mountain Lion Cliffs (Figure S1, Figure 2). North of here, the exposures of the Jim Camp Wash beds move west of the traditional park boundary, but reappear inside the park at Point of Bluff (Figure 10d, Figure 11c).

As noted by Roadifer [17] and Woody [12], the boundary between the top of the Jim Camp Wash beds and the base of the Martha's Butte beds can be difficult to place, given that both are fairly complex units of interbedded resistant ledge-forming sandstones and friable slope-forming sandstones and mudstones, with numerous incised contacts between these units. However, the often blocky-weathering tan and yellowish Flattops One sandstones (Figures 11a–b,d, Figures S4c–d) are distinct from the resistant sandstones of the Jim Camp Wash beds, which tend to be grayish and less-resistant “hoodoo” weathering (Figures 11a–b,d). Also, as noted by Woody [12], there is often a subtle color shift in the slope forming sandstones and mudstones of these units from more purplish (in the uppermost Jim Camp Wash beds) to more grayish (in the Martha's Butte beds). Even so, the transition is particularly difficult to identify on the east side of the Jim Camp Wash drainage, where some of the sandstones of the Martha's Butte beds lack the distinctive blocky weathering seen elsewhere. The contact between the Jim Camp Wash beds and Martha's Butte beds was identified here by carefully tracing the blocky tan-colored lower Flattops One sandstone representing the type of Heckert and Lucas' [11] “Agate Bridge Bed” all the way from their “Giant Logs” type section east to Jim Camp Wash, and around the Jim Camp Wash drainage (Figures 6–7, and discussion for No Name Point 2b and North of Long Logs sections in Appendix S1). In this area, and continuing northeast of Long Logs, the lower part of the Martha's Butte beds grades into friable and muddy yellowish-gray sand (see Near Little Battleship section, Figure S10c–e), and eventually grades into mudstones with interbedded sandstones indistinguishable from the Jim Camp Wash beds (see North of Long Logs and Stemwedel Site sections, Figures S10a–b,f–g).

The strata making up most of the section exposed at the sides of Blue Mesa, Agate Mesa, Lot's Wife, King's Throne, the cliffs north of Crystal Forest, and those west of Jasper Forest (Figure S1, Figures 10a–d,f; see Flag Canyon, “Gatesy's Plunge section 3”, Blue Mesa Pronghorn Trail sections, Figures S8f–g, Figures S9a–b,e–f), are exposed below the Jasper Forest bed and Rainbow Forest Bed, and therefore require a new name. We suggest the name “Lot's Wife beds”. In contrast to Heckert and Lucas [11], we prefer to refer to both the Jim Camp Wash beds and Lot's Wife beds as informal units following Woody [12], as we also do with the Camp Butte beds and Martha's Butte beds, given that these are thick and highly heterogeneous packages of strata in terms of lithology and sedimentary architecture, and it makes little sense to formalize them as a single “Bed”.

The Lot's Wife beds generally have a thickness of 15–20 meters at Blue Mesa (Figure 10a, Blue Mesa Pronghorn section), Agate Mesa (Figure 10b, “Lot's Wife Section 3”), and north of Crystal Forest, but are thicker to the west, reaching 30 meters at the cliffs west of Jasper Forest and around Point of Bluff (Figure 11c, Figure 10d; see also Woody's [42] Agate Mesa West 1 and Agate Mesa West 2 sections, and units 2–4 of the PFNP-11 section in Roadifer [17]).

As with the “Agate Bridge Bed,” Woody [12] identified two distinct facies as being part of the Jim Camp Wash beds, and his outcrop photos make clear that his “Facies D” ([12]fig. 4.1) is the Lot's Wife beds, while his “Facies E” ([12]figs.4.2,5) is the Jim Camp Wash beds. This is corroborated by the differences between these facies that he describes, which is consistent with our own observations on how the Lot's Wife beds and Jim Camp Wash beds differ. These units were also described by Herrick [52], with “Facies B, C, and E” at her “Lot's Wife” locality representing the Lot's Wife beds, and “Facies F” and (in part) “Facies E” at her “Gatesy's Plunge” locality representing the Jim Camp Wash beds.

Both the Lot's Wife beds and Jim Camp Wash beds are complex units of interbedded sandstone, conglomerate, and mudstone exhibiting cut and fill architecture [11], [12]. Sand bodies in both units are ribbons and sheets, with the latter often connecting the former in tiers, sands are usually texturally and compositionally immature lithic wackes, and conglomerates (when present) are composed primarily of re-worked sedimentary clasts, especially re-worked pedogenic carbonate nodules [12], [52]. However, the sand bodies in the Jim Camp Wash beds tend to be thicker and more laterally continuous, mudstone is a relatively minor component and more variable in color compared to the Lot's Wife beds. Also, pedogenic carbonate nodules, some reaching 10 cm or more in diameter, as well as conglomeratic lenses composed of reworked pedogenic carbonate, and dense accumulations of unionid bivalves (Figures 13a–c), are abundant in the Jim Camp Wash beds but virtually unknown in the Lot's Wife beds.

North of the Flattops, the Lot's Wife beds can be loosely divided into lower and upper beds (Figures 10a–d,f–g, see “Lot's Wife section 3” and Blue Mesa Pronghorn section, Figures S9a–b,e–f). Horizontal beds of interbedded purple mudstone (Figure 13d) and pale sheet sandstones dominate the lower Lot's Wife beds (Woody [12] described these sheet sandstones as “tiers; see his fig. 4.1). The upper Lot's Wife beds are dominated by light gray and reddish sandstone, with purple and bluish mudstones and muddy sands being a minor component. Sandstones in the upper Lot's Wife beds are sometimes particularly resistant ledge-forming units (Figure 10c), especially near the contact between the upper and lower Lot's Wife beds (this is especially true at the Battleship Quarry section, where Heckert and Lucas [11] identified ledge-forming sandstones at this contact as the Rainbow Forest Bed in their “Hill 5573” section). In other places, the upper Lot's Wife beds may be fairly muddy and friable (Figure 10b, see “Lots Wife section 3”, Figures 22a–b). Murry ([20]p.785) identified sandstones occurring only five meters below the Jasper Forest bed at Crystal Forest as being correlative with the Camp Butte sandstone. These are actually part of the upper Lot's Wife beds (although the Camp Butte beds are well exposed lower in the section further north in badlands exposed at the north end of the cliffs; Figure S1).

This distinction between the lower and upper Lot's Wife beds is not always clear; pinkish medium-to coarse-grained cross-bedded sandstones locally dominate the lower Lot's Wife beds (one of these is Herrick's “Facies E” at her “Lot's Wife locality”), and even incise into the top of the Camp Butte beds (Figure 10g). In other places, individual cut and fill sequences in the upper Lot's Wife beds fine up into dark purple and reddish brown mudstones and muddy sands, making them difficult to distinguish from the lower Lot's Wife beds. Moreover, there are locally interfingering contacts between the lower and upper Lot's Wife beds, and between the lower Lot's Wife beds and Camp Butte beds. Nonetheless, the stratigraphic distinction between the Camp Butte beds, lower Lot's Wife beds, and upper Lot's Wife beds is common and striking in this region of the park.

### Stratigraphically Significant and Traceable Sandstone Units within the Jim Camp Wash Beds

Some of the resistant sandstone beds within the Jim Camp Wash beds are noteworthy (Figure 5b) because they can be at least locally traced and mapped, and because some have been (erroneously) identified as the Jasper Forest bed or Rainbow Forest Bed. Cooley ([16]p. 93) identified the traditional Sonsela sandstone bed (Jasper Forest bed) in the Rainbow Forest area with sandstones and conglomerates distinct from, and slightly up section from, the Rainbow Forest Bed. Specifically, Cooley identified a sandstone capping a “small mesa one mile northeast” of the Rainbow Forest Museum (probably the one shown in Figure 11b) as the Jasper Forest bed (Billingsley [49] erroneously mapped the sandstone capping this small mesa as the Rainbow Forest Bed, which is actually exposed at its base). Cooley noted that this sandstone differed from the Jasper Forest bed north of the Flattops in being a fine- to medium-grained non-conglomeratic sandstone grading laterally into siltstone. In this same area, Murry ([20]p. 785) also identified the Jasper Forest bed as a thin, well-consolidated siliceous sandstone about 5 meters above the Rainbow Forest Bed.

Neither Cooley [16] nor Murry [20] explained why they identified these sandstones as the Jasper Forest bed. Both, however, were both referring to discontinuous resistant sandstones lenses lying at close to the same stratigraphic level, low in the Jim Camp Wash beds, a few meters above the persistent red silcrete zone (discussed below). These sandstones are lithologically very distinct from the Jasper Forest bed in being dominated by intrabasinal clasts composed of reworked pedogenic carbonate nodules, whereas conglomeratic clasts in both the Jasper Forest bed and Rainbow Forest Bed are dominated by extrabasinal chert, quartzite, and volcanic rocks.

We refer to the sandstone capping the small mesa northeast of the Rainbow Forest Museum, which is one of the thickest and most resistant of these sandstones and an important ledge-forming unit in the Jim Camp Wash drainage, as the “Long Logs sandstone” (Figure 11b, and see North of Long Logs and East of Petroglyph sections, Figures 16g–h, Figures S10a–b). This is probably the same sandstone called the “Agate House Bed” by Heckert [50], although this is unclear as he never discussed this unit in the text. Another light gray hoodoo-forming sandstone is present at the Bowman vertebrate locality, and lies just above the Long Logs sandstone (see East of Petroglyph and Bowman Site sections, Figures S3g–h, Figures S4a–f); we refer to this as the “Bowman sandstone.” A sandstone capping many of the small mesas in the Jim Camp Wash drainage is roughly at this same level (Figure S1, see No Name Point 3 section, Figures S4g–h). Resistant and locally traceable sandstone lenses are present higher in the Jim Camp Wash beds in this area, although we do not provide names for them.

North of the Flattops, two locally traceable sandstone units were named in the Jim Camp Wash beds in the area between the Peninsula and Mountain Lion Mesa. The “Peninsula sandstone” is a thin (less than 1 m thick) ledge-forming flaggy sandstone with interbedded conglomerate, lying fairly high in the Jim Camp Wash beds, which can be traced around the northeast end of the Peninsula (Figure S1, see Peninsula section, Figures S7a–e). On the southeastern side of the Peninsula, it dives into the subsurface before reaching Martha's Butte. North of the Peninsula, it can be traced with slight difficulty across the flats below the Starving Man Cliffs north as far as Starving Man Wash. A similar conglomeratic bed crops out about the same distance below the lowermost Flattops One sandstone further south (Figure S1, see Dry Wash Bridge North section, unit 2 in Figures S6a–b), and is probably more or less correlative.

The “Mountain Lion Mesa sandstones” are a series of connected blanket sands lying slightly below the level of the Peninsula sandstone. The lowest of these is a resistant, “hoodoo”-weathering unit exposed at the Peninsula (Figure S1, see Peninsula section, Figures S7c,e). To the south, it becomes a well-cemented unit largely concealed under Quaternary alluvium, but to the north the Mountain Lion Mesa sandstone forms a more massive and resistant tan-colored sandstone which is intermittently exposed and can be traced to the base of Mountain Lion Cliffs. Here, it thins to a light pink layer at the base of a slightly higher Mountain Lion Mesa sandstone (which may be close to the same level as the Peninsula sandstone) that can be traced along the Mountain Lion Cliffs and Mountain Lion Mesa (Figure S1, Figure 11d, Figure 12c, see “Gatesy's Plunge section 4” and Mountain Lion Cliffs sections, Figures S7h–i, unit 3c in Figures S8a–b). This upper Mountain Lion Mesa sandstone is a multistoried and architecturally complex sand body that was described by Herrick ([52]p.12) at the base of “Facies F” at her “Gatesy's Plunge” locality. Parker and Irmis [75] mistakenly identified this sandstone as the Rainbow Forest Bed at the type locality for the phytosaur *Pseudopalatus jablonskiae*.

Roadifer ([17]p.18–20) identified the Rainbow Forest Bed as being identifiable north of the Flattops, above the Jasper Forest bed. Specifically, he identified the Rainbow Forest Bed with a “pebbly quartzose sandstone bed very similar to the Sonsela…[that] occurs about 20 feet above the Sonsela” in the exposures along the northeastern flanks of Mountain Lion Mesa. Roadifer [17] may have been referring to the one of the Mountain Lion Mesa sandstones.

### Stratigraphically Significant Silcrete Horizons in the Sonsela Member

Silicified horizons are common in the Sonsela Member. One stratigraphic interval generally less than two meters thick in which these horizons frequently occur lies near the base of the Jim Camp Wash beds (Figures 14a–c). Woody [12], [42] indicated that these silcretes are about 7–15 meters above the base of the Jim Camp Wash beds (his “Facies E”), although in fact they are usually 7 meters or less above the Jasper Forest bed and Rainbow Forest Bed. At many localities other less well-developed silcrete horizons are present within a meter or two of the most distinctive and well-developed horizon (Figure 14c, and see Peninsula section, Figures S7a,e), and the best-developed horizons vary from place to place. Individual silcrete horizons within this interval are generally no more than 10 cm thick (Figures 14a,c), but the silcrete capping the Red Band Buttes (the “agatized conglomerate” comprising unit 13 in the “Hill 5573” of Heckert and Lucas [11]) is up to a meter thick (Figure 11f, Figure 14b, see Red Band Buttes section, Figures S8c–d). Locally along the southeastern end of the Mountain Lion Cliffs, and some distance north of Long Logs (see the North of Long Logs section, Figure S10b), the silcrete becomes a pinkish-colored coarse-grained sandstone. The fact that these sandstones are equivalent to the silcrete can be confirmed by physically tracing them a short distance along outcrop to where the resume their more typical character. The silcretes are commonly deep red, pinkish-orange, or (often when occurring in sandy facies) black on the outside, and red, orange, black, gray, or milky white on the inside. Due to their frequently dark red color, we refer to this stratigraphic interval as the “persistent red silcrete zone” (Figure S1).

Two other stratigraphic intervals which usually contain silcrete horizons are present in the Sonsela Member (Figure 5b), although the silcretes are more discontinuous than in the persistent red silcrete zone. Woody [12], [42] identified one of these, which occurs several meters below the base Jasper Forest bed in the upper Lot's Wife beds (his “Facies D”). Woody ([12]fig. 8) used these silcretes to correctly correlate the sections exposed at Blue Mesa and Agate Mesa (*contra* [11]). The outside of these silcretes is usually orange (although this color is also sometimes also seen in the persistent red silcretes), and for this reason this interval is referred to as the “persistent orange silcrete zone.” Another, even more discontinuous black silcrete horizon occurs near the base of the Martha's Butte beds, usually in reddish and tan “candy-striped” friable sands just above the level of lower Flattops One sandstones. This level is referred to as the “persistent black silcrete zone.”

The silcretes are composed of silicified plant material, although the mode of preservation varies ([42]p. 63–68). Woody [12], [42] noted that the persistent red and persistent orange silcrete horizons frequently have a dendritic pattern. One of the most dramatic expressions of this is in the persistent orange silcrete in the upper Lot's Wife beds the Billings Gap area, east of Blue Mesa (Figure 14d). Woody [42] interpreted these as representing silicified root mats, indicating a relatively high and stable water table, which encouraged plant roots to spread laterally rather than vertically. Demko [47] also interpreted his “paleosol plant-bearing units (PBUs)”, which included silicified roots and rotted wood, as having formed in poorly-drained conditions, and may have been referring (at least in part) to these horizons. The persistent red silcretes sometimes occur in a zone of intense red and gray mottling, indicating pedogenic development (in the area north of Long Logs), and/or at the top of a sharply truncated package of friable sand directly overlying the Jasper Forest bed/Rainbow Forest Bed (see East of Petroglyphs section, Figures S3g–h). It may be therefore that the silcrete horizons indicate depositional hiatuses.

Alternately, the silcretes may indicate disruptions in the biota. Red and black agatized petrified wood is also sometimes found in the silcrete horizons. Creber and Ash [76] described a stratigraphic horizon containing abundant deformed red and black petrified wood showing what they interpreted as evidence of evidence of fungal infection. This horizon was described as occurring about 8 meters below the Jasper Forest bed, and shown (Creber and Ash [76]fig. 1) occurring about an equal distance between the Jasper Forest bed and the Rainbow Forest Bed (following most previous workers, Creber and Ash considered these to be stratigraphically distinct units). However, although Creber and Ash [76] did not give detailed locality information where this horizon may be observed, Sid Ash (personal communication) has identified both the persistent orange silcrete zone at Blue Mesa and the persistent red silcrete zone above the Rainbow Forest Bed northeast of Rainbow Forest as representing this “single” horizon. Creber and Ash's [76] interpretation of these silcretes as representing a catastrophic die-off of conifers may be significant, as the persistent red silcrete zone may also mark the level of a significant turnover in the vertebrate fauna [70].

### Stratigraphic Units of the “Sinking Ship”

The “Sinking Ship” is a butte located north of Blue Mesa, in which the strata dip at an anomalously steep angle to the northeast (Figure 11h). Woody [12], [42] identified this sandstone forming the “prow” of the ship as being the Camp Butte beds. However, the lithologic characteristics of this unit, a highly siliceous conglomeratic sandstone containing bright red petrified wood, are more consistent with the unit being the Jasper Forest bed. This is weakly corroborated by the presence of a reddish silcrete a few meters above this bed, in a dark reddish mudstone more reminiscent of the lower Jim Camp Wash beds than the distinctive purple and white banded lower Lot's Wife beds exposed at Blue Mesa just to the south. Moreover, the light brown, coarse-grained, muddy, and generally non-conglomeratic, and blocky weathering sandstone capping the Sinking Ship resembles most the Flattops One sandstones (as it was correctly identified by Woody [42]). The Sinking Ship therefore represents the most northerly outcrop of the upper part of the Sonsela Member within PEFO south of the Puerco River.

Moreover, these correlations indicate that the Sinking Ship has literally “sunk” more than 30 meters, as this is the approximate difference in elevation between the prow of the ship and the Jasper Forest bed capping Blue Mesa. This subsidence may be due to the subsurface dissolution of evaporates in the Permian Supai Formation, as these deposits extensively underlie the Chinle Formation in the PEFO region [77]. Deformation of the Chinle Formation due to subsurface salt tectonism has been documented elsewhere [78].

### The Sonsela Member-Petrified Forest Member Contact

Heckert and Lucas [11] and Woody [12] placed the boundary between the Sonsela Member and the Petrified Forest Member (*sensu* [12]) at the top of the sandstone they identified as the “Agate Bridge Bed”/“Flattops One bed.” However, as already discussed this unit actually consists of stratigraphically distinct units, the upper of which (the Martha's Butte beds) contains several Flattops One sandstones occurring at slightly different stratigraphic levels. The boundary proposed by Heckert and Lucas [11] and Woody [12] therefore cannot be applied consistently.

However, a very distinct stratigraphic horizon occurs at the top of the Martha's Butte beds which can be traced throughout the study area (Figure S1). Immediately below the Flattops Two Bed is a unit of purple mudstone (Figures 12a,c,f; see Dalton Site, Martha's Butte, “Gatesy's Plunge section 2” sections, Figures S5c–d, Figures S6c,e, Figures S7f–g), usually exhibiting only faint greenish-gray mottling and showing “popcorn” weathering. This unit is informally referred to here as the “monotonous purple beds” (it is in part Herrick's “Facies E” at her “Gatesy's Plunge” locality), and is distinct from the usually dark red mudstones in the Petrified Forest Member above the Flattops 2 Bed (see Upper Flattops West and Lower Flattops West sections, Figures S5e–h). More importantly, there is usually a very abrupt and very easily identified contact between the base of the monotonous purple beds and the top of the Martha's Butte beds. The latter tend to be lighter-colored candy-striped friable sand, locally with heavily pedogenically altered “purple mottled beds” and/or resistant ledge-forming upper Flattops One sandstones at the contact (such as at Martha's Butte). The contact is usually sharp and probably unconformable, although locally it is more gradational. Nonetheless, because of its distinctness and lateral extent we place the boundary between the Sonsela and Petrified Forest Members at this contact.

In the drainages at the head of Jim Camp Wash and Starving Man Wash, which are separated by less than a kilometer (Figure S1, Figure 2), this contact is unusually indistinct and difficult to identify. In this area, the sandy Martha's Butte beds are usually muddy, mottled purple, gray and reddish, and also exhibit “popcorn” weathering. This makes them difficult to distinguish from the monotonous purple beds, although the contact may still be faintly discerned. At the head of Jim Camp Wash, the monotonous purple mudstone also locally grades laterally into, and is partly incised by, a distinctive sandy and conglomeratic unit at the base of the Flattops 2 Bed (Figure S1), which slightly incises the top of the Martha's Butte beds, and contains abundant orange concretions and white and orange “non-jasperized” petrified wood preserved by permineralization.

## Discussion

### The Importance of Walking out Contacts and Mapping

Petrified Forest National Park arguably contains the best exposed, most accessible, and most thoroughly studied terrestrial Upper Triassic deposits in the world. Nonetheless, the current study emphasizes that important misunderstandings can arise or persist regarding even such well-studied strata, if lithostratigraphic correlations are not confirmed by physically walking out contacts throughout the study area, and preferably documenting these contacts with mapping. It is significant that problems with the revised correlations of Heckert and Lucas [11] and Woody [12], [42] were first suggested by problems encountered during mapping [29]. It is also significant that Martz [32] was able to use detailed mapping and the physical tracing of persistent sandstone units within the Dockum Group of West Texas to help resolve conflicts in lithostratigraphic and biostratigraphic correlations in that region [56], [79], and that comparisons with Lehman's ([56]fig. 4) geologic map helped identify exactly how and where correlation errors occurred ([32]p. 85–93). We find it very difficult to lend credence to any lithostratigraphic models, particularly those prompting unorthodox reinterpretations of biostratigraphic patterns, which do describe in detail (and preferably show) show the geographic distribution of lithologic marker beds (e.g., [27]).

### Tr-4 Unconformity

Our work confirms Woody's [12], [42] doubts about the existence of a single unconformable surface (the Tr-4 unconformity) at the base of the Sonsela Member in PEFO, contra Heckert and Lucas [11]. The Camp Butte beds, which form the base of the expanded Sonsela Member of Heckert and Lucas [11], consists of discontinuous lenses of sandstone and conglomerate which are complexly interbedded with both the uppermost Blue Mesa Member and lowermost Lot's Wife beds. Each individual lens has an unconformable base which scours into Blue Mesa Member mudstones, but they do not fall along a single regional unconformable surface.

In contrast, the erosional bases of the Jasper Forest bed and Rainbow Forest Bed do represent more continuous surfaces, at least as far as we have been able to follow them. However, the lateral facies change of the Jasper Forest bed into more friable and locally less conglomeratic sandstone at Mountain Lion Cliffs and east of Crystal Forest, and the fact that the unit thins to only a few meters at Red Band Buttes, suggests that it may well pinch out beneath the Flattops. This does not necessarily mean that the unconformity at the base of the sandstones does not persist even if the sandstones themselves are absent (as the Tr-3 unconformity at the base of the Chinle Formation persists even though the channel deposits of the overlying Shinarump Member pinch in and out; e.g., Stewart et al. [1]). Moreover. Beer [80] noted that the unconformity at the base of the Moss Back Member in Utah, which Lucas [3], [30] identified as the Tr-4 unconformity, can be traced over long distances, with well-developed paleosols occurring on the interfluves between incised channel deposits. Nonetheless, the work of May [55] and Martz [32] in West Texas shows that the Tr-4 unconformity, even if it exists locally, does not extend throughout the western United States.

In addition, our work in the park demonstrates that the transition between characteristic elements of the Adamanian and Revueltian vertebrate faunas occurs low in the Jim Camp Wash beds, not at the base of the Jasper Forest bed and Rainbow Forest Bed [70]. Therefore, even if a regional Tr-4 unconformity exists at the base of the Jasper Forest bed and Rainbow Forest Bed, it does not appear to mark a significant faunal turnover (*contra* [3], [30], [31]).

Degradational/aggradational cycles seen in Upper Triassic strata of the western United States may be more numerous and complex than often appreciated. Many workers have postulated the existence of two or three major cycles of degradation within Upper Triassic deposits of the western United States (corresponding to the Tr-3, Tr-4 and Tr-5 unconformities of Pipiringos and O'Sullivan [53] and Lucas [30]), each followed by generally fining-upward aggradational sequences [3], [30], [79], [81], [82]. However, other degradational/aggradational episodes have been observed. A depositional hiatus accompanied by extensive paleosol development occurs above the Shinarump Member in Utah [80], and several degradational/aggradational cycles occur above the postulated “Tr-4” unconformity within both the Petrified Forest Member of Arizona [83]–[85] and the Cooper Canyon Formation of Texas [79], [86]. The lateral extent of most the unconformities marking the bases of these packages are also unclear, and at least some (such as those associated with the “Tr-4 unconformity”) may be localized.

The causes of these degradational/aggradational cycles is also unclear. Beer [80] and Dubiel and Hasiotis [87] suggested that episodes of increased incision and clastic influx were driven by increased precipitation associated with climatic changes. However, Cleveland et al. [81] noted that the “Tr-4” and “Tr-5” unconformities are not associated with evidence of increased precipitation. Lucas [3], [30] argued that these cycles instead represented shifts in base level driven by eustatic sea level change, while Kraus and Middleton [83] and Cleveland et al. [81] suggested that they were driven by tectonic uplift. Moreover, Lehman and Chatterjee [79] noted that major shifts in paleocurrent direction and sediment provenance occur around the level of the “Tr-4 unconformity” in the Dockum Group of West Texas which cannot be explained by changes in sea level or precipitation, and probably indicate tectonic reorientation of the basin. In summary, the number and significance of degradational/aggradational cycles within Upper Triassic strata of the western United States is not resolved, and they may have been caused by a complex interaction of climatic and tectonic factors which have yet to be fully understood [82].

### Late Triassic Depositional and Climatic Changes Recorded in the Chinle Formation of Petrified Forest National Park

During the Late Triassic, western North America was situated about 5°–10° north of the equator near the western margin of the Pangean supercontinent (e.g., [88]). The Chinle Formation was deposited across much of Arizona, New Mexico, Utah, and Colorado, in a back-arc basin associated with the magmatic arc extending through southwestern Arizona [89]–[91] by a variety of fluvial, lacustrine, and paludal systems [2], [45], [70], [92]. Trunk rivers originating in western Texas and/or eastern New Mexico flowed northwest to the coastline in Nevada (e.g., [1], [2], [9], [93]–[94]). Sediments (including airborne volcanic detritus) entering the Chinle depocenter were derived primarily from the volcanic arc to the southwest and/or from a northeasterly sloping upland associated with the arc (the “Mogollon Slope”), from remnants of the Ancestral Rocky Mountains to the northeast, and from uplifted Precambrian and Paleozoic rocks in Texas [1], [2], [72], [93]–[96]. Chinle deposition was punctuated by alternating periods of degradation and aggradation due to tectonic, eustatic, and/or climatic changes (e.g., [30], [79], [83], [86], [97]) which have yet to be resolved.

The presence of fossils in the Chinle Formation such as ferns, horsetails, freshwater fish, giant amphibians, and aquatic reptiles indicate perennial rivers and/or lakes [98]–[101]). However, mottled gleyed, calcic, and vertic paleosols, rhizoconcretions, pedogenic carbonate nodules, locally densely packed lungfish and crayfish burrows, and regular banding in bivalve shells, all suggest that precipitation was episodic, and possibly highly seasonal [102]. The climate during deposition of the Chinle Formation is generally accepted to have been warm but with highly seasonal precipitation (a “megamonsoonal” climate) caused by altered patterns of atmospheric circulation driven by the configuration of the Pangean supercontinent around the equator [102]–[104], although there is debate as to exactly how arid conditions may have been during the “dry season” [73], [101], [102], [105]. Sedimentological evidence (discussed below) indicates the development of an increasingly arid climate throughout the course of Chinle deposition, probably driven by the movement of western North America out of the tropics and into the drier mid-latitudes (e.g., [87], [102]).

The lower part of the Chinle Formation was deposited in paleovalleys that were incised into the Early-Middle Triassic Moenkopi Formation and older Permian strata, and formed the Tr-3 unconformity [53]. This incision occurred sometime during the late Middle Triassic or early Late Triassic, and subsequent deposition of the Chinle Formation is usually considered on the basis of biostratigraphic data to have begun during the late Carnian (e.g., [3], [61]), although based on the revised date for the Carnian-Norian boundary [58] it might not have begun until the early Norian. The discontinuous conglomeratic channel sandstones of the Shinarump Member were deposited by braided, and later meandering river systems confined within these paleovalleys [92], [106]. Fill of the paleovalleys continued in Arizona and New Mexico with deposition of the Mesa Redondo and Bluewater Creek Members [1], [107]. Although little is known about the depositional and climatic conditions under which these members formed, the well-studied Monitor Butte Member of Utah may be at least partially syndepositional [1], [3], and formed in a variety of fluvial, paludal, and lacustrine environments [80], [87], [102], [108]. For reasons that we will discuss in a future paper, we agree with Parker [7] that the uppermost Mesa Redondo Member forms the reddish beds exposed at the base of the Blue Mesa Member in PEFO (*contra* [14], [39], [40]), and we disagree with Demko's [47], [48] referral of the lower part of the overlying Blue Mesa Member to the Monitor Butte Member.

The Blue Mesa Member, the lowest unit with extensive exposure in PEFO, began to be deposited about 219 Ma, well into the Norian according to the recently revised Late Triassic timescale [58], [67]. The Blue Mesa Member was deposited by a mixed-load meandering river system, of which the Newspaper Rock Bed represents channel deposits [12], [47], [83]. The Blue Mesa Member is dominated by overbank deposits, which are drab-colored mudstones containing abundant gleyed paleosols, indicating the presence of highly seasonal precipitation, abundant organic material, rapid sedimentation, extended saturation of soils, and possibly at least seasonal wetland conditions [12], [47], [48], [108]–[110]. This interpretation is supported by the abundance of large temnospondyl amphibians in the Blue Mesa Member [24], [111], as well as fossil ferns similar to those inhabiting the modern day humid tropical and subtropical environments [101]. Although Simms et al. [112] cited a variety of evidence indicating that conditions were wetter globally during the Carnian than in the Norian (their “Carnian pluvial episode”), the depositional and climatic conditions indicated by both the Monitor Butte Member and Blue Mesa Member suggest that wet conditions persisted into the early Norian in western North America.

The onset of deposition of the Sonsela Member during the (middle or late?) Norian indicates a fairly dramatic shift in the depositional regime. Overlying and interfingering with the thick overbank mudstones of the uppermost Blue Mesa Member, the Camp Butte beds consist of a multiple discontinuous conglomeratic sandstone lenses deposited by invading bedload-dominated braided rivers bringing in abundant extrabasinal sediments [12], [52]. Herrick [52] interpreted the overlying horizontally-bedded deposits of alternating purple mudstone and white sand in the lower Lot's Wife beds as well-drained overbank mudstones punctuated by crevasse splays associated with the bedload-dominated streams that deposited the Camp Butte beds. As already discussed, the cause of this shift in depositional regime is unclear, although it does not appear to have been proceeded by an extended depositional hiatus (the “Tr-4 unconformity” of Lucas [3], [30]).

The upper Lot's Wife beds represent the return of sandy and frequently conglomeratic channel deposits and muddier channel fills (Herrick [52]; Woody's [12] “Facies D”) likely representing initial deposition of the Jasper Forest bed/Rainbow Forest Bed river system. The Jasper Forest bed and Rainbow Forest Bed were deposited by low sinuosity, bedload-dominated braided rivers exhibiting high energy but possibly ephemeral flow [45], [52], [71], [83]. However, Espregen [71] and Woody [42] both suggested that high mudstone content in these sandstones indicates they may have been deposited, at least in part, in high-energy mixed-load and moderate-sinuosity channels. Clasts in the Jasper Forest bed and Rainbow Forest Bed are dominated by extrabasinal chert and quartzite [1], as well as volcanic clasts of Triassic age [72]. The presence of an at least localized depositional hiatus (the “Tr-4 unconformity”) prior to the incision of these channel systems is possible but ambiguous.

The upper Sonsela Member and Petrified Forest Member (*sensu* [12]; Painted Desert Member of the Petrified Forest Formation *sensu* [11] and upper Petrified Forest Member of most previous workers) show evidence of having been deposited by both bedload-dominated low-sinuosity rivers and mixed-load high-sinuosity rivers, with the latter becoming predominant. Larger channel sands in the Jim Camp Wash beds were deposited by vertical and lateral accretion in meandering channels, although ribbon sandstones representing smaller low-sinuosity channels are also present (Herrick [52]; Woody's [12] “Facies E”). Espregen [71] interpreted the Flattops One sandstones in the Martha's Butte beds as having been deposited in bedload-dominated low-sinuosity rivers. However, Woody [12], [42] noted a more sinuous channel system was suggested for some of the Flattops One sandstones (his “G Facies”) by the presence of lateral accretion bedding and “ridge and swale” scroll bar topography. Although he suggested that channels became more sinuous higher in the section, the distinctive scroll bars visible from the main park road just south of the Flattops (see Woody [12]fig. 6) actually occur fairly low in the Martha's Butte beds, below the “candy-striped beds.”

Although Herrick [52] indicated that there was no significant difference between the Lot's Wife beds and Jim Camp Wash beds, well-developed mottled paleosol horizons and pedogenic carbonate nodules (and consequently, channel gravels composed of reworked carbonate nodules) are locally far more abundant and better developed in both the Jim Camp Wash beds and Martha's Butte beds than seen in either the Blue Mesa Member or Lot's Wife beds. These differences suggest that a shift from poorly-drained wetlands to well-drained drained soils and possibly a more arid climate occurred during deposition of the Sonsela Member, which is supported by Espregen's [71] identification of a possible playa lake deposit in the Martha's Butte beds. However, lower sedimentation rates encourage paleosol development (e.g., [113]), so it is conceivable that the higher pedogenic development of the Jim Camp Wash beds indicates slower sedimentation rather than better-drained soils and a more arid climate. Improved calibration of sedimentation rates through improved radioisotopic dating of the Sonsela Member may help resolve this question. The cause of this sedimentological change is of particular interest, as it may coincide with faunal and floral reorganizations [70].

One interesting possible side effect of this increase in carbonate nodule development is the great abundance of unionid bivalves in the upper Sonsela Member and Petrified Forest Member, which are virtually unknown in the Blue Mesa Member and lower Sonsela Member [114]. Unionids prefer relatively alkaline waters, and today can be extremely abundant in streams with high levels of dissolved calcium and carbon dioxide, which are essential for shell development [115]. Therefore, the spectacular beds of unionid bivalves in the Jim Camp Wash beds, Martha's Butte beds, and Petrified Forest Member, might be tied to the increase in reworked carbonate nodules present in stream gravels.

The Petrified Forest Member is predominantly a mixed-load, meandering river system dominated by overbank mudstones, although non-sinuous bedload-dominated streams persisted (e.g., [47], [70], [83], [84]), and infilled scours within overbank mudstones show that there were repeated degradational/aggradational cycles during deposition of the Petrified Forest Member [83]–[85]. The Petrified Forest Member consists mostly of red and purple mudstones with abundant vertic paleosols and pedogenic carbonate nodules, indicating that the relatively well-drained overbank deposits and possibly increasingly arid climatic conditions which began during deposition of the Sonsela Member persisted [12], [83], [87], [71], [109], [110]. This is supported by the relative rarity of large temnospondyl amphibians ([24], [111], [70]) in both the upper Sonsela and Petrified Forest Members.

The overlying Owl Rock and Rock Point Members in northeastern Arizona (as well as the Church Rock Member of southern Utah, which is equivalent to the Rock Point Member), contain well-developed pedogenic carbonate horizons, including not only nodules but calcretes, as well as eolian deposits, suggesting the development of increasingly arid conditions in the region during the Late Triassic [1], [87], [92], [102], [110], [116]. The Black Forest Bed near the top of the Petrified Forest Member has been dated at 211–213 Ma [68]–[69], close to the Norian-Rhaetian boundary age of 207–210 Ma [59], suggesting that deposition of the Owl Rock and Rock Point Members occurred during the Rhaetian. The climatic trends of the upper Chinle Formation cumulated with the formation of the massive eolian dune fields of the Glen Canyon Group during Early-Middle Jurassic time (e.g., [117]).

## Supporting Information

Appendix S1 Description of measured sections.
(0.17 MB DOC)

Figure S1 Geologic map of the Chinle Formation (Upper Triassic) in the region of Petrified Forest National Park between Jasper Forest and the Flattops. The location of the map is shown by the smaller park map on the upper left, and unit symbols are explained by the stratigraphic column and key on the lower left. Dashed contact lines indicate where a contact is either arbitrary due to being gradational, or poorly exposed. The contacts for Quaternary deposits, which are often thin layers of wind-blown sand patchily concealing Chinle Formation outcrops, are particularly arbitrary, and should be taken with a grain of salt.
(9.47 MB TIF)

Figure S2 Key to symbols used in measured sections.
(0.18 MB TIF)

Figure S3 Labeled photographs and diagrams of measured sections 1–4. South End Knob at 12S E0602076 N3851723 NAD 27, photo (a) and section (b); South End Cliff at 12S E0601939 N3851827 NAD 27, photo (c) and section (d); “PFNP-14”/“Giant Logs section” of Roadifer [17] and Heckert and Lucas [11] at 12S E0602800 N3854095 NAD 27, photo (e) and section (e–f); East of Petroglyphs at 12S E0604707 N3854159 NAD 27 photo (g) and section (g–h).
(7.55 MB TIF)

Figure S4 Labeled photographs and diagrams of measured sections 5–8. Bowman 2/Bowman South at 12S E0604866 N3854341 NAD 27, photo (a) and section (b); Bowman 3 at 12S E0604793 N3854410 NAD 27, photo (c) and section (d); Bowman 1 at 12S E0604831 N3854555 NAD 27, photo (e) and section (f); No Name Point 3 of Woody [42] at 12S E0603673 N3854544 NAD 27 photo (g) and section (h).
(7.90 MB TIF)

Figure S5 Labeled photographs and diagrams of measured sections 9–12. No Name Point 2b section of Woody [42] at 12S E0606203 N3854676 NAD 27 photo (a) and section (b); Dalton Site at 12S E0606877 N3855141 NAD 27, photo (c) and section (d); Lower “Flattops West” of Heckert and Lucas [11] photo at 12S E0607645 N3854991 NAD 27 (e) and section (f); Upper “Flattops West” section of Heckert and Lucas [11] at 12S E0607767 N3855109 NAD 27 photo (g) and section (h).
(10.26 MB TIF)

Figure S6 Labeled photographs and diagrams of measured sections 13–15. Dry Wash Bridge East at 12S E0608669 N3856310 NAD 27, photo (a) and section (b); Walker's Stump and Martha's Butte at 12S E0608292 N3856717 NAD 27, photograph (c); Walker's Stump section (d); Martha's Butte section (e).
(7.75 MB TIF)

Figure S7 Labeled photographs and diagrams of measured sections 16–18. Photographs of micro-sections used to create composite section for the Peninsula at 12S E0608872 N3857800 NAD 27 (a), 12S E0608701 N3857648 NAD 27 (b), 12S E0608644 N3857522 NAD 27 (c), 12S E0608489 N3857317 NAD 27 (d), composite Peninsula section (e); “Gatesy's Plunge section 2” of Herrick [52] at 12S E0607320 N3858600 NAD 27, photo (f) and section (g), “Gatesy's Plunge section 4” of Herrick [52] at 12S E0607655 N3858302 NAD 27, photo (h) and section (i).
(6.59 MB TIF)

Figure S8 Labeled photographs and diagrams of measured sections 19–22. Mountain Lion Cliffs at 12S E0608065 N3858693 NAD 27, photo (a) and section (b), Photograph of more northerly Red Band Butte and Near Battleship Quarry photographed at about 12S E0609097 N3859146 NAD 27 (c), Red Band Butte section (d), Near Battleship Quarry section (e), Flag Canyon at 12S E0611631 N3859786 NAD 27, photo (f) and section (g).
(9.01 MB TIF)

Figure S9 Labeled photographs and diagrams of measured sections 23–25. “Lot's Wife section 3”/“PFNP-5” sections of Herrick [52] and Roadifer [17] at 12S E0609915 N3862732 NAD 27, photo (a) and section (b); Tepees to Camp's Butte, photo with foreground at 12S E0612452 N3867253 NAD 27 (c) and section (d); Blue Mesa Pronghorn Trail at 12S E0614297 N3866933 NAD 27, photo (e) and section (f).
(7.92 MB TIF)

Figure S10 Labeled photographs and diagrams of measured sections 26–29. North of Long Logs at 12S E0605581 N3852976 NAD 27, photo (a) and section (b); Near Little Battleship, photo of lower part of section at 12S E0606518 N3853673 NAD 27 (c), photo of upper part of section at 12S E0606462 N3853772 NAD 27 (d), section (e); Stemwedel Site section at 12S E0607365 N3853104 NAD 27, photo (f) and section (g); Near Milkshake Quarry at 12S E0605069 N3850861 NAD 27, photo (h) and section (i).
(6.81 MB TIF)

## References

[1] Stewart JH, Poole FG, Wilson RF (1972a). Stratigraphy and origin of the Chinle Formation and related Upper Triassic strata in the Colorado Plateau region.. United States Geological Survey Professional Paper.

[2] Dubiel RF, Caputo MV, Peterson JA, Franczyk KJ (1994). Triassic deposystems, paleogeography, and paleoclimate of the Western Interior.. Mesozoic Systems of the Rocky Mountain Region, USA.

[3] Lucas SG, Morales M (1993). The Chinle Group: revised stratigraphy and biochronology of Upper Triassic nonmarine strata in the western United States.. Aspects of Mesozoic Geology and Paleontology of the Colorado Plateau: Museum of Northern Arizona Bulletin.

[4] Long RA, Murry PA (1995). Late Triassic (Carnian and Norian) tetrapods from the southwestern United States: New Mexico Museum of Natural History and Science Bulletin.

[5] Camp CL (1930). A study of the phytosaurs, with description of new material from western North America. Memoirs of the University of California.

[6] Stagner HR (1941). Geology of the fossil leaf beds of the Petrified Forest National Monument. In: Daugherty LH. The Upper Triassic Flora of Arizona.. Carnegie Institution of Washington Publication.

[7] Parker WG, Parker WG, Ash SR, Irmis RB (2006). The stratigraphic distribution of major fossil localities in Petrified Forest National Park, Arizona.. A Century of Research at Petrified Forest National Park 1906-2006: Museum of Northern Arizona Bulletin.

[8] Stocker MR (2008). The relationships of the phytosaur *Leptosuchus* Cape 1922 with descriptions of new material from Petrified Forest National Park, Arizona (unpublished Master's thesis)..

[9] Axsmith BJ (2009). A new *Cynepteris* from the Upper Triassic of Arizona: potential implications for the early diversification of schizaelean ferns.. International Journal of Plant Science.

[10] Repenning CA, Cooley ME, Akers JP (1969). Stratigraphy of the Chinle and Moenkopi Formations, Navajo and Hopi Indian Reservations Arizona, New Mexico, and Utah.. United States Geological Survey Professional Paper 521-B.

[11] Heckert AB, Lucas SG, Heckert AB, Lucas SG (2002). Revised Upper Triassic stratigraphy of the Petrified Forest National Park, Arizona, U.S.A.. Upper Triassic Stratigraphy and Paleontology: New Mexico Museum of Natural History and Science Bulletin.

[12] Woody DT, Parker WG, Ash SR, Irmis RB (2006). Revised stratigraphy of the Lower Chinle Formation (Upper Triassic) of Petrified Forest National Park, Arizona.. A Century of Research at Petrified Forest National Park 1906-2006: Museum of Northern Arizona Bulletin.

[13] Akers JP, Cooley ME, Repenning CA, Anderson RY, Harshbarger JW (1958). Moenkopi and Chinle Formations of Black Mesa and adjacent areas.. New Mexico Geological Society Guidebook.

[14] Dubiel RF, Hasiotis ST, Demko TM, Santucci VL, McClelland L (1999). Incised valley fills in the lower part of the Chinle Formation, Petrified Forest National Park, Arizona: complete measured sections and regional stratigraphic implications of Upper Triassic rocks.. National Park Service Paleontological Research: Technical Report NPS/NRPO/GRTR-99/3.

[15] Dubiel RF, Lucas SG, Morales M (1993). Depositional setting of the Owl Rock Member of the Upper Triassic Chinle Formation, Petrified Forest National Park and vicinity, Arizona.. The Nonmarine Triassic: New Mexico Museum of Natural History and Science Bulletin.

[16] Cooley ME (1957). Geology of the Chinle Formation in the upper Little Colorado drainage area, Arizona and New Mexico (unpublished Master's thesis)..

[17] Roadifer JE (1966). Stratigraphy of the Petrified Forest National Park, Arizona (unpublished Ph.D dissertation)..

[18] Billingsley GH (1985a). General stratigraphy of the Petrified Forest National Park, Arizona.. Museum of Northern Arizona Bulletin.

[19] Ash SR (1987). Petrified Forest National Park, Arizona.. Geological Society of America Centennial Field Guide-Rocky Mountain Section.

[20] Murry PA (1990). Stratigraphy of the Upper Triassic Petrified Forest Member (Chinle Formation) in Petrified Forest National Park, Arizona, USA.. Journal of Geology.

[21] Dubiel RF, Hasiotis ST, Demko TM, Riggs NR, May CL, Santucci VL (1994). A composite measured section, Upper Triassic Chinle Formation, Petrified Forest National Park, Arizona.. Petrified Forest National Park Research Abstracts, Petrified Forest National Park.

[22] Lucas SG, Santucci VL, McClelland L (1995). Revised Upper Triassic stratigraphy, Petrified Forest National Park.. National Park Service Paleontological Research: Technical Report NPS/NRPO/GRTR-95/16.

[23] Long RA, Ballew KL, Colbert EH, Johnson RR (1985). Aetosaur dermal armor from the Late Triassic of southwestern North America, with special reference to material from the Chinle Formation of Petrified Forest National Park.. The Petrified Forest through the ages: Museum of Northern Arizona Bulletin.

[24] Long RA, Padian K, Padian K (1986). Vertebrate biostratigraphy of the Late Triassic Chinle Formation, Petrified Forest National Park, Arizona: preliminary results.. The Beginning of the Age of Dinosaurs: faunal change across the Triassic-Jurassic boundary.

[25] Hunt AP, Lucas SG, Santucci VL, McClelland L (1995). Two Late Triassic vertebrate faunas at Petrified Forest National Park.. National Park Service Paleontological Research: Technical Report NPS/NRPO/NRTR-95/16.

[26] Woody DT, Parker WG (2004). Evidence for a transitional fauna within the Sonsela Member of the Chinle Formation, Petrified Forest National Park, Arizona.. Journal of Vertebrate Paleontology.

[27] Hunt AP, Lucas SG, Heckert AB, Lucas SG, Zeigler KE, Lueth VW, Owen DE (2005). Definition and correlation of the Lamyan: a new biochronological unit for the nonmarine Late Carnian (Late Triassic).. Geology of the Chama Basin: New Mexico Geological Society Guidebook, 56^th^ Field Conference.

[28] Parker WG, McCord RD (2005). Faunal review of the Upper Triassic Chinle Formation of Arizona.. Vertebrate Paleontology of Arizona, Mesa Southwest Museum Bulletin.

[29] Raucci JJ, Blakey RC, Umhoefer PJ, Parker WG, Ash SR, Irmis RB (2006). A new geologic map of Petrified Forest National Park with emphasis on members and key beds of the Chinle Formation.. A Century of Research at Petrified Forest National Park 1906-2006: Museum of Northern Arizona Bulletin.

[30] Lucas SG (1991). Sequence stratigraphic correlation of nonmarine and marine Late Triassic biochronologies, western United States.. Albertiana.

[31] Heckert AB, Lucas SG (1996). Stratigraphic description of the Tr-4 unconformity in west-central New Mexico and eastern Arizona.. New Mexico Geology.

[32] Martz JW (2008). Lithostratigraphy, chemostratigraphy, and vertebrate biostratigraphy of the Dockum Group (Upper Triassic), of southern Garza County, West Texas. (unpublished PhD dissertation)..

[33] Stewart JH, Poole FG, Wilson RF, Breed CS, Breed WJ (1972b). Changes in nomenclature of the Chinle Formation on the southern part of the Colorado Plateau: 1850s-1950s.. Investigations in the Chinle Formation: Museum of Northern Arizona Bulletin.

[34] Gregory HE (1917). Geology of Navajo County: United States Geological Survey Professional Paper 93..

[35] Stewart JH (1957). Proposed nomenclature of part of Upper Triassic strata in southeastern Utah.. American Association of Petroleum Geologists Bulletin.

[36] Harshbarger JW, Repenning CA, Irwin JA (1957). Stratigraphy of the uppermost Triassic and the Jurassic rocks of the Navajo Country: United States Geological Survey Professional Paper 291..

[37] Kiersch GA (1956). Metalliferous minerals and mineral fuels, geology, evaluation, and uses, with a section on general geology. Mineral resources Navajo-Hopi Reservations, Arizona Utah, vol. 1..

[38] Cooley ME (1958). The Mesa Redondo Member of the Chinle Formation, Apache and Navajo Counties, Arizona.. Plateau.

[39] Heckert AB, Lucas SG, Santucci VL, McClelland L (1998b). The oldest Triassic strata exposed in Petrified Forest National Park, Arizona.. National Park Service Paleontological Research: Technical Report NPS/NRGRD/GRDTR 98/01.

[40] Therrien F, Jones MM, Fastovsky DE, Herrick AS, Hoke GD, Santucci VL, McClelland L (1999). The oldest Triassic strata exposed in Petrified Forest National park revisited.. National Park Service Paleontological Research: Technical Report NPS/NRPO/GRTR-99/3.

[41] Gregory HE (1950). Geology and geography of the Zion Park Region Utah and Arizona: United States Geological Society Professional Paper 220..

[42] Woody DT (2003). Geologic reassessment of the Sonsela Member of the Chinle Formation, Petrified Forest National Park, Arizona (unpublished Master's thesis)..

[43] Reeside JB, Applin PL, Colbert EH, Gregory JT, Hadley HD (1957). Correlation of the Triassic formations of North America exclusive of Canada.. Bulletin of the Geological Society of America.

[44] Goebel LA (1936). A correlation of the forests in Petrified Forest National Park (unpublished National Park Service Report)..

[45] Deacon MW (1990). Depositional analysis of the Sonsela sandstone bed, Chinle Formation, northeast Arizona and northwest New Mexico (unpublished Master's thesis)..

[46] Heckert AB, Lucas SG, Santucci VL, McClelland L (1998a). Stratigraphic distribution and age of petrified wood in Petrified Forest National Park, Arizona.. National Park Service Paleontological Research: Technical Report NPS/NRGRD/GRDTR 98/01.

[47] Demko TM (1995a). Taphonomy of fossil plants in the Upper Triassic Chinle Formation (unpublished PhD dissertation)..

[48] Demko TM, Boaz D (1995b). Taphonomy of fossil plants in Petrifed Forest National Park, Arizona.. Fossils of Arizona, Proceedings of the Mesa Southwest Paleontological Society and Mesa Southwest Museum.

[49] Billingsley GH (1985b). Geologic map of Petrified Forest National Park, Arizona. Report to the Petrified Forest Museum Association, unpublished..

[50] Heckert AB (1997). Litho- and biostratigraphy of the lower Chinle Group, East-Central Arizona and West-Central New Mexico, with a description of a new theropod (Dinosauria: Theropoda) from the Bluewater Creek Formation (unpublished Master's thesis)..

[51] Lucas SG, Heckert AB, Spielmann JA, Tanner LH, Hunt AP, Lucas SG, Spielmann JA (2007). Third Day: Triassic stratigraphy and paleontology in northeastern Arizona.. Triassic of the American West: New Mexico Museum of Natural History and Science Bulletin.

[52] Herrick AS (1999). Telling time in the Triassic: biochronology and stratigraphy of the Chinle Formation in Petrified Forest National Park, Arizona (unpublished Master's thesis)..

[53] Pipiringos GN, O'Sullivan RB (1978). Principal unconformities in Triassic and Jurassic rocks, Western Interior United States - a preliminary survey. United States Professional Paper 1035-A..

[54] Lucas SG, Hunt AP, Lucas SG, Morales M (1993). Tetrapod biochronology of the Chinle Group (Upper Triassic), western United States.. The Nonmarine Triassic: New Mexico Museum of Natural History and Science Bulletin.

[55] May BA (1988). Depositional environments, sedimentology, and stratigraphy of the Dockum Group (Triassic) in the Texas Panhandle (unpublished Master's thesis)..

[56] Lehman TM (1994). The saga of the Dockum Group and the case of the Texas/New Mexico boundary fault.. New Mexico Bureau of Mines and Mineral Resources Bulletin.

[57] Ogg JG, Gradstein F, Ogg J, Smith A (2004). The Triassic Period.. A Geologic Time Scale 2004.

[58] Furin S, Preto N, Rigo M, Roghi G, Gianolla P (2006). High precision U-Pb zircon age from the Triassic of Italy: Implications for the Triassic time scale and the Carnian origin of calcareous nannoplankton and dinosaurs.. Geology.

[59] Muttoni, G, Kent DV, Jadoul F, Olsen PE, Rigo M (2010). Rhaetian magneto-biostratigraphy from the Southern Alps (Italy): Constraints on Triassic chronology.. Palaeogeography, Palaeoclimatology, and Paleoecology.

[60] Dunay RE, Fisher MJ (1974). Late Triassic palynofloras of North America and their European correlatives.. Review of Palaeobotany and Palynology.

[61] Litwin RJ, Traverse A, Ash SR (1991). Preliminary palynological zonation of the Chinle Formation, southwestern U.S.A., and its correlation to the Newark Supergroup (eastern U.S.A.).. Review of Palaeobotany and Palynology.

[62] Cornet B, Lucas SG, Morales M (1993). Applications and limitations of palynology in age, climatic, and paleoenvironmental analyzes of Triassic sequences in North America.. The Nonmarine Triassic: New Mexico Museum of Natural History and Science Bulletin.

[63] Lucas SG (1998). Global Triassic tetrapod biostratigraphy and biochronology.. Palaeogeography, Palaeoclimatology, Palaeoecology.

[64] Lucas SG, Heckert AB (2000). Biochronological significance of Triassic nonmarine tetrapod records from marine strata.. Albertiana.

[65] Channell JET, Kozur HW, Sievers T, Mock R, Aubrecht R (2003). Carnian-Norian biomagnetostratigraphy at Silická Brezová (Slovakia): correlation to other Tethyan sections and to the Newark Basin.. Palaeogeography, Palaeoclimatology, Palaeoecology.

[66] Muttoni G, Kent DV, Olsen PE, Di Stefano P, Lowrie W (2004). Tethyan magnetostratigraphy from Pizzo Mondello (Sicily) and correlation to the Late Triassic Newark astrochronological polarity time scale.. GSA Bulletin.

[67] Irmis R, Mundil R (2008). New age constraints from the Chinle Formation revise global comparisons of Late Triassic vertebrate assemblages.. Journal of Vertebrate Paleontology.

[68] Riggs NR, Ash SR, Barth AP, Gehrels GE, Wooden JL (2003). Isotopic age of the Black Forest Bed, Petrified Forest Member, Chinle Formation, Arizona: An example of dating a continental sandstone.. GSA Bulletin.

[69] Heckert AB, Lucas SG, Dickinson WR, Mortensen JK (2009). New ID-TIMS U-PB ages for Chinle Group strata (Upper Triassic) in New Mexico and Arizona, correlation to the Newark Supergroup, and implications for the “long Norian.”. Geological Society of America Abstracts with Programs.

[70] Parker W, Martz J (2009). Constraining the stratigraphic position of the Late Triassic (Norian) Adamanian-Revueltian faunal transition in the Chinle Formation of Petrified Forest National Park, Arizona.. Journal of Vertebrate Paleontology.

[71] Espregen WA (1985). Sedimentology and petrology of the Upper Petrified Forest Member of the Chinle Formation, Petrified Forest National Park and vicinity, Arizona (unpublished Master's thesis)..

[72] Riggs NR, Barth AP, González-León C, Walker JD, Wooden JL (2009). Provenance of Upper Triassic strata in southwestern North America as suggested by isotopic analysis and chemistry of zircon crystals.. Geological Society of America Abstracts with Programs.

[73] Ash SR, Nesbitt SJ, Parker WG, Irmis RB (2005). Synopsis of the Upper Triassic flora of Petrified Forest National Park and vicinity.. Guidebook to the Triassic Formations of the Colorado Plateau in northern Arizona: Geology, Paleontology, and History, Mesa Southwest Museum, Bulletin No. 9.

[74] Murry PA, Long RA, Lucas SG, Hunt AP (1989). Geology and paleontology of the Chinle Formation, Petrified Forest National Park and vicinity, Arizona, and a discussion of vertebrate fossils of the southwestern Upper Triassic.. Dawn of the Age of Dinosaurs in the American Southwest.

[75] Parker WG, Irmis RB, Parker WG, Ash SR, Irmis RB (2006). A new species of the Late Triassic phytosaur *Pseudopalatus* (Archosauria: Pseudosuchia) from Petrified Forest National Park, Arizona.. A Century of Research at Petrified Forest National Park 1906-2006, Museum of Northern Bulletin.

[76] Creber GT, Ash SR (1990). Evidence of widespread fungal attack on Upper Triassic trees in the southwestern U.S.A.. Review of Palaeobotany and Palynology.

[77] Neal JT, Rauzi SL (1996). Storage opportunities in Arizona bedded evaporates.. Solution Mining Research Institute, Meeting Paper (Fall, 1996).

[78] Hazel JE (1994). Sedimentary response to intrabasinal salt tectonism in the Upper Triassic Chinle Formation, Paradox Basin, Utah: U.S.. Geological Survey Bulletin 2000-F.

[79] Lehman T, Chatterjee S (2005). The depositional setting and vertebrate biostratigraphy of the Triassic Dockum Group of Texas.. Indian Journal of Earth System Sciences.

[80] Beer JJ (2005). Sequence stratigraphy of fluvial and lacustrine deposits in the lower part of the Chinle Formation, south central Utah, United States: paleoclimatic and tectonic implications (unpublished master's thesis)..

[81] Cleveland DM, Atchley SC, Nordt LC (2007). Continental sequence stratigraphy of the Upper Triassic (Nrian-Rhaetian) Chinle strata, northern New Mexico, U.S.A.: Allocyclic and autocyclic origins of paleosol-bearing alluvial successions.. Journal of Sedimentary Research.

[82] Blakey RC, Miall AD (2008). Pennsylvanian-Jurassic sedimentary basins of the Colorado Plateau and southern Rocky Mountains.. Sedimentary Basins of the World, Vol. 5: The Sedimentary Basins of the United States and Canada.

[83] Kraus KJ, Middleton LT (1987). Dissected paleotopography and base-level changes in a Triassic fluvial sequence.. Geology.

[84] Johns ME (1988). Architectural element analysis and depositional history of the Upper Petrified Forest Member of the Chinle Formation, Petrified Forest National Park, Arizona (unpublished master's thesis)..

[85] Love SE (1993). Floodplain deposits as indicators of sandbody geometry and reservoir architecture, Chapter Three: the Chinle Formation (unpublished Ph.D dissertation)..

[86] Frehlier AP (1986). Sedimentology, fluvial paleohydrology, and paleogeomorphology of the Dockum Formation (Triassic), West Texas (unpublished Master's thesis)..

[87] Dubiel RF, Hasiotis ST (in press) Facies complexity and climatic variability in a continental-scale system: the Upper Triassic Chinle Formation, Colorado Plateau, USA.. SEPM Special Publication on Fluvial Systems.

[88] Bazard DR, Butler RF (1991). Paleomagentism of the Chinle and Kayenta Formations, New Mexico and Arizona.. Journal of Geophysical Research.

[89] Dickinson WR (1981). Plate tectonic evolution of the southern Cordillera.. Arizona Geological Society Digest.

[90] Busby-Spera CJ (1988). Speculative tectonic model for the early Mesozoic arc of the southwest Cordilleran United States.. Geology.

[91] Lawton TF, Caputo MV, Peterson JA, Franczyk KJ (1994). Tectonic setting of Mesozoic sedimentary basins, Rocky Mountain region, United States.. Mesozoic Systems of the Rocky Mountain System, U.S.A..

[92] Blakey RC, Gubitosa R, Reynalds MW, Dolley ED (1983). Late Triassic paleogeography and depositional history of the Chinle Formation, southern Utah and northern Arizona.. Mesozoic Paleogeography of West-Central United States. Rocky Mountain Section, Society of Economic Paleontologists and Mineralogists.

[93] Gehrels GE, Dickinson WR (1995). Detrital zircon provenance of Cambrian to Triassic miogeoclinal and eugeoclinal strata in Nevada.. American Journal of Science.

[94] Riggs NR, Lehman TM, Gehrels GE, Dickinson WR (1996). Detrital zircon link between headwaters and terminus of the Upper Triassic Chinle-Dockum paleoriver system.. Science.

[95] Stewart JH, Anderson TH, Haxal GB, Silver LT, Wright JE (1986). Late Triassic paleogeography of the southern Cordillera: the problem of a source for voluminous volcanic detritus in the Chinle Formation of the Colorado Plateau region.. Geology.

[96] Bilodeau WL (1986). The Mesozoic Mogollon Highlands, Arizona: An Early Cretaceous rift shoulder.. Journal of Geology.

[97] Lupe R, Silberling NJ, Howell DG (1985). Genetic relationship between Lower Mesozoic continental strata of the Colorado Plateau and marine strata of the western Great Basin: Significance for accretionary history of Cordilleran lithotectonic terranes.. Tectonostratigraphic terranes of the Circum-Pacific region: Houston, Texas, Circum-Pacific Council for Energy and Mineral Resources, Earth Science Series, no. 1.

[98] Murry PA, Lucas SG, Hunt AP (1989). Paleoecology and vertebrate faunal relationships of the Upper Triassic Dockum and Chinle Formations, southwestern United States.. Dawn of the Age of Dinosaurs in the American Southwest.

[99] Parrish JM (1989). Vertebrate paleoecology of the Chinle Formation (Late Triassic) of the southwestern United States.. Paleogeography, Palaeoclimatology, Palaeoecology.

[100] Ash SR, Creber GT (1992). Paleoclimatic interpretation of the wood structures of trees in the Chinle Formation (Upper Triassic), Petrified Forest National Park, Arizona, USA.. Palaegeography, Palaeoclimatology, Palaeoecology.

[101] Ash SR, Santucci VL, McClelland L (2001). The fossil ferns of Petrified Forest National Park, and their paleoclimatological interpretations.. Proceedings of the 6^th^ Fossil Research Conference, National Park Service.

[102] Dubiel RF, Parrish JT, Parrish JM, Good SC (1991). The Pangean Megamonsoon-Evidence from the Upper Triassic Chinle Formation, Colorado Plateau.. Palaios.

[103] Parrish JT, Peterson F (1988). Wind directions predicted from global circulation models and wind directions determined from eolian sandstones of the western United States—A comparison.. Sedimentary Geology.

[104] Kutzbach JE, Gallimore RG (1989). Pangaean climates: Megamonsoons of the megacontinent: Journal of Geophysical Research.

[105] Demko TM, Dubiel RF, Parrish JT (1998). Plant taphonomy in incised valleys: Implications for interpreting paleoclimate from fossil plants.. Geology.

[106] Blakey RC, Gubitosa R (1984). Controls of sandstone body geometry and architecture in the Chinle Formation (Upper Triassic), Colorado Plateau.. Sedimentary Geology.

[107] Lucas SG, Hayden SN (1989). Triassic stratigraphy of West-Central New Mexico.. New Mexico Geological Society Guidebook, 40^th^ Field Conference, Southern Colorado Plateau.

[108] Dubiel RF (1987). Sedimentology of the Upper Triassic Chinle Formation, southeastern Utah: paleoclimatic implications.. Journal of the Arizona-Nevada Academy of Science.

[109] Therrien F, Fastovsky D (2000). Paleoenvironments of early theropods, Chinle Formation (Late Triassic), Petrified Forest National Park, Arizona.. Palaios.

[110] Tanner LH, Lucas SG, Semken SC, Berglof W, Ulmer-Scholle D (2003). Pedogenic features of the Chinle Group, Four Corners region: evidence of Late Triassic aridification.. Geology of the Zuni Plateau: New Mexico Geological Society Guidebook, 54^th^ field conference.

[111] Hunt AP, Lucas SG (1993). Taxonomy and stratigraphic distribution of Late Triassic metoposaurid amphibians from Petrified Forest National Park, Arizona.. Journal of the Arizona Nevada Academy of Science.

[112] Simms MJ, Ruffell AH, Johnson ALA, Fraser NC, Sues H-D (1994). Biotic and climatic changes in the Carnian (Triassic) of Europe and adjacent areas.. In the Shadow of the Dinosaurs: Early Mesozoic Tetrapods.

[113] Bown TM, Kraus MJ (1987). Integration of channel and floodplain suites, I. Developmental sequence and lateral relations of alluvial paleosols.. Journal of Sedimentary Geology.

[114] Good SC, Johnston PA, Haggart JW (1998). Freshwater bivalve fauna of the Late Triassic (Carnian-Norian) Chinle, Dockum, and Dolores Formations of the Southwest United States.. Bivalves: An Eon of Evolution-Paleobiological Studies Honoring Norman D. Newell.

[115] Smith DG (2001). Pennak's freshwater invertebrates of the United States: Porifera to Crustacea, 4^th^ Edition..

[116] Dubiel RF, Lucas SG, Hunt AP (1989). Depositional and climatic setting of the Upper Triasic Chinle Formation, Colorado Plateau.. Dawn of the Age of Dinosaurs in the American Southwest.

[117] Blakey RC, Peterson F, Kocurek G (1988). Synthesis of late Paleozoic and Mesozoic eolian deposits of the Western Interior of the United States.. Sedimentary Geology.

