# Supplementary material for: Revised Lithostratigraphy of the Sonsela Member (Chinle Formation, Upper Triassic) in the Southern Part of Petrified Forest National Park, Arizona
Source: PLoS One. 2010 Feb 19;5(2):e9329. doi: 10.1371/journal.pone.0009329 (PMC2824835; doi:10.1371/journal.pone.0009329)
Supplement: Appendix S1 — Description of measured sections. (0.17 MB DOC) [file pone.0009329.s001.doc]

**APPENDIX: MEASURED SECTIONS**

Although we made use of several sections provided by previous authors, most of these were problematic to use due to the difficulty in identifying described units precisely in outcrop. We have therefore measured most of the sections used in this study. We provide a key for the measured sections (Figure S2), and the sections themselves (Figures S3-S10) are described from the bottom up, and we provide labeled photographs in this Appendix to aid future researchers in identifying our units in outcrop. The colors for mudstones and muddy very fine-grained sandstones are specified using the Munsell color system. In the outcrop photos, dashed lines are used to highlight the edges of features such as hills and ridges which may be partially blocking outcrops behind them.

We have also provided UTM coordinates for the outcrops, excepting vertebrate and plant localities (which are proprietary information of the National Park Service, and only available to qualified researchers). Coordinates were taken with a Garmin GPSmap 60, using the NAD 27 datum. The coordinates are accurate to within 10 meters.

Although this study was primarily concerned with the Sonsela Member, additional sections (Upper Flattops West, Lower Flattops West, Tepees to Camp’s Butte) were included to encompass the stratigraphic interval containing all known plant and vertebrate localities in the southern part of the park. This covers most of thickness of the Chinle Formation in this region except for the lower part of the Blue Mesa Member below the Newspaper Rock Bed, and the top of Gregory’s [34] “D unit,” which are exposed in the area encompassing the Tepees, Haystacks, and Newspaper Rock.

1. South End Knob section (total thickness: 9.05 m)

Within the traditional park boundaries, the exposures west of Rainbow Forest are the only ones south of Flattops to expose a complete section through the Lot’s Wife beds and Camp Butte beds, as well as the top of the Blue Mesa Member. Two sections were measured near the south entrance station. The South End Knob section (Figure S3a-b) is located at 12S E0602076 N3851723 NAD 27. The South End Cliff section (Figures 10h, Figures S3c-d) is located just the west, and measured from 12S E0601939 N3851827 NAD 27. This section is capped by a conglomeratic sandstone (unit 5) which can be traced north to the type section of the Rainbow Forest Bed at the “Giant Logs” section of Heckert and Lucas [11]. There is also fairly striking conglomeratic lens (unit 3) composed of extrabasinal clasts within the Lot’s Wife beds here which is not seen elsewhere, probably representing a lower tongue of the Rainbow Forest Bed.

Blue Mesa Member

1. 0-1.3 m (thickness: 1.3 m): Medium bluish gray (5B 5/1) siltstone showing very frothy weathering. Upper contact sharp.

Camp Butte beds

1. (2a) 1.3-2.5 m (thickness: 2.5 m): Extremely muddy medium- to coarse-grained sandstone, very frothy weathering. Compositionally and texturally immature; subrounded to subangular clasts, dominated by quartz, but with common feldspar and mica (the rest of sandstones in unit 2 are lithologically similar). Extremely colorful with intense light-brown (5YR 5/6), dark yellowish-orange (10YR 6/6), and grayish-yellow (5Y 8/4) streaks and mottling, contains carbonized plant material and white non-agatized petrified wood. Pockets of gypsum less than 1 mm across are common. Upper contact gradational.

(2b) 2.5-3.25 m (thickness: 0.75 m): Gray medium- to coarse-grained sandstone, gradually fining upwards into fine- to very fine-grained sandstone containing a conglomeratic layer about 10 cm thick with subangular to subrounded chert pebbles about 1 cm in diameter. The unit is capped by a few centimeters of yellowish-gray (5Y 8/1) siltstone. Pockets of gypsum a few mm in diameter are common. Upper contact sharp.

(2c) 3.25-4.25 m (thickness: 1 m): Fairly muddy coarse-grained sandstone, contains scattered tiny pockets of gypsum as in previous units, layer about 10 cm thick containing some scattered pebble-sized clasts about 1 cm in diameter near the base. Upper contact sharp.

(2d) 4.25-7.55 m (thickness: 3.3 m): Muddy coarse- to very coarse-grained sandstone with scattered angular pebble-sized (about 1 cm in diameter) rip-up clasts of gray mudstone and chert at the base, grading up into coarse-grained sandstone containing some granule-sized clasts (most a few mm in diameter). Streaks and mottles of light-brown (5YR 5/6), dark yellowish-orange (10YR 6/6), and grayish-yellow (5Y 8/4). This unit grades laterally into yellowish- gray (5Y 8/1) siltstone. Orange non-agatized petrified logs are present in the general area weathering out of this unit. Upper contact sharp.

(2e) 7.55-9.05+ m (thickness: 1.5+): About 30 cm of conglomerate, pebble-sized (about 1 cm in diameter) subangular to subrounded clasts of chert in a matrix of coarse- to very coarse-grained sandstone, grading up into medium- to coarse-grained sandstone. Unit not complete, upper part stripped by erosion.

1. South End Cliff section (total thickness: 26.2 m)

Camp Butte beds

1. 0-3.1 m (thickness: 3.1 m): Medium- to coarse-grained sandstone, compositionally and texturally immature as with the sandstones of unit 2 in the South End Knob section. Shows “candy-striping” of light gray, pink, and purple banding in weathered outcrop; fresh samples show a very fine-scale mottling with varying proportions of these colors probably representing differential amounts of interstitial hematite and/or clay. Upper contact sharp. Mostly equivalent to the upper part of unit 2 in the South End Knob section.

Lot’s Wife beds

1. (2a) 3.1-4.2 m (thickness: 1.1 m): Grayish-red-purple (5RP 4/2) siltstone with fine yellowish-gray (5Y 8/1) mottling and dark yellowish-orange (10YR 6/1) silicified rhizoliths. Upper contact sharp.

(2b) 4.2-6.1 m (thickness: 1.9 m): Finely mottled dark red and gray medium- to coarse-grained sandstone, “candy stripped” as in unit 1 though more strongly reddish near the base, locally well-lithified. Upper contact sharp.

(2c) 6.1-8.4 m (thickness: 2.3 m): Coarse-grained sandstone, grading up into medium- to fine-grained sandstone, whole unit fairly uniform dull purple color. Upper contact gradational.

(2d) 8.4-10.5 m (thickness: 2.1 m): Light brownish-gray (5YR 6/1) matrix-supported very fine- to fine-grained sandstone exhibiting popcorn weathering. Upper contact sharp.

1. Conglomeratic sandstone layer within the Lot’s Wife beds, generally coarse- to very coarse-grained sandstone with scattered granule- to cobble-sized clasts of chert, quartzite, and volcanic rock mostly up to about 10 cm in diameter, although there are some which reach 20 cm or more. The larger well-rounded cobble-sized clasts tend to be concentrated into lenses, though even these are “matrix-supported” in that the cobbles are separated by sand and granule- to pebble-sized clasts. At least the cobble-sized clasts may represent silicified limestone, although we were not able to identify any distinctive Paleozoic fossils as are common in the Rainbow Forest Bed and Jasper Forest bed (e.g., [16]).

(3a) 10.5-12.8 m (thickness: 2.3 m): Lighter colored and less resistant than 3b; sandstone is very finely-mottled red and white, there is a particularly thick (about 60 cm) layer of cobble-sized clasts near the middle of the unit. Upper contact gradational.

(3b) 12.8-15.2 m (thickness: 2.4 m): Dark red (probably due to interstitial hematite), fairly resistant ledge-forming unit, otherwise compositionally similar to unit 3a; faintly discernable horizontal planar bedding. Upper contact gradational.

1. (4a) 15.2-18.3 m (thickness: 3.1 m): Medium- to coarse-grained sandstone fining up into moderate brown (5YR 4/1) very fine-grained sandstone, “candy striping” of light and dark brown throughout the unit. The very top of the unit is a zone of pedogenic alteration about 10-20 cm thick, well cemented with intense pale-purple (5P 6/1) and light greenish-gray (5GY 8/1) mottling. Upper contact gradational.

(4b) 18.3-22.4 m (thickness: 4.1 m): Well-lithified siltstone, grading from grayish-red-purple (5RP 4/2) to moderate brown (5YR 3/4), some light greenish-gray (5GY 8/1) mottling. Upper contact sharp.

Rainbow Forest Bed

1. (5a) 22.4-23.7 m (thickness: 1.3 m): Very pale yellowish medium- to coarse-grained matrix-supported sandstone, with granule and pebble-sized clasts (up to about 1 cm in diameter) of chert and reworked mudstone scattered throughout, especially in the lower 20-30 cm. Upper contact sharp.

(5b) 23.7-26.2+ m (thickness: 2.5+ m): Very pale yellowish conglomeratic sandstone, lower part full of well-rounded cobble-sized clasts (generally about 5-7 cm in diameter; very few approach 10 cm) of chert, quartzite, and volcanic rock, becomes more sand-dominated higher in the unit, although lenses of pebble- and cobble-sized clasts persist. This unit contains colorful agatized petrified wood. Upper surface stripped by erosion.

1. PFNP-14 section/Giant Logs section (total thickness: 43 m)

This outcrop (Figures 11a, Figures S3e-f), located at 12S E0602800 N3854095 NAD 27 represents Heckert and Lucas’ [11] type section for the Rainbow Forest Bed, Jim Camp Wash “Bed,” and “Agate Bridge Bed” (a Flattops One sandstone), and was also previously measured by Roadifer ([17], section PFNP-14). Roadifer [17] tended to identify visually distinctive and easily identifiable units in his measured sections, and the units given in Figure correspond to his. We had much greater difficulty identifying the units given in Heckert and Lucas’ [11] “Giant Logs” section for the Jim Camp Wash beds. However, the Jim Camp Wash beds are a fairly complex unit of mudstone of sandstone which shows enormous lateral variation, and it may be that Heckert and Lucas’ units were measured at a slightly different location along the outcrop.

The bluish mudstones exposed at the base of the section (unit 1 to both Roadifer and Heckert and Lucas) were identified by Heckert and Lucas [11] as the top of the Blue Mesa Member, but in fact represent the top of the Lot’s Wife beds. The type of the Rainbow Forest Bed (unit 2 to both Roadifer and Heckert and Lucas) was identified as the “Sonsela sandstone bed” by Roadifer. Roadifer’s units 3, 5, and 6-7 are generally fining upward sequences within the Jim Camp Wash beds in which sandstone fine upwards into reddish or gray mudstone. To the north (to the right side of Figure 16e), the lower part of unit 5 becomes a slightly more resistant light-light colored sandstone which cuts down section to truncate unit 4 and the top of unit 3. Roadifer’s unit 4 is a dull-brown resistant bed identified as a conglomerate by Roadifer, probably due to the fact that it weathers into pebble-sized nodules. However this is a silificied horizon, and probably represents an unusually colored outcrop of “persistent reddish silcrete”, as it lies at the right stratigraphic distance above the Rainbow Forest Bed. Roadifer’s unit 8, identified by him as the “Camp Wash zone,” corresponds to Heckert and Lucas’ ([11], units 11-15) type of the “Agate Bridge Bed,” and is a lower Flattops One sandstone. This unit was used by us to define the base of the Martha’s Butte beds, and can be traced (with difficulty) around the Jim Camp Wash drainage.

1. East of Petroglyph Site section (total thickness: 29.85 m)

This section was measured along the west side of Jim Camp Wash northeast of Rainbow Forest, on a small butte at 12S E0604665 N3854142 NAD 27 (Figure S3g-h) slightly north of Roadifer’s [17] PFNP-6 section (the Pronghorn Petroglyph section of Heckert and Lucas [11]). This site was chosen primarily for its suitability for magnetostratigraphic sampling (Kate Zeigler, unpublished data), and provides a section through most of the Jim Camp Wash beds, missing only the lowermost and uppermost few meters.

Jim Camp Wash beds

1. 0-1.75 m (thickness: 1.75 m): Extremely muddy, matrix-supported, slightly lithified, very fine- to fine-grained sandstone, grayish red (10R 4/2) with very fine (mm scale) light greenish-gray (5GY 8/1) mottles. There are also a few larger (cm scale) mottles, usually forming around pockets of fine- to medium-grained sand, and similarly-sized pockets of dark purple mudstone. Becomes mottled pale red (10R 6/2) and light greenish gray higher up, and eventually almost solid light greenish gray in the uppermost 20-30 cm. The “persistent reddish silcrete” lies in the top of this unit, sometimes as a thick (~10 cm thick) silicified layer (Figure 14a), and sometimes only as a thin network of reddish silica veins extending through the top of unit 1. The outside is of the silcrete is moderate reddish brown (10R 4/6), while the inside is milky white and gray. To the south, the silcrete lies within the grayish-red-purple mudstone of unit 2, which is thicker there. Slope-forming unit. Upper contact gradational.
2. 1.75-3.45 m (thickness: 1.7 m): Grayish-red-purple (5RP 4/2) siltstone with sparse patches and sub-horizontal stringers of light gray mottling, which tend to form around patches of sand. Slope-forming unit. Upper contact sharp.
3. (3a) 3.45-4.75 m (thickness: 1.3 m): Muddy but clast-supported granule and pebble conglomerate (clast size up to ~1 cm). Clasts mostly re-worked pedogenic carbonate nodules.

(3b) 4.75-5.15 m (thickness: 0.4 m): Pale purple (5P 6/2) and gray banded muddy very fine-grained sand, laterally grading into well-lithified mudstone. Upper contact sharp.

1. Long Logs sandstone, or at approximately the same stratigraphic level.

(4a) 5.15-6.55 m (thickness: 1.4 m): Resistant lenses of well-lithified conglomerate with clasts of re-worked pedogenic carbonate, mostly granule-size, up to a few mm across, interbedded with friable clast-supported medium- to coarse-grained quartzose sandstone, and lenses of ripple cross-laminated very coarse-grained sandstone. The weathered surface of this unit is dark brown and tan. Upper contact gradational.

(4b) 6.55-9.65 m (thickness: 3.1 m): Well-lithified coarse- to very coarse-grained clast-supported sandstone as in unit 4a, faint horizontal planar-bedding, gently dipping planar cross-bedding, and trough cross-bedding, fines upwards into medium- to coarse-grained sandstone, which is well-lithified and weathers into “hoodoos,” light gray with faint reddish and purple banding, light greenish-gray zone near the top. There are a few discontinuous lenses of granule conglomerate as in unit 4a. Upper contact gradational.

1. (5a) 9.65-11.75 m (thickness: 2.1 m): Moderate brown (5YR 3/4) siltstone with light greenish-gray (5GY 8/1) mottles, usually representing slightly sandy patches. Upper contact sharp.

(5b) 11.75-12.35 m (thickness: 0.6 m): Light gray fine- to medium-grained sandstone, lower part mixed with moderate brown (5YR 3/4) mudstone. This unit cuts sharply down-section to the north, and is the gray hoodoo-weathering sandstone referred to informally as the “Bowman sandstone” which makes up most of the Bowman South section, and is exposed near the base at Bowman 1 (units 2a and 2b) and Bowman 3 (units 1a and 1b).

(5c) 12.35-14.55 m (thickness: 2.2 m): Well-lithified grayish-red-purple (5RP 4/2) siltstone with light gray mottling, grades up into moderate brown (5YR 3/4).

(5d) 14.55-21.95 m (thickness: 7.4 m): Light gray very fine-grained sandstone, mottled with reddish-brown pockets of mudstone which become more purplish higher in the section. The unit grades upwards into pale purple (5PB 5/2) and light greenish-gray (5GY 8/1) mottled very fine-grained sandstone and silt. The top two meters of the unit is heavily pedogenically altered, full of plum-sized carbonate nodules and stringers of calcified rhizoliths (Figure 13a). Upper contact sharp.

1. 21.95-23.65 m (thickness: 1.7 m) Medium- to coarse-grained sandstone, horizontal planar bedding and gently dipping planar cross-bedding, weathered surface tan-colored. Patches of granule and pebble conglomerate similar to unit 4a at the base of the unit. Resistant ledge-forming unit. Upper contact gradational.
2. 23.65-29.85 m (thickness: 6.2 m): Very fine-grained sand to silt. Light gray sand at the base followed by alternating sand and silt-dominated layers, all somewhat well-lithified. Pale reddish-brown (10R 5/4) and moderate reddish- brown (10R 4/6), with pockets of grayish-red-purple (5RP 4/2) and dark greenish-gray (5G 4/1) mottling throughout. Unit not complete, upper surface stripped by erosion, but upper contact with base of Flattops One sandstone only about 2 meters higher in adjacent cliff. Roughly equivalent to unit 4 of Bowman 3 section.
3. Bowman 2 (Bowman South) section (total thickness: 9.35 m)

The Bowman vertebrate localities are located northeast of Rainbow Forest on the west side of the Jim Camp Wash drainage, just north of the East of Petroglyphs section. The Bowman South locality is near a small knob formed mostly from the Bowman sandstone (Figure S4a-b) located at 12S E604866 N3854341 NAD 27, while the Bowman locality proper is located near a small mesa (Figure S4e-f) located at 12S E604831 N3854555 NAD 27. Several vertebrate localities can be correlated to this knob and mesa respectively, whereas the cliff at 12S E604793 N3854410 NAD 27, measured for the Bowman 3 section (Figures S4c-d) covers most of the thickness of the Jim Camp Wash beds starting not far above the persistent red silcrete zone, and the lower Martha’s Butte beds, and can be tied to both of the other sections.

Jim Camp Wash beds (Bowman sandstone)

1. 0-0.5 m (thickness: 0.5 m): Slightly sandy siltstone, grayish red-purple (5RP 4/2), with yellowish gray (5Y 8/1) mottling. Contains pedogenic carbonate nodules. Friable slope-forming unit. Upper contact sharp.
2. 0.5-3.75 m (thickness: 3.25 m): Very friable coarse- to very coarse-grained, poorly sorted sandstone with subangular to subrounded clasts, mica occurs as a common accessory. Becomes extremely muddy sandstone near the top of the unit, where the vertebrate material was collected. Fresh exposures are dark yellow with orange mottling, weathered exposures are yellow-tan. Friable slope-forming unit.
3. (3a) 3.75-4.45 m (thickness: 0.7 m): Complexly interbedded muddy sandstone and conglomerate. Sandstone is muddy, coarse-grained, well-sorted, with subangular to subrounded clasts. The conglomerate is muddy and composed of granule- to pebble-sized clasts up to 2 cm in diameter of quartz, reworked mudstone, and reworked pedogenic carbonate nodules. Upper and lateral contact with unit 3b gradational.

(3b) 4.45-8.15 m (thickness: 3.7 m): Similar to unit 3a except that the conglomeratic lenses are absent, and there are well-cemented lenses of fine- to medium grained sandstone which horizontal planar bedding and gently dipping cross bedding, which weather into “hoodoos.” Upper contact gradational.

1. 8.18-9.35 m (thickness: 1.2 m): Grayish-red (10R 4/2) siltstone with light greenish-gray (5GY 8/1) mottling and slickensides, upper part contains black carbonized streaks and pedogenic carbonate, becomes yellowish medium- to coarse-grained sand at the very top. Friable slope-forming unit. Unit not complete, upper contact stripped by erosion.
2. Bowman 3 section (total thickness: 30.3 m)

Jim Camp Wash beds

1. 0-2.4 m (thickness: 2.4 m): Very fine-grained greenish-gray sandstone fining up into grayish-red (10R 4/2) siltstone with light olive-gray (5Y 6/1) mottling at the top. Slope-forming unit. Upper contact sharp. Base of unit not exposed.
2. (2a) 2.4-2.5 m (thickness: 0.1 m): Base of Bowman sandstone. Very well-cemented coarse- to very-coarse grained sandstone with some pebble-sized clasts (up to 1 cm in diameter) mixed in. Pebble-sized clasts composed of reworked mudstone and pedogenic carbonate nodules. Resistant ledge-forming unit.

(2b) 2.5-6.2 m (thickness: 3.7 m): Upper part of Bowman sandstone. Dull lavender friable muddy medium- to very coarse-grained sandstone, pebble-sized clasts (up to a few cm in diameter) of reworked mudstone scattered throughout, contains some pockets of mudstone. Fines upwards into light gray fine- to very fine-grained sandstone, then back into coarser lavender sand and gravel. Upper contact gradational. Equivalent to unit 1 in the Bowman 1 section.

(2c) 6.2-10 m (thickness: 3.8 m): Very muddy fine-grained sandstone, mottled lavender and light gray. Grades up into pale purple (5P 6/2) silt to very fine-grained sand with light greenish-gray (5GY 8/1) mottling and some reworked pedogenic carbonate nodules. Unit becomes darker grayish-purple (5P 4/2) and very well-cemented in the top 20 cm. Mostly slope-forming unit. Upper contact sharp. Equivalent to unit 4 of the Bowman 3 (Bowman South) section.

1. (3a) 10-10.2 m (thickness: 0.2 m): Light greenish-gray (5GY 8/1), very muddy matrix-supported pebble conglomerate, clasts of reworked mudstone up to 2 cm in diameter. Equivalent to unit 4 of Bowman 1 section.

(3b) 10.2-10.5 m (thickness: 0.3 m): Light greenish-gray, well-cemented, pebble conglomerate. Pebble clasts composed of reworked mudstone, pedogenic carbonate, and chert up to 3-4 cm in diameter. Resistant ledge-former.

(3c) 10.5-10.9 m (thickness: 0.4 m): Light greenish-gray, poorly sorted, muddy coarse-grained sand. Upper contact gradational.

1. 10.9-18.2 m (thickness: 7.3 m): Pale purple (5P 6/2) silt to very fine-grained sandstone with very fine greenish-gray (5GY 8/1) mottling, becomes more of a brownish shade higher up, then lavender about 5 m above base of unit, then a light greenish-gray conglomeratic layer with pebble-sized clasts of reworked pedogenic carbonate nodules. Slope-forming unit. Upper contact sharp.

Martha’s Butte beds

1. Lower Flattops One sandstone, can be traced to Heckert and Lucas’ [11] “Giant Logs” section, where it is the type of the “Agate Bridge Bed.”

(5a) 18.2-20.6 m (thickness: 2.4 m): Light gray, very muddy and poorly sorted coarse-grained sandstone, fining up into a friable, less muddy and better sorted medium- to coarse-grained sandstone containing well-cemented lenses 4-5 cm thick. Upper contact gradational.

(5b) 20.6-24.1 m (thickness: 3.5 m): Greenish-gray, muddy but clast-supported, fairly well-sorted, medium-grained sandstone. Horizontal planar-bedding and ripple cross-lamination. Somewhat more resistant than previous unit and contains some layers that are very well-cemented. Upper contact gradational.

1. 24.1-28.8 m (thickness: 4.7 m): Muddy fine to medium-grained sandstone which fines upward into siltstone to very fine-grained sandstone. Mostly moderate brown (5YR 3/4) with light greenish gray mottling. Poorly exposed.
2. Another lower Flattops One sandstone: 28.8-30.3 m (thickness: 1.5 m): Very well-cemented, well-sorted, fine to medium-grained sandstone. Very faint horizontal planar beds and planar cross-bedding, with some ripple cross-lamination. Light gray in fresh outcrop but weathers to tan. Upper surface stripped by erosion.
3. Bowman 1 section (total thickness: 13.65 m)

Jim Camp Wash beds

1. 0-1.1 m (thickness: 1.1 m): Bowman sandstone. Matrix-supported medium- to coarse-grained sandstone with subangular clasts. Alternates between easily eroded layers with popcorn weathering and more resistant layers which form “hoodoo” ledges. Mostly very light gray (N8), although patches are grayish- orange (10YR 7/4) and pale greenish-yellow (10Y 8/2). Upper contact sharp.
2. (2a; lateral facies change into lower part of unit 2b) (thickness: 0.9 m): Light olive-gray (5Y 6/1), muddy, coarse-grained siliceous sandstone with subangular to subrounded clasts. Some interbedded reworked carbonate nodules. Very faint horizontal planar bedding, planar cross-bedding, and ripple cross-stratification. Slope-forming unit. Upper contact gradational.

(2b) 1.1-3.6 m (thickness: 2.5 m): Very friable, muddy, poorly-sorted, medium- to coarse-grained light greenish-gray (5GY 8/1) sandstone, grading upwards into light gray (N8) fine-grained sandstone. Slope-forming unit. Upper contact gradational.

1. 3.6-4.7 m (thickness: 1.1 m): Grayish red-purple (5RP 4/2) silt to very fine-grained sandstone with light greenish-gray (5GY 8/1) mottling, upper part becomes increasingly sandy. White (N9) reworked pedogenic carbonate nodules are scattered throughout. Slope forming unit. Upper contact sharp.
2. 4.7-5.9 m (thickness: 1.2 m): Granule to pebble conglomerate, largest clasts up to 1 cm in diameter, with some interbedded fine- to medium-grained sandstone. Conglomerate clasts mostly re-worked pedogenic carbonate nodules, although some are reworked mudstone. Resistant ledge-forming unit. Upper contact gradational.
3. (5a) 5.9-7.3 m (thickness: 1.4 m): Very muddy, poorly sorted, fine- to medium-grained sandstone with greenish-gray mottling, interbedded in the lowermost 10 cm with resistant lenses of light gray medium- to coarse-grained sandstone. Slope-forming unit.

(5b) 7.3-8.0 m (thickness: 0.7 m): Lens of pinkish-gray fine- to medium-grained well-cemented sandstone. Resistant ledge-forming unit.

(5c) 8.0-8.25 m (thickness: 0.25 m): Same as unit 5a.

(5d) 8.25-13.45 m (thickness: 5.2 m) Grayish-red-purple (5RP 4/2) silt to very fine-grained sand with light greenish-gray (5GY 8/1) mottling, contains some pedogenic carbonate nodules which become less common in the upper part of the unit. Slope-forming unit.

1. 13.45-13.65 m (thickness: 0.2 m): Well-cemented coarse-grained clast-supported, well sorted sandstone with subangular to subrounded clasts, faint horizontal planar bedding and planar cross-bedding. Only a weathered remnant remains on top of the hill.
2. “No Name Point 3” section (total thickness: 14.98 m)

This section (Figures S4g-h) was measured by Woody [42] at a small butte in the middle of the Jim Camp Wash drainage, the base of which is at about 12S E603673 N3854544 NAD 27. Woody is correct that the sandstone exposed at the base of the section (unit A) is the Rainbow Forest Bed. The persistent reddish silcrete is locally absent here, but the ripple-bedded sandstone (unit E) is at roughly the same stratigraphic level. The coarse-grained sandstone capping the mesa (unit I) can be traced throughout this area, and in other neighboring buttes it lies several meters below the base of the same Flattops One sandstone forming the base of the Martha’s Butte beds in this area. The capping sandstone in this section is one of the several prominent sandstones in the Jim Camp Wash beds, but probably lies slightly above the Long Logs sandstone, possibly at the level of the Bowman sandstone.

1. “No Name Point 2b” (total thickness: 21.31 m)

This section (Figures S5a-b) was measured by Woody [42] at a prominent cliff on the eastern side of the Jim Camp Wash drainage, the basal conglomerate in the foreground is at 12S E0606203 N3854676 NAD 27. This is a particularly difficult region in which to correlate individual sandstone units, particularly the contact between the Jim Camp Wash beds and Martha’s Butte beds, is difficult to physically trace due to the complexity of the units. The basal contact of the Flattops One sandstone that Heckert and Lucas [11] made the type for the “Agate Bridge Bed” can be traced to the Bowman locality, and from there around the Jim Camp Wash drainage to just south of Red Butte, to where this sandstone becomes part of a massive amalgamated sandstone unit composed of multiple lower Flattops One sandstones (Figure 12b). From here, the basal contact can be traced to No Name Point 2b, where it lies at the base of Woody’s units E and F, consisting of interbedded greenish-gray sand and mudstone, while the rest of the massive amalgamated sandstone grades laterally into an upper ledge forming sandstone, a higher Flattops One sandstone (Woody’s unit H, the “Little Battleship sandstone”) and an intervening section dominated by mudstone. The conglomeratic unit at the base of the section (Woody’s unit A) may be close to the level of the Long Logs sandstone or Bowman sandstone. In summary, Woody’s units A-D lie in the Jim Camp Wash beds, while units E-H represent the Martha’s Butte beds.

1. Dalton Site section (total thickness: 25.1 m)

The Dalton Site section (Figures S5c-d) was measured at 12S E0606877 N3855141 NAD 27, not far from a vertebrate locality of the same name, and preserves almost the exact same section exposed at Martha’s Butte on the other side of the Flattops.

Martha’s Butte beds

1. 0-2 m (thickness: 2+ m): Lower Flattops One sandstone. Light olive gray (5Y 6/1) medium- to coarse-grained friable muddy sandstone, quartzose but with abundant muscovite and biotite. The unit is interbedded with lenses, usually only a few cm thick and dipping at angles of about 15-20°, of well-cemented medium- to coarse-grained sandstone with ripple cross-lamination. The upper part of the unit is also interbedded with very muddy grayish red purple (5RP 4/2) fine- to medium-grained sand. The base of the unit is not exposed at the locality. Upper contact gradational.
2. 2-10.1 m (thickness: 8.1 m): “Candy-striped beds.” Fluctuates between siltstone, often well-lithified (especially in the lower part of the unit), and very fine-grained sandstone. The colors in the unit consist of mottled and banded pale red purple (5RP 6/2) and yellowish gray (5Y 8/1), with the former tending to be siltier and the latter tending to be sandier. Pedogenic carbonate nodules occur in the upper part of the unit, although there is not a “mottled purple zone” which can be clearly distinguished from the rest of the “candy-stripped beds” here as there is at Martha’s Butte. Upper contact sharp.

Petrified Forest Member

1. 10.1-20.4 m (thickness: 10.3 m): “Monotonous purple beds.” Muddy siltstone with popcorn weathering, fairly uniform grayish red purple (5RP 4/2) except for some fine yellowish gray (5Y 8/1) mottling. Upper contact sharp.
2. 20.4-25.1 m (thickness: 4.7 m): Flattops 2 Bed. Resistant, ledge-forming, fine- to medium-grained sandstone with horizontal planar bedding low in the unit, and horizontal planar bedding and planar cross-bedding high in the unit, with at least some of the latter large enough to possibly represent lateral accretion beds. Weathers light brown. Upper surface erosionally stripped, forms top of cliff.
3. Lower “Flattops West” section (total thickness: 16.95 m)

Heckert and Lucas [11] measured the Flattops sandstones numbers one through four of Billingsley [18] as a composite section, their Flattops West section. They designated type sections for Flattops sandstones numbers 2-4, which they called Flattops Beds 2-4. The lower part of the section, encompassing units 1-11, included the “Agate Bridge Bed” (a Flattops One sandstone) and the type section of Flattops Bed 2, and was measured just east of Red Butte. The upper part of the section, encompassing units 12-19 and extending from the top of Flattops Bed 2 to the top of Flattops Bed 4, was measured in two different areas of the Flattops, about 2 kilometers from where the lower part of the section was measured. We have re-measured units 12-19 in the same areas that Heckert and Lucas [11] did, as these sections therefore encompass the type sections of Flattops Bed 3 and Flattops Bed 4. Lower Flattops West (Figure S5e-f) was measured at 12S E0607645 N3854991 NAD 27, and Upper Flattops West (Figure S5g-h) was measured at 12S E0607767 N3855109 NAD 27. The numbering system for units is taken from Heckert and Lucas’ [11] measured section, with some modifications.

Petrified Forest Member

1. 0-1.3 m (thickness: 1.3+ m): Flattops Bed 2. Resistant, ledge-forming gray, reddish-brown, and light brown very fine- to fine-grained sandstone with faint horizontal planar bedding and ripple cross-lamination, interbedded with very muddy dark reddish-brown very fine-grained sandstone. Only the top of the unit is exposed. Upper contact gradational.
2. 1.3- 5.1 m (thickness: 3.8 m): Fining upward sequence, dark reddish-brown muddy very fine- to fine-grained sandstone with thin (10 cm or less thick) beds of well-cemented light gray very fine- to fine-grained sandstone, grading up into reddish-brown siltstone. About a meter above the base of the unit is a zone of pebble-sized pedogenic carbonate nodules. Friable slope-forming unit. Upper contact gradational.
3. (13a) 5.1-9.6 m (thickness: 4.5 m): Light greenish-gray fine- to very fine-grained sandy mudstone. Heckert and Lucas [11] observed carbonaceous plant debris in this unit. Friable slope-forming unit. Upper contact sharp.

(13b) 9.6-11.35 m (thickness: 1.75 m): Friable slope-forming light yellowish- gray extremely muddy coarse-grained sandstone with interbedded lenses of resistant clast-supported horizontal planar-bedded sandstone about 10 cm thick. Upper contact sharp.

1. 11.35-12.95 m (thickness: 1.6 m): Flattops Bed 3. Complexly interbedded unit, dominated by resistant, ledge-forming, horizontal planar bedded and trough cross-bedded coarse- to very coarse-grained sandstone, interbedded low in the unit with granule conglomerate with clasts composed mostly of reworked pedogenic carbonate, and higher in the unit with muddy, friable coarse- to very coarse-grained sand. Upper contact sharp.
2. 12.95-16.95 m (thickness: 4 m): Flattops Bed 3. Resistant, ledge-forming, horizontal planar bedded, planar cross-bedded, and trough cross-bedded coarse- to very coarse-grained sandstone, with a few thin lenses of granule conglomerate less than 10 cm thick. Upper surface stripped by erosion, forms top of cliff.
3. Upper “Flattops West” section (total thickness: 13.65 m)

Flattops Bed 3

1. 0-0.25 m (thickness: 0.25+ m): Light gray, resistant, medium-grained horizontal planar-bedded sandstone. Only the top of the unit is exposed. Upper contact gradational.

Petrified Forest Member (overbank deposits)

1. 0.25-4.05 m (thickness: 3.8 m): Fining upward sequence. Chocolate brown and dark reddish-brown muddy fine-grained sandstone, with well cemented light gray layers generally less than 10 cm thick, grading up into dark reddish-brown siltstone, and then silty claytone of the same color, containing fine greenish-gray mottles. There is a zone of unusually large (plum-sized) pedogenic carbonate nodules about a meter above the base of the unit. This is probably the lower of the two “conglomerates” that Heckert and Lucas [11] identified, although they did recognize the presence of pedogenic carbonate nodule horizons within this unit. Upper contact sharp.
2. (17a) 4.05-4.55 m (thickness: 0.5 m): Discontinuous lenses of conglomerate alternating between layers dominated by granule-sized and pebble-sized clasts, clasts composed primarily of reworked intrabasinal pedogenic conglomerate, horizontal planar bedded and planar cross-bedded.

(17b) 4.55-11.05 m (thickness: 6.5 m): Fining upward sequence similar to unit 16. The lower part of the unit is reddish-brown muddy fine-grained sandstone with well-cemented light gray layers and lenses (about 10-20 cm thick) of well-cemented, medium-grained, ripple cross-laminated sandstone. This fines up into siltstone, then into dull reddish-gray calcareous muddy siltstone. Upper contact sharp.

(17c) 11.05-11.95 m (thickness: 0.9 m): Light gray friable coarse-grained sand and interbedded siltstone. The base of the unit is a pebble conglomerate composed primarily of clasts of reworked pedogenic carbonate. The upper contact is sharp.

Flattops Bed 4

1. 11.95-13.65 m (thickness: 1.7 m): Dark brown, ledge-forming medium- to coarse-grained sandstone with extensive horizontal planar bedding, planar cross-bedding, and ripple cross-lamination.
2. Dry Wash Bridge East section (total thickness: 16.5 m)

This section (Figures S6a-b) was measured at 12S E0608669, N3856310 NAD 27, on the north side of one of the mesas between Martha’s Butte and the bridge crossing Dry Wash along the main park road, just north of the Flattops. This particular location was selected as it encompasses not only the entire section of the Martha’s Butte beds, but some of the underlying Jim Camp Wash beds, including a locally prominent conglomeratic unit (unit 2) which may be roughly equivalent to the Peninsula sandstone.

Jim Camp Wash beds

1. 0-1.2 m (thickness: 1.2 m): Extremely muddy fine to medium-grained sand, grayish red purple (5RP 4/2) with light greenish gray (5GY 8/1) mottling. Upper contact sharp.
2. 1.2-1.9 m (thickness: 0.7 m): Well indurated ledge-forming conglomeratic sandstone, dominated by granule- to pebble-sized clasts most of which are no larger than 1 cm, derived from reworked carbonate nodules, interbedded with fine to medium-grained sand with horizontal planar bedding and gently dipping planar cross-bedding. Upper contact gradational. This unit may be roughly equivalent to the Peninsula sandstone.
3. 1.9-4.9 m (thickness: 3 m): Poorly sorted medium- to coarse-grained sand fining up to fine- to medium-grained sand with grayish red purple (5RP 4/2) mud mixed in, both as matrix and reworked granule or pebble-sized clasts. Interbedded in the lower part of the unit are thinner (usually 1-3 cm thick) well-indurated lenses of medium- to coarse-grained sand, often ripple cross-laminated, sometimes with granule-sized clasts (a few millimeters in diameter) at the base. The top of the unit is a well-cemented discontinuous layer of poorly sorted sand and granule-sized clasts, up to about 10 cm thick. Upper contact sharp.
4. 4.9-6.4 meters (thickness: 1.5 meters): Silt to very fine-grained sand, grayish red purple (5RP 4/2) with light greenish gray (5GY 8/1) mottling, pedogenic carbonate nodules dominate the upper part of the unit. Upper contact sharp.

Martha’s Butte beds

1. 6.4-13.3 meters (thickness: 6.9 meters): Friable medium- to coarse-grained sand, usually poorly sorted, alternating between clast-supported and matrix-supported layers, the latter of which sometimes show popcorn weathering. Some of the matrix-supported sand is light olive gray (5Y 6/1), but the upper part of the unit is mostly pale red (10R 6/2). The sand at the base of the unit, which grades laterally into pale-purple (5P 6/2) mudstone, is equivalent to the lower Flattops One sandstone forming the flats to the west and exposed at the base of Martha’s Butte. The rest of the section is equivalent to the “candy-striped beds.” Upper contact sharp. On the east side of this mesa, this lower Flattops One sandstone is a mostly well-indurated unit about 2.5 meters thick, composed of medium- to coarse-grained, poorly sorted, clast-supported sandstone composed mostly of subangular to subrounded quartz clasts, mostly trough cross-bedded although there are also planar cross-beds. There are conglomeratic beds with pebble-sized clasts usually 5 cm in diameter or less, composed mostly of reworked intrabasinal pedogenic carbonate clasts although chert and quartzite also occur. These conglomerate beds are usually no more than 10 cm thick, although some may reach 40 cm in thickness. This is a more typical expression of the lower Flattops One sandstone than seen in the measured section.
2. 13.3-13.9 m (thickness: 0.3 meters): Grayish blue (5PB 5/2) mudstone and muddy very fine-grained sand, mottled with yellowish gray (5Y 8/1), contains pedogenic carbonate nodules. This “purple mottled” unit is not very distinct here from the banded beds, although elsewhere it is thicker and separated from them by an erosional surface. Upper contact sharp, locally truncated by unit 7.
3. 13.9-15.3 m (thickness: 1.4 meters): Upper Flattops One sandstone. Conglomerate dominated by granule- and pebble-sized clasts composed of reworked pedogenic carbonate up to a few centimeters in diameter, interbedded with muddy very fine- to fine-grained sand. Upper contact sharp.
4. 15.3-16.5 meters (thickness: 1.2 meters): Upper Flattops One sandstone. Well indurated fine- to medium-grained siliceous sandstone, dominated by horizontal planar bedding and planar cross bedding, locally contains a few thin (10 cm thick or less) conglomeratic beds, containing pebble-sized clasts composed mostly of reworked pedogenic carbonate nodules. Nearby, this sandstone thickens to more than 3 meters. This sandstone marks the top of the Sonsela Member, and is the same one capping the line of mesas between the Dry Wash bridge along the main park road and Martha’s Butte.
5. Walker’s Stump Section (total thickness: 11.9 m)

Walker’s Stump (Figures S6c-d) is a low knob located near the foot of Martha’s Butte at 12S E0608292 N3856717 NAD 27. The knob represents a relatively friable facies of the lower Flattops One sandstone forming the base of Martha’s Butte and most of the flats below the Flattops in this area. It is named for the large orange petrified stump in the upper beds of the knob, which have also produced other important plant fossils.

Jim Camp Wash beds

1. 0-0.6 m (thickness: 0.6 m): Dull purple-gray, fairly well-cemented, clast-supported, fine- to medium-grained sand. Horizontal planar bedding. Upper contact gradational.
2. 0.6-2.1 m (thickness: 1.5 m): Light olive-gray (5Y 6/1), fairly friable, clast-supported fine-grained micaceous sandstone. Slope-forming unit. Upper contact gradational.
3. 2.1-3.2 m (thickness: 1.1. m): Pale purple (5P 6/2), muddy, very fine-grained sand, interbedded with thin discontinuous lenses of resistant, greenish-gray, fine- to medium-grained clast-supported sand about 10 cm thick. Zone of pedogenic carbonate nodules at the base of the unit. Faint planar cross-bedding. Upper contact sharp.

Martha’s Butte beds (lower Flattops One sandstone)

1. 3.2-6.2 m (thickness: 3.0 m): Very light gray (N8), muddy, very fine-grained sand, interbedded with more resistant lenses of coarse-grained sandstone about 20-30 cm thick with scattered pebble-sized clasts (about 1 cm thick) of reworked pedogenic carbonate nodules. This unit produces plant fossils.
2. 6.2-11.9 m (thickness: 5.7 m): Mostly light greenish-gray (5GY 8/1) medium- to coarse-grained friable sandstone, interbedded with slightly resistant lenses of fine- to medium-grained sand about 10 cm thick, and thinner lenses of muddy purple fine-grained sandstone about 1 cm thick.
3. Martha’s Butte Section (total thickness: 35.1 m)

Martha’s Butte (Figure 12a, Figures S6c-e), located at 12S E0608235 N3856775 NAD 27, serves as the primary reference section for the Martha’s Butte beds. It preserves a complete section through the Martha’s Butte beds (including lower and upper Flattops One sandstones bracketing the unit, with another lower Flattops One sandstone being present just to the north that is immediately above the one at Martha’s Butte), but the uppermost Jim Camp Wash beds and lowermost Petrified Forest Member.

Jim Camp Wash beds

1. 0-0.2 m (thickness: 0.2 m): Fine- to medium-grained sandstone, contains some reworked pedogenic carbonate nodules. Dark brown in fresh exposure, weathered surface purple and “frothy.” Slope-forming unit. Upper contact sharp.

Martha’s Butte beds

1. Lower Flattops One sandstone

(2a) 0.2-1.45 m (thickness: 1.25 m): Muddy, greenish-gray, fine- to medium-grained sandstone, fairly micaceous. Slope-forming unit. Upper contact gradational.

(2b) 1.45-4.95 m (thickness: 3.5 m): Light tan, clast-supported, medium- to coarse-grained sandstone, fairly micaceous. Slope-forming unit. Upper contact sharp.

1. Lower Flattops One sandstone

(3a) 4.95-6.25 m (thickness: 1.3 m): Well-cemented fine- to medium-grained sandstone. Horizontal planar beds and gently dipping planar cross-beds. Light gray in fresh outcrop, weathers tan. Resistant ledge former. Upper contact gradational.

(3b) 6.25-6.35 m (thickness: 0.1 m): Pebble conglomerate, most clasts about 1 cm in diameter although up to 5 cm, matrix of medium- to coarse-grained sand. Pebble clasts composed of quartz, chert, quartzite, reworked pedogenic carbonate. Upper contact gradational.

(3c) 6.35-6.85 m (thickness: 0.5 m) Same as unit 3a, interfingers with unit 3b. Slope-forming unit. Upper contact gradational.

1. 6.85-8.35 m (thickness: 2.1 m): “Candy-striped beds.” Friable very light gray (N8) silt to very fine-grained sandstone. Slope forming unit. Upper contact gradational.
2. 8.35-9.2 m (thickness: 0.85 m): “Candy-striped beds.” Banded and mottled reddish and gray, slightly cemented very fine- to fine-grained sandstone, interbedded with well cemented, resistant lenses of fine- to medium-grained sandstone about 3-4 cm thick. Upper contact gradational.
3. 9.2-17.8 m (thickness: 8.6 m): “Candy-striped beds.” Pale reddish-purple (10R 5/4), very muddy, very fine-grained friable sandstone, interbedded with bands of less muddy and better cemented light gray (N8) very fine-grained sand. Slope-forming unit. Upper contact gradational. The lower Flattops One sandstone capping the low buttes and the Peninsula just to the north is equivalent to one of the reddish zones in this unit.
4. 17.8-19.2 m (thickness: 1.4 m): “Purple mottled beds.” Zone of heavy pedogenic modification. Alternating light greenish-gray (5GY 8/1) very fine- to fine-grained sand, and pale red-purple (5RP 6/2) silt to very fine-grained sand, full of large (up to 10 cm in diameter) pedogenic carbonate nodules. Upper contact gradational.
5. 19.2-22.7 m (thickness: 3.5 m): “Purple mottled beds.” Pale red-purple (5RP 6/2) siltstone with light greenish-gray (5GY 8/1) mottling, contains light gray bands about 20 cm thick which are better cemented. Lower part has small pedogenic carbonate nodules (2-3 cm in diameter). Upper contact sharp.
6. 22.7-23.3 m (thickness: 0.6 m): Upper Flattops One sandstone. Well cemented pebble conglomerate, with most clasts composed of reworked pedogenic carbonate (although some are quartz), interbedded with fine-grained matrix-supported sandstone. Upper contact sharp.

Petrified Forest Member

1. 23.3-31.8 m (thickness: 8.5 m): Monotonous purple beds. Grayish red-purple (5RP 4/2) silt to very fine-grained sand, fairly well-lithified, some very fine greenish-gray mottling. Upper contact sharp.
2. 31.8-35.1 m (thickness: 3.3 m): Flattops 2 Bed. Well cemented, well-sorted, fine- to medium-grained sandstone, mostly horizontal planar bedded. Light gray in fresh outcrop, weathers brown. Top stripped, forms cap on butte.
3. Peninsula section (composite) (total thickness: 36.73 m)

The Peninsula is a northeasterly-trending cliff just north of Martha’s Butte which mostly formed by the Jim Camp Wash beds and the lowermost Martha’s Butte beds, and is capped by a lower Flattops One sandstone. This Flattops One sandstone pinches out into the “candy-striped beds” of the Martha’s Butte beds at Martha’s Butte (Figure 12a). Just to the northeast of the Peninsula, the section is exposed all the way down to the Jasper Forest bed. The section was measured as a series of micro-sections from the northeast to southwest at 12S E0608872 N3857800 NAD 27 (Figure S7a), 12S E0608701 N3857648 NAD 27 (Figure S7b), 12S E0608644 N3857522 NAD 27 (Figure S7c), and 12S E0608489 N3857317 NAD 27 (Figure S7d). These were combined into a composite section encompassing the entire Jim Camp Wash beds (Figure S7e), including the persistent red silcrete zone, Mountain Lion Mesa sandstone, and Peninsula sandstone. Where the same unit occurs in different micro-sections, the greater thickness was arbitrarily used for the composite.

Jasper Forest bed

1. 0-0.05 (thickness: 0.05 m): Medium- to coarse-grained, well-cemented clast-supported sandstone. Upper contact gradational. Only the top of the unit is exposed.

Jim Camp Wash beds

1. 0.05-0.4 (thickness: 0.35 m): Covered interval.
2. 0.4-2.15 m (thickness: 1.75 m): Sand mostly varying in clast-size from very fine- to medium- grained, and candy-striped moderate brown (5YR 4/4) and pinkish gray (5YR 8/1), with a few thin (few centimeter thick) layers of reddish silt to very fine-grained sand. Upper contact gradational. At the Mountain Lion Cliffs, this unit grades laterally in places into the top of the Jasper Forest bed.
3. 2.15-5.15 m (thickness: 3.0 m): Very dusky purple (5RP 2/2) siltstone with some very fine light greenish-gray (5GY 8/1) mottling. Upper contact gradational.
4. 5.15-8.85 m (thickness: 3.7 m): Persistent red silcrete zone. Yellowish-gray (5Y 8/1) siltstone with very dusky purple (5RP 2/2) mottling, containing two reddish silcrete horizons; the lower one is about 3-4 cm thick and 1.5 meters above the base of the unit, the second is a thicker ledge-former about 5-6 cm thick at the very top of the unit. The silcrete layers are mostly moderate reddish-brown (10R 4/6) though with milky white layers, and the outside is very dusky red (10R 2/2).
5. 8.85-10.35 m (thickness: 1.5 m): Grayish-purple (5P 4/2) and yellowish-gray (5Y 8/1) mottled siltstone, with a zone of pedogenic carbonate nodules. Upper contact gradational.
6. 10.35-13.15 m (thickness: 2.8 m): Grayish-red (10R 4/2) siltstone with fine gray mottles. Upper contact sharp.
7. 13.15-13.19 m (thickness: 0.04 m): Very well-cemented coarse-grained sandstone at mico-section 2 (Figure 19b), roughly laterally equivalent to moderate brown (5YR 4/4) calcrete at micro-section 3.
8. 13.19-14.59 m (thickness: 1.4 m): Muddy, very friable, drab greenish-gray coarse-grained sand interbedded with thin lenses (10-20 cm thick) of light olive gray (5Y 6/1) siltstone. Upper contact sharp.
9. 14.59-15.09 m (thickness: 0.7 m): Slightly resistant, clast-supported, grayish coarse-grained sand, with a 10 cm thick lens of gray siltstone in the middle of the unit, and a 20 cm thick layer of very muddy pebble conglomerate at the top, with the largest clasts mostly no larger than 2 cm and composed of chert. Upper contact gradational.
10. 15.09-20.69 m (thickness: 5.6 m): Light gray very fine-grained sand, candy-striped with light brownish gray (5YR 6/1) and light olive gray (5Y 6/1). The coarser-grained Mountain Lion Mesa sandstone is part of this unit and pinches out within it, but is better exposed along the edge of Starving Man Wash just to the north. This sandstone is a resistant and hoodoo-forming coarse-grained sandstone about 1.5 meters thick (the top of the unit is about 3 meters below the arbitrary top of the unit), with planar horizontal and cross beds, and trough cross beds. The upper part of the Mountain Lion Mesa sandstone is interbedded with the very fine-grained sand.
11. 20.69-22.69 m (thickness: 2 m): Grayish-red-purple (5RP 4/2) siltstone with popcorn weathering containing light greenish-gray (5GY 8/1) mottling and white carbonate nodules, some 2 cm or more across. Upper contact sharp.
12. 22.69-23.09 m (thickness: 0.4 m): Peninsula sandstone. Friable greenish-gray coarse-grained sandstone interbedded with resistant fine-grained sand with ripple cross-lamination, and granule conglomerate containing clasts about 2 mm in diameter, composed of reworked pedogenic carbonate. Upper contact gradational.
13. 23.09-25.19 m (thickness: 2.10 m): Light greenish-gray (5GY 8/1), very micaceous, muddy fine-grained sandstone. Upper contact gradational.

Martha’s Butte beds

1. 25.19-30.39 m (thickness: 5.2 m): Grayish-purple (5P 4/2) muddy, very fine-grained sandstone to silt mottled with light greenish-gray (5GY 8/1), pedogenic carbonate nodules of various sizes from millimeter scale up to two centimeters. The nodules are particularly concentrated in a layer about 1.5 meters above the base of the unit which contains variegated grayish-purple and light greenish-gray bands. This unit is laterally equivalent with the lower Flattops One sandstone at the base of Martha’s Butte. Upper contact gradational.
2. Lower Flattops One sandstone

(16a) 30.39-31.89 m (thickness: 1.5 m): Yellowish coarse-grained sandstone, locally friable and slope-forming. Upper contact sharp.

(16b) 31.89-31.94 m (thickness: 0.05 m): Very well cemented coarse-grained sandstone, slightly conglomeratic with granule-sized clasts of re-worked pedogenic carbonate nodules. Upper contact gradational.

(16c) 31.94-35.44 m (thickness: 3.5 m): Very friable, predominantly dark yellowish-orange (10 YR 6/6) extremely muddy coarse-grained sand, with abundant organic material concentrated mostly in patches of orange and black, contains non-agatized petrified wood.

(16d) 35.44-36.73 m (thickness: 1.3 m): Fairly resistant conglomeratic sandstone, coarse- to very coarse-grained sandstone with granule and pebble-sized clasts up to 2 cm or more in diameter, composed mostly of re-worked pedogenic carbonate and siltstone, although coal and petrified wood fragments are mixed in. Unit not complete at micro-section 4 (Figure 19d), upper part of unit stripped by erosion.

1. “Gatesy’s Plunge section 2” (total thickness: 23.43 m)

This section (Figures S7f-g) measured by Herrick [52] at 12S E0607320 N3858600 NAD 27 on top of the cliffs formed by the Flattops One sandstone forming Starving Man Cliffs and Mountain Lion Cliffs. This section forms the top of the exposures shown in Figure 12c. It is also approximately where Roadifer [17] measured his PFNP-7a section, and covers the uppermost Martha’s Butte beds and lowermost Petrified Forest Member. Herrick’s [52] units have been assigned an arbitrary numbering system, with unit 1a (0-2.20 m) representing the Flattops One sandstone, units 2a-e (2.20-10.58 m) representing the candy-striped uppermost Martha’s Butte beds (2e is the “purple mottled bed”), units 3a-d (10.5-20.79 m) being the “monotonous purple beds” at the base of the Petrified Forest Member, and unit 4 (20.79-23.43 m) being the Flattops 2 Bed.

1. “Gatesy’s Plunge section 4” (total thickness: 22.93 m)

This locality at 12S E0607655 N3858302 NAD 27 (Figures S7h-i), produced the *Zamites powelli* leaf impressions described by Herrick [52], which came from directly above the Mountain Lion Mesa sandstone. As with “Gatesy’s Plunge section 2”, Herrick’s units have been assigned an arbitrary numbering system, with unit 1 (0-4.75 m) representing the *Zamites-*producing beds in the upper Jim Camp Wash beds, immediately above the Mountain Lion Mesa sandstone (the top of which is exposed as a resistant sandstone here), units 2-5 (4.75-15.30 m) representing the upper part of the Jim Camp Wash beds, units 6-7 (15.30-20.27 m) representing either the uppermost Jim Camp Wash beds or the base of the Martha’s Butte beds (it is difficult to correlate this contact precisely in this area), and unit 8 (20.27-22.93 m) being the Flattops One sandstone capping the Mountain Lion Cliffs.

1. Mountain Lion Cliffs section (total thickness: 45.6 m)

This section (Figure 11d, Figures S8a-b), located at 12S E0608065 N3858693 NAD 27, is particularly critical, as it was measured where the traditional Sonsela sandstone bed (the Jasper Forest bed) can be traced to the base of cliffs east of Mountain Lion Mesa capped by a Flattops One sandstone, as traced previously by both Roadifer [17] and Herrick [52]. Roadifer [17] measured his PFNP-7a section here, but it was decided to measure a more detailed section, which encompasses the top of the Lot’s Wife beds, the entire thickness of the Jasper Forest bed and Jim Camp Wash beds, and the base of the Martha’s Butte beds (the Flattops One sandstone capping the section).

Lot’s Wife beds

1. (1a) 0-0.6 m (thickness: 0.6 m): Very dusky purple (5P 2/2) siltstone, mottled with yellowish gray (5Y 8/1). Upper contact sharp.

(1b) 0.6-4.2 m (thickness: 3.6 m): Extremely siliceous, slightly muddy, light gray medium- to coarse-grained sandstone, fining upwards into grayish red purple (5RP 4/2) silt to very fine-grained sandstone mottled with light greenish gray (5GY 8/1). The upper 40 cm is interbedded with light gray medium- to coarse-grained sandstone as at the base of the unit. Upper contact sharp.

Jasper Forest bed

1. 4.2-9.0 m (thickness: 4.8 m): Extremely siliceous, friable and muddy, medium- to coarse-grained sandstone, interbedded with resistant horizontal planar bedded and ripple-cross laminated medium- to coarse-grained sandstone weathering into “hoodoos”, and muddy pebble conglomerate with clasts composed mostly of chert and re-worked purple mudstone. The upper 1.5 meters is interbedded with grayish red purple (5RP 4/2) silt to very fine-grained sand. Upper contact gradational.

Jim Camp Wash beds

1. (3a) 9.0-16.8 m (thickness: 7.8 m): Complexly interbedded unit. Pinkish medium-grained sandstone with pebble-sized clasts (mostly no more than 2 cm) of chert scattered throughout, particularly on bedding surfaces, interbedded with light bluish medium-grained muddy sandstone and grayish blue (5PB 5/2) and light greenish gray (5GY 8/1) silt to very fine-grained sand. Horizontal and gently dipping planar bedding. There are two fining upward sequences grading from sand to silt in this unit. Upper contact gradational.

(3b) 16.8-20.9 m (thickness: 4.1 m): Silt to very fine-grained sand, grading from grayish-red-purple (5RP 4/2) to grayish-blue (5PD 5/2), with the upper 20 cm being light greenish-gray (5Y 8/1) and mixed with medium-grained sand. Upper contact sharp.

(3c) 20.9-29.7 m (thickness: 8.8 m): Mountain Lion Mesa sandstone. Muddy, light brown, medium- to coarse-grained sandstone, mostly trough cross bedded and horizontal and dipping planar bedded. There is some pebble conglomerate, mostly concentrated along bedding surfaces, with poorly rounded clasts of chert and reworked sedimentary rocks, the latter dominating. Upper contact gradational. The base of this sandstone locally truncates the persistent red silcrete zone here.

(3d) 29.7-37.8 m (thickness: 8.1 m): Generally fining-upward sequence. Medium- to coarse-grained sandstone as in previous unit, interbedded with brown, friable, fine- to medium-grained sand and dark yellowish-brown (10YR 4/2) siltstone forming “candy striped” layers, fining up into moderate brown (5YR 4/4) siltstone to very fine-grained sand, which becomes light olive gray (5Y 6/1) at the top of the unit. Upper contact sharp. It is possible that the upper part of this unit represents the base of the Martha’s Butte beds.

Martha’s Butte beds

1. (4a) 37.8-41.9 m (thickness: 4.1 m): Lower Flattops One sandstone. Yellowish, blocky weathering, medium- to coarse-grained sandstone with faint reddish banding and gently dipping planar bedding, probably representing lateral accretion bedding. Upper contact gradational.

(4b) 41.9-45.6 m (thickness: 3.7 m): Lower Flattops One sandstone. Extremely muddy dusky yellow (5Y 6/4) very fine-grained sand. Unit not complete, upper surface stripped by erosion.

1. Red Band Buttes section (total thickness: 16.5 m)

We refer to two buttes located just to the southeast of the Mountain Lion Cliffs (Figure S1, Figure 2) as the “Red Band Buttes” due to the distinctive reddish layer exposed on their sides. One of these buttes (Figure 11d, Figures S8c-d) at 12S E0608869 N3859185 NAD 27 was referred to as “Hill 5573” by Heckert and Lucas [11], and we have re-measured their section. The Red Band Buttes are important for several reasons. First, they are capped by the persistent red silcrete, which is thicker here (up to a meter) than anywhere else it has been examined within the park; Heckert and Lucas [11] had tentatively identified the silcrete here as the “Agate Bridge Bed” (Jasper Forest bed). Second, the actual Jasper Forest bed is almost non-existent here represented by the reddish band, which is composed of very fine-grained sand and silt. This identification is supported by examining the Peninsula section just to the south, where close to seven meters of section consisting of purple mudstone over gray and red candy-striped sand, lie between the silcrete horizons and the Jasper Forest bed as they do here (Figures S7a,f). Thirdly, the identification of the persistent red silcrete and Jasper Forest bed in the buttes allows the sandstones exposed below the eastern slopes of the mesas (including those around the Battleship Quarry) as lying in the lower part of the Lot’s Wife beds. These sandstones were identified by Heckert and Lucas [11] as the Rainbow Forest Bed in their “Hill 5573” section.

Lot’s Wife beds

1. 0-0.75 m (thickness: 0.75 m): Grayish-red-purple (5RP 4/2) siltstone mottled with light greenish gray (5GY 8/1). May be equivalent to the top if unit 2a from the Near Battleship Quarry section. Upper contact sharp.
2. 0.75-2.15 m (thickness: 1.4 m): Muddy light gray medium- to coarse-grained sandstone fining upwards into fine- to medium-grained muddy sandstone with pale purple (5P 6/2) mottling. May be equivalent to unit 2b from the Near Battleship Quarry section. Upper contact gradational.
3. 2.15-7.40 m (thickness: 5.25 m): Silt to very fine-grained micaceous sandstone, predominantly pale purple (5P 6/2) with light greenish gray (5GY 8/1) mottling, but with some layers of solid light greenish gray. Calcareous and in places well-lithified. Upper contact sharp.

Jasper Forest bed/Jim Camp Wash beds

1. 7.40-11 m (thickness: 3.6 m): Moderate red (5R 5/4) muddy very fine-grained sandstone with fine (millimeter scale) yellowish gray (5Y 8/1) mottling, fining upwards into silt to very fine-grained sand, which is candy striped and mottled dark reddish brown (10R 3/4) and light greenish gray (5GY 8/1). This lower part of this unit (the “red band”) is equivalent to the Jasper Forest bed, and the sandy-stripped finer grained upper part of the unit is equivalent to the lower Jim Camp Wash beds. Upper contact gradational.
2. 11-15.5 m (thickness: 4.5 m): Grayish-red-purple (5RP 4/2) siltstone with fine light greenish-gray (5GY 8/1) mottling. The top 20 cm of the unit is extremely calcareous and more intensely mottled with light greenish gray. Upper contact sharp.
3. 15.5-16.5+ m (thickness: 1+ m): Persistent red silcrete. Massive silicified horizon, predominantly shades of dull orange (grayish orange 10YR 7/4 and reddish orange 10R 6/6) but with layers of moderate reddish brown (10R 4/6), and pockets of extremely well-lithified pale purple (5P 6/2) mudstone.
4. Near Battleship Quarry section (total thickness: 4.3 m)

This section, measured at 12S E0609097 N3859146 NAD 27 (Figures S8c,e) lies near the Red Band Buttes between Mountain Lion Mesa, Agate Mesa, and the Battleship, and is a particularly problematic area for correlation. There are several sandstones, including some which are prominent ledge-formers, in the vicinity. One located near the Battleship Quarry contains a vertebrate locality, and another was identified as the “Rainbow Forest Bed” by Heckert and Lucas [11] in the lower part of their “Hill 5573” section. In fact, these sandstones appear to lie at the base of the upper Lot’s Wife beds, some distance below the Jasper Forest bed (for reasons explained above).

Lot’s Wife beds

1. 0-0.2 m (thickness: 0.2+ m): Top of the lower Lot’s Wife beds. Grayish-red-purple (5RP 4/2) siltstone. Upper contact sharp.
2. (2a) 0.2-3.5. m (thickness: 3.3 m): Upper Lot’s Wife beds. Fining upward sequence. Very siliceous, friable, medium- to coarse-grained clast-supported sandstone containing light brown (5YR 4/6) silicified rhizomes and vertebrate material. This sand immediately fines upwards into very micaceous very fine- to fine-grained muddy sandstone which is mottled white (N9), pale red (10R 6/2), and pale purple (5P 6/2) containing circular pockets of pale purple (5P 6/2) siltstone about 10-20 cm in diameter, probably representing rip-up clasts from the lower Lot’s Wife beds. The top 40 cm of the unit is pale purple (5P 6/2) siltstone. Upper contact sharp.

(2b) 3.5-4.3+ m (thickness: 0.8 m): Upper Lot’s Wife beds. Muddy, micaceous, light gray fine- to medium-grained sand, interbedded with lenses of pale purple (5P 6/2) and light greenish-gray (5GY 8/1) mottled siltstone about 10-15 cm thick. Unit incomplete, upper surface sharp.

1. Flag Canyon section (total thickness: 14.8 m)

The Flag Canyon vertebrate locality lies in the upper Lot’s Wife beds north of Crystal Forest, near this outcrop at 12S E0611631 N3859786 NAD 27. In this area (Figures S8f-g), the Jasper Forest bed retains its typical character as a prominent ledge-forming, highly siliceous conglomeratic sandstone, but a short distance to the south and southwest it grades laterally into a friable slope-forming sandstone, which is the character of the unit at Crystal Forest, and the Battleship (Figure 11g).

Lot’s Wife beds

1. 0-1.8 m (thickness: 1.8+ m): Fine- to medium-grained sandstone, slightly muddy although mostly clast-supported. Faintly horizontal planar-bedded and intensely candy-striped with yellowish gray (5Y 8/1), light greenish gray (5GY 8/1), pale red purple (5RP 6/2) and light brownish gray (5YR 6/1). The top 20 cm of the unit is grayish-red (10R 4/2) siltstone. Upper contact fairly sharp.
2. (2a) 1.8-2.6 m (thickness: 0.8 m): Light greenish-gray (5GY 8/1) and grayish- red (10R 4/2) silt to very fine-grained sandstone. The persistent orange silcrete lies at the top of the unit, and is a thin (few cm thick) and discontinuous light brown (5YR 5/6) silicified layer. Upper contact gradational and somewhat arbitrary.

(2b) 2.6-7 m (thickness: 4.4 m): Mottled siltstone, color varies between zones of yellowish gray (5Y 8/1), light olive gray (5Y 6/1), grayish blue (5PB 5/2) and grayish red purple (5RP 4/2). Upper contact sharp.

Jasper Forest bed

1. (3a) 7-8.3 m (thickness: 1.3 m): Medium- to coarse-grained horizontal planar-bedded sandstone, extremely siliceous and containing pebbly conglomeratic layers, mostly along bedding planes, but there are also discontinuous lenses of pebble conglomerate. Clasts are composed predominantly of chert. Upper contact gradational and arbitrary. The lower 20 cm of the unit is full of clasts of re-worked light brown (5YR 5/6) and yellowish-orange (10YR 6/6) mudstone, which give the base of the unit a bright orange color. Upper contact gradational.

(3b) 8.3-14.8+ m (thickness: 6.5+ m): Medium- to coarse-grained sandstone as in previous unit, but with less conglomerate, and the largest conglomeratic clasts are mostly reworked lithified mudstone. Varies between friable and ledge-forming beds. Horizontal and dipping planar beds, and trough cross beds. The unit fines upwards somewhat, with fine- to medium-grained sandstone predominating in the upper few meters of the unit. Top of unit incomplete, upper surface stripped by erosion

.

1. “Lot’s Wife section 3”/”PFNP-5” (total thickness: 32.50 m)

The cliffs along the north side of Agate Mesa give excellent exposures though the lower part of the Sonsela Member, including the contact with the top of the Blue Mesa Member. Roadifer [17] and Herrick [52] measured sections at the same spot, at about 12S E0609915 N3862732 NAD 27 (Figures S9a-b). Herrick’s section is more detailed and it was possible to identify her units in outcrop. Her eleven units have been given a numbering system with unit 1 (0-2.66 m) representing the uppermost Blue Mesa Member, units 2a-b (2.46-8.44 m) being the Camp Butte beds, units 3a-e (8.44-17.62 m) being the lower Lot’s Wife beds, unit 3f and unit 4 (17.64-27.32) being the upper Lot’s Wife beds, and unit 5 (27.32-32.50 m+) being the Jasper Forest bed. Her section description was used for the measured section with two additions: there are several thick lenses of dark purple mudstone about 1 m thick interbedded in the Camp Butte beds, and the lower 3 meters of the Jasper Forest bed is composed of conglomerate with pebble-cobble sized clasts composed mostly of chert.

1. Teepees to Camp’s Butte Section (composite) (total thickness: 9.3 m)

The Newspaper Rock Bed, which lies in the middle of the Blue Mesa Member, experiences numerous facies changes in the Tepees area of the park (Demko [47]), and to the east of the main park road it thins to a distinctive reddish band a few meters thick which can be traced throughout the Tepees area, almost to the base of Camp’s Butte. The lower part of a composite section was measured at 12S E0612452 N3867253 NAD 27 (the foreground in Figure S9b). A distinctive yellowish layer (unit 4a) exposed just a few meters above the Newspaper Rock Bed can be physically traced to the base of Camp’s Butte, and the upper part of the composite section was measured here from 12S E0612547 N3867188 NAD 27 (Figure 1e, Figure S9b). This is roughly the same area where Lucas [3] measured units 12-15 of his type section for the Blue Mesa Member, and where Therrien and Fastovsky [108] measured sections 1-3 for their “Dinosaur Wash” study area.

Blue Mesa Member

1. 0-2.3 m (thickness: 2.3 m): Well-lithified silty claystone, mottled light greenish gray (5GY 8/1), greenish gray (5GY 6/1), grayish blue (5PB 5/2), and grayish red purple (5RP 5/2). Upper contact gradational.
2. 2.3-4.7 m (thickness: 4.7 m): Newspaper Rock Bed. Moderate brown (5YR ¾) silty claystone with patches of light olive brown (5Y 5/6). Upper contact gradational.
3. 4.7-10.1 m (thickness: 5.4 m): Predominantly siltstone with extremely variable coloration; the lower part of the unit is mottled grayish red purple (5RP 4/2) with light greenish gray mottling, while the top meter is light olive gray (5Y 6/1) and medium bluish gray (5B 5/1). There is a discontinuous lens of light gray very fine-grained sandstone, about 20 cm thick, near the base of the unit, and the unit fines up back into siltstone above it. Upper contact sharp.
4. (4a) 10.1-11.3 m (thickness: 1.2 m): The base of this unit is about 20 cm of yellowish gray (5Y 7/2) claystone, overlain by a meter of muddy medium- to coarse-grained sandstone which is intensely mottled dark yellowish orange (10YR 6/6), moderate yellow (5Y 7/6), and moderate reddish brown (10R 4/6). This bed also contains gypsum and carbonized plant material. Laterally, this unit grades into a light bluish, extremely muddy, coarse- to very coarse-grained sandstone.

(4b) 11.3-17.5 m (thickness: 6.2 m): Predominantly calcareous light bluish gray (5B 7/1) siltstone, in places mottled grayish red purple (5RP 4/2), although the base of the unit is muddy very fine-grained sandstone, and there are several discontinuous lenses of light gray muddy, friable, fine- to medium-grained sandstone which vary greatly in thickness (usually no more than about 40 cm) and are often interbedded with siltstone. These are mostly concentrated near the top of the unit, and at this level there is also a very distinctive thin (usually no more than 1 cm thick) pinkish layer of gypsum, which weathers out as a striking bright pinkish carpet on the slopes. Dark-colored in-situ logs occur in the lower part of this unit, with the roots extending into unit 4a. Upper contact gradational.

1. 17.5-25.3 m (thickness: 7.8 m): Calcareous siltstone, distinctly darker than the previous unit, colors vary from grayish blue (5PB 5/2) to grayish red purple (5RP 5/2) to grayish purple (5P 4/2). The top meter or so below the Camp Butte beds is yellowish gray (5Y 8/1). As in the previous unit, there are several discontinuous lenses of light gray muddy, friable, fine- to medium-grained sandstone which vary greatly in thickness (usually no more than about 40-50 cm), and often interbedded with siltstone. Upper contact sharp.

Camp Butte beds (Sonsela Member)

1. 25.3-34.9 m (thickness: 9.6 m): Conglomeratic sandstone capping Camp’s Butte. Predominantly medium- to coarse-grained sandstone, with extensive trough cross-beds, dipping planar cross-beds, and horizontal planar beds. There are abundant granule- to pebble-sized clasts scattered throughout, usually no more than 2 cm in diameter and composed of chert and re-worked lithified mudstone, although they are also concentrated in discontinuous conglomeratic lenses generally 10-40 cm thick. In the upper part of the unit, there is a layer of massive (cobble sized, up to 15 cm across) chunks of re-worked Blue Mesa Member mudstone overlying a lens of muddy very fine-grained sand and silt. The top meter of the unit capping Camp’s Butte is relatively muddy and dark reddish brown, and is at about the gradational contact with the Lot’s Wife beds. Top of unit stripped by erosion.
2. Blue Mesa Pronghorn Trail Section (total thickness: 50.75 m)

The northern face of Blue Mesa (Figures S9e-f), as with that of Agate Mesa, provides excellent exposures of the entire lower part of the Sonsela Member, as well as exposing the contact with the top of the Blue Mesa Member in the badlands below. The Blue Mesa Pronghorn Trail section was measured a short distance to the east of the Blue Mesa loop road, in the same area as Woody’s [42] Blue Mesa 1 section. Due to difficulty in precisely identifying Woody’s units in outcrop, it was decided to re-measure the section, the base of which is at 12S E0614297 N3866933 NAD 27.

Blue Mesa Member

1. (1a) 0-2.8 m (thickness: 2.8 m): Grayish-red-purple (5RP 4/2) silty claystone with fine yellowish-gray (5Y 8/1) mottling (mottles are mostly a few millimeters in diameter) and root traces. Some carbonate nodules (4-5 cm in diameter) are also present. Upper contact gradational.

(1b) 2.8-3.65 m (thickness: 0.85 m): Medium bluish gray (5B 5/1) silty claystone with fine (millimeter scale) light olive gray (5Y 6/1) mottling. Upper contact gradational.

(1c) 3.65-4.05 m (thickness: 0.4 m): Yellowish-gray (5Y 8/1) silty claystone. Upper contact sharp.

Camp Butte beds

1. (2a) 4.05-4.45 m (thickness: 0.4 m): Mottled dark yellowish-orange (10YR 6/6) and very pale orange (10YR 8/1), extremely muddy medium- to coarse-grained sand, locally pebble conglomerate with clasts generally no larger than 1 cm. Sand and gravel clasts composed predominantly of chert and quartzite. Upper contact sharp.

(2b) 4.45-5.85 m (thickness: 1.4 m): Muddy and extremely siliceous medium- to coarse-grained sandstone fining upwards into medium-grained sandstone, some scattered pebble-sized (less than 1 cm) rip-up clasts of very pale orange (10YR 8/1) mudstone, giving the whole unit a yellowish color in weathered outcrop. Gently dipping planar cross-bedding and trough cross-bedding. Dark orange petrified wood occurs in this unit. Upper contact sharp.

(2c) 5.85-10.45 m (thickness: 4.6 m): Very complexly interbedded unit. Predominantly medium- to coarse-grained sandstone, the bulk of which is somewhat muddy and tends to be pinkish in weathered outcrop. However, densely interbedded in the pinkish sandstone are more resistant clast-supported lenses which are bluish-gray and generally 10-30 cm thick, most of which are also medium- to coarse-grained (but some are very fine- and fine-grained), which weather into “hoodoos.” The base of the unit is locally conglomeratic, with pebble-sized clasts generally no more than 1 cm in diameter composed of chert, quartzite, and reworked sedimentary clasts. This unit is mostly horizontal and gently dipping planar cross-bedding and trough cross-bedding, although some of the resistant lenses are ripple cross-laminated. Upper contact gradational, upper part of unit locally grades laterally into unit 3a.

Lot’s Wife beds

1. (3a) 10.45-15.25 m (thickness: 4.8 m): Complexly interbedded and laterally variable unit, lower of two fining upward sequences in the lower Lot’s Wife beds. Grayish-pink (5R 8/2) very fine- to fine-grained sand interbedded with muddier pale red (10R 6/2) beds, as well as pinkish medium- to coarse-grained sandstone as in the previous unit. The unit laterally grades into a fining upward sequence in which the base of the unit is as just described, but fines upwards into grayish-red-purple (5RP 4/2) and dark reddish-brown (10R 3/4) silt to very fine-grained sand with yellowish-gray (5Y 8/1) mottling. Upper contact sharp.

(3b) 15.25-23.05 m (thickness: 7.8 m): Second fining upward sequence in the lower Lot’s Wife beds. Yellowish-gray (5Y 8/1) very fine-grained sand interbedded with lenses (up to about 1 m thick) of pinkish-weathering medium- to coarse-grained sand, grading up into silt to very fine-grained sand varying in color from grayish-blue (5PB 5/2) to dark reddish-brown (10R 3/4). Upper contact sharp.

1. (4a) 23.05-26.15 m (thickness: 3.1 m): Upper Lot’s Wife beds. Very light gray (N8) medium-grained muddy sand, grading up into sandy siltstone of the same color with lenses of grayish-blue (5PB 5/2) silt to very fine-grained sand. Upper contact sharp.

(4b) 26.15-37.35 m (thickness: 11.2 m): Upper Lot’s Wife beds. The base of the unit is an extremely muddy reddish medium- to coarse-grained siliceous sand layer about 40 cm thick, locally conglomeratic with pebble sized clasts exceeding 1 cm in diameter, which fines up into very fine- to fine-grained sand, mostly yellowish gray (5Y 8/1) and dominated by faint horizontal planar-bedding; the top 40-50 cm is light brown (5YR 6/4). Upper contact sharp.

Jasper Forest bed

1. (5a) 37.35-40.95 m (thickness: 3.6 m): Extremely muddy and poorly sorted conglomerate; sand-sized clasts mostly medium- to very coarse-grained, gravel clasts are mostly subangular to subrounded, and mostly pebble-sized (a few centimeters in diameter) although cobbles reaching or exceeding 10 cm are common. These clasts are mostly composed of chert, although quartzite and reworked sedimentary clasts composed of sandstone and mudstone were also noted. Dominated by horizontal and gently dipping planar bedding, and trough cross-bedding. Upper contact gradational.

(5b) 40.95-45.25 m (thickness: 4.3 m): Compositionally similar to previous unit except that dominated by sandstone; gravel-sized clasts, mostly pebble-sized, are scattered throughout and also concentrated in a few layers delineating horizontal planar beds and planar and trough cross-beds. Upper contact sharp.

(5c) 45.25-50.75 m (thickness: 5.5 m): Medium- to coarse-grained sandstone with bedding as in previous two units, pebble-sized clasts, mostly no more than about 1 cm in diameter (although a few reach cobble-size) , are present in the lower 50 cm of the unit but sparser higher up. Upper surface truncated by erosion.

1. North of Long Logs section (total thickness: 23.45 m)

This section (Figures S10a-b) at 12S E0605581 N3852976 NAD 27 illustrates the problems that can locally occur in placing the boundary between the Jim Camp Wash beds and Martha’s Butte beds. The “Little Battleship sandstone,” forming the cap on the small butte called the Little Battleship, is a good example of one of the blocky and yellowish Flattops One sandstones that are so distinct from any in the Jim Camp Wash beds. However, there is a very distinctive package of friable yellowish-gray sand pinching out immediately north the North of Long Logs section (equivalent to unit 8), which can be traced with difficulty to the base of the Martha’s Butte beds at the No Name Point 2b section. This yellow-gray sand is therefore considered to represent the base of the Martha’s Butte beds, and forms units 2a-2b in the Near Little Battleship section. However, it grades laterally into purple and grayish sandstones and mudstones at the Stemwedel locality section (units 2-3) that are not clearly distinguishable from those in the Jim Camp Wash beds.

Jim Camp Wash beds

1. 0-1.75 m (thickness: 1.75+ m): Siltstone with a little medium-grained sand mixed in, grayish red purple (5RP 4/2) with fine light gray mottling, slope-forming unit. Intermittently exposed about 40 cm above the (measured) base of the unit is a pinkish, well-cemented, medium- to coarse-grained quartz arenite usually about 5-10 cm thick, although it can be thicker. This is an unusual lateral facies change of the persistent reddish silcrete, which has its more usual character further south towards Long Logs. Base of the unit not exposed. Upper contact sharp.
2. 1.75-4 m (thickness: 2.25 m): Very fine- to fine-grained muddy, friable, slope-forming sandstone showing distinct candy-striping of pale red (10R 6/2) and pinkish gray (5YR 8/1), in which the pale bed bands tend to be muddier. Upper contact sharp.
3. 4-6.8 m (thickness: 2.8 m) Calcareous siltstone with some fine-grained sand mixed in, pale purple (5P 6/2) mottled with yellowish gray (5Y 8/1). The base of the unit is well-cemented, and the rest is full of pedogenic carbonate nodules. Upper contact sharp.
4. (4a) 6.8-7.5 m (thickness: 0.7 m): Long Logs sandstone. Medium- to coarse-grained, friable light gray sandstone interbedded with resistant, ledge-forming granule conglomerate (clasts mostly reworked pedogenic carbonate) with faint horizontal planar bedding.

(4b) 7.5-9.0 m (thickness: 1.5 m): Long Logs sandstone. Medium- to coarse-grained sandstone with faint planar and trough cross-bedding, faintly candy-striped with pale red (10R 6/2) and pinkish gray (5YR 8/1). Upper contact gradational with overlying fining upward sequence, somewhat arbitrary.

1. 9.0-13.6 m (thickness: 4.6 m): Fining upward sequence. Medium- to coarse-grained sand with faint horizontal planar and trough cross-bedding, fining up gradually to very fine-grained sand, and then in the upper two meters to grayish-red-purple (5RP 4/2) siltstone with greenish- gray (5GY 6/1) mottling. The silty part of the unit is calcareous, and full of densely packed pedogenic carbonate nodules. Upper contact sharp.
2. 13.6-15.9 m (thickness: 2.3 m): Fining upward sequence. Muddy fine- to medium-grained sandstone, with mottling and candy-striping of grayish red (10R 4/2) and yellowish gray (5Y 8/1), with the reddish bands tending to be muddier. This grades upwards into grayish-red-purple (5RP 4/2) very fine-grained sand to silt with greenish-gray (5GY 6/1) mottling. Upper contact is gradational.
3. 15.9-18.6 m (thickness: 2.7 m): Grayish-red-purple (5RP 4/2) siltstone with greenish-gray (5GY 6/1) mottling as in previous unit, but extremely calcareous and full of carbonate nodules and sparse root traces. Upper contact sharp.

Martha’s Butte beds

1. 18.6-21.1 m (thickness: 2.5 m): Well-cemented, pale purple (5P 6/2) siltstone with light greenish-gray (5GY 8/1) mottling. Laterally equivalent with units 2a-2b of Near Little Battleship section. Upper contact sharp.
2. (9a) 21.1-22.2 m (thickness: 1.1 m): Yellowish-gray (5Y 8/1) muddy, friable, fine- to medium-grained sand, locally with some thin (few cm thick) resistant lenses of ripple cross-laminated sand in the lower part of the unit.

(9b) 22.2-23.45 m (thickness: 1.25 m): “Little Battleship sandstone,” a lower Flattops One sandstone. Medium- to coarse-grained, resistant, ledge-forming, planar cross-bedded sandstone, with a few centimeters of pebble conglomerate (clasts of reworked intrabasinal pedogenic carbonate) at the base.

1. Near Little Battleship section (total thickness: 18.05 m)

The Little Battleship is a small mesa capped by the “Little Battleship sandstone,” which lies above the yellow-gray sand package forming the base of the Martha’s Butte beds. Several important vertebrate localities occur within this interval in this area, and have been tied to this section. A composite section (Figure S10e) was measured a short distance to the north of the Little Battleship which encompasses most of the Martha’s Butte beds, ending a short distance below the upper contact with the monotonous purple beds. The lower part of the section (Figure S10c) which measured up to the base of the Little Battleship sandstone, was measured from 12S E0606518 N3853673 NAD 27, and the upper part of the section (Figure 23d) was measured from 12S E0606462 N3853772 NAD 27.

Jim Camp Wash beds

1. 0-1 m (thickness: 1 m): Pale purple (5P 6/2) and light greenish-gray (5GY 8/1) mottled siltstone containing orange-sized carbonate nodules. Upper contact sharp.

Martha’s Butte beds

1. (2a) 1-4.7 m (thickness: 3.7 m): “Yellowish-gray sand package.” Generally fining upward sequence of mostly friable, fairly muddy, pale olive (10Y 6/2), slope-forming, texturally and compositionally immature sandstone containing abundant muscovite and biotite. The sandstone fines from medium- to coarse-grained sand to fine- to medium-grained sand, with a few horizons of modest-sized (few centimeters in diameter) carbonate nodules in the upper part of the unit. Locally, there is conglomerate at the base of the unit with granule- to pebble-sized clasts composed of re-worked pedogenic carbonate. Upper contact sharp.

(2b) 4.7-6.5 m (thickness: 1.8 m): Pale purple (5P 6/2) and greenish-gray (5GY 6/1) siltstone to very fine-grained sandstone, shot through with tiny (millimeter-sized) pockets of gypsum. The base of the unit is a horizon of pedogenic carbonate nodules, no larger than a few centimeters in diameter. Upper contact sharp.

1. 6.5-7.75 m (thickness: 1.25 m): “Little Battleship sandstone,” a lower Flattops One sandstone. Highly siliceous coarse- to very coarse-grained sandstone dominated by gently dipping planar bedding, possibly representing lateral accretion bedding. Discontinuous lenses of conglomerate 30-40 cm thick with granule- to pebble-sized clasts up to a centimeter or more in diameter, mostly composed of chert and quartzite. This unit is unusually siliceous for the Martha’s Butte beds. Resistant ledge-forming unit, upper contact gradational.
2. (4a) 7.75-10.05 m (thickness: 2.3 m): Friable, slope-forming, pale olive (10Y 6/2) fine-grained sandstone, patches of which are dark yellowish-orange (10YR 6/6) and contain gypsum and white and orange petrified wood. Interbedded are resistant, dusky yellow (5Y 6/4), gently dipping lenses (at about 15-20°) of medium-grained, planar-bedded sandstone 10-20 cm thick.

(4b) 10.05-18.05 m (thickness: 8 m): Light gray (N7) siltstone to very-fine grained sandstone, interbedded with yellowish-gray (5Y 7/2) fine-grained sand, all gently dipping 15-20°. The upper few meters are interbedded with grayish-red (5R 4/2) siltstone. Individual layers of sandstone and siltstone are usually only about 10-30 cm thick.

1. Stemwedel Site section (total thickness: 48.50 m)

The Stemwedel vertebrate locality is in the East Flattops area, near the base of a mesa which is truncated just below the Flattops 3 Bed (Figures S10f-g). The section for the site was measured up the side of the mesa, the base of which is at 12S E0607365, N3853104 NAD 27. Physical walking out of the base of unit 2 shows that it correlates with the “yellow-gray sand package” considered to mark the base of the Martha’s Butte beds (see discussion under North of Long Logs section).

Jim Camp Wash beds

1. 0-1.1 m (thickness: 1.1 m): Mottled greenish-gray (5GY 6/1) and grayish-red-purple (5RP 4/2) siltstone. Upper contact sharp.

Martha’s Butte beds

1. 1.1-4.9 m (thickness: 3.8 m): Greenish-gray (5GY 6/1) fine- to medium-grained sandstone, mostly muddy and friable although interbedded with resistant fine- to medium-grained sand lenses 5-10 cm thick, and with grayish-purple (5P 4/2) siltstone. Cut and fill deposition; the sand and silt layers are laterally discontinuous, variable in thickness, and show variable degrees of dip. The top of the unit is a zone of mottled grayish red purple (5RP 4/2) and mottled greenish gray (5GY 6/1). Upper contact sharp.
2. 4.9-10.2 (thickness 5.3 m): Fining upward sequence. The base of the unit in a lens of fine- to medium-grained, resistant, yellowish-gray (5Y 8/1) sandstone with horizontal planar bedding, planar cross-bedding, and ripple cross-lamination. At its thickest point, this lens is about 1.3 m thick but it pinches out laterally. Grades vertically into friable, slope-forming greenish-gray (5GY 6/1) fine- to medium-grained sandstone, and higher up into pale purple (5P 6/2) siltstone, the upper part of which is heavily pedogenically altered with intense yellowish-gray (5Y 8/1) mottles and orange-sized carbonate nodules. Although only a couple meters thick where the overlying sandstone lens (unit 4a) is thickest, this unit thickens to about 4 meters laterally where this overlying lens thins. Upper contact sharp.
3. (4a) 10.2-12.6 m (thickness: 2.4 m): A lens of resistant fine- to medium-grained sandstone dominated by horizontal planar bedding, interbedded with muddy and friable sandstone of the same grain size, and grayish–red-purple (5RP 4/2) and light greenish-gray (5GY 8/1) siltstone. There are patches of pebble conglomerate with clasts of reworked intrabasinal pedogenic carbonate at the base. At its thickest point, this lens is about 2.4 meters thick, but it pinches out laterally.

(4b) 12.6-19.95 m (thickness 7.35 m): Fining upward sequence. Friable, yellowish-gray (5Y 8/1), very fine- to fine-grained sand, grading into grayish-red (5R 4/2) very fine- grained sand to silt, and then into well-cemented silt of mottled greenish-gray (5GY 6/1) and grayish-red-purple (5RP 4/2), full of pedogenic carbonate nodules as with the top of the previous unit. Upper contact sharp.

(4c) 19.95-22.35 m (thickness: 2.4 m): Pale reddish-brown (10R 5/4) very fine-grained sand. Upper contact sharp.

1. 22.35-27.95 m (thickness: 5.6 m): Fining-upward sequence. The base of the unit is fairly resistant, slightly muddy, fine- to medium-grained yellowish-gray (5Y 8/1) sandstone, which grades upwards into pale red-purple (5RP 6/2) silt to very fine-grained sand containing carbonate nodules and light greenish-gray (5GY 8/1) mottling. Upper contact sharp.

Petrified Forest Member

1. 27.95-34.70 m (thickness: 6.75 m): Monotonous purple beds. Grayish-red-purple (5RP 4/2) siltstone with fine light greenish-gray (5GY 8/1) mottling, contains sparse lenses of extremely muddy medium-grained sand up to about 1 meter thick. Upper contact sharp.
2. (7a) 34.70-34.80 m (thickness: 0.1 m): Remnant of Flattops 2 Bed. Discontinuous, resistant thin (5-10 cm thick) lenses of granule conglomerate with horizontal planar bedding, interbedded with yellowish-gray (5Y 8/1) fine- to medium-grained sandstone.

(7b) 34.80-48.50 m (thickness: 13.7 m): Mostly fining upward sequence. The base of the unit is yellowish-gray (5Y 8/1) fine- to medium-grained sandstone interbedded with grayish-red (5R 4/2) muddy sandstone of similar grain size. This package fines upwards into pale reddish-brown (10R 5/4) silt to very fine-grained sand, then into light brownish-gray (5YR 6/1) extremely muddy medium-grained sand. The top of the unit is stripped by erosion, and covered with remnants of the Flattops 3 Bed, which was probably no more than a few meters above the top of the hill.

1. Near Milkshake Quarry section (total thickness: 15 m)

The Milkshake Quarry section is the most southeasterly within PEFO. It lies a short distance above the Rainbow Forest Bed, and ends not far below a very thick ledge-forming sandstone which is probably equivalent to the Long Logs sandstone. The section (Figures S10h-i) was measured at 12S E0605069 N3850861 NAD 27. A thin silcrete horizon capping the section is probably not the persistent red silcrete, as it far too high above the Rainbow Forest Bed. However, the mottled siltstone of unit 2b resembles that of the persistent red silcrete zone (even where the silcrete itself is absent) in the area north of Long Logs, and may represent that horizon.

Jim Camp Wash beds

1. (1a) 0-1.5 m (thickness: 1.5+ m): Grayish-red (10R 4/2) and yellowish-gray (5Y 8/1) mottled siltstone with lenses of pinkish-gray (5YR 8/1) fine-grained sandstone. The upper part of the unit is silt to very fine-grained sand, which is mottled pale red (10R 6/2) and yellowish gray (5Y 8/1). The Rainbow Forest Bed is exposed a short distance to the southwest, and the upper contact is of that unit is probably not far below the surface here. Upper contact gradational.

(1b) 1.5-3.2 m (thickness: 1.7 m): Grayish-red-purple (5RP 4/2) siltstone with yellowish-gray (5Y 8/1) mottling, contains a lens of pinkish-gray fine- to medium-grained sandstone with a maximum thickness of about 50 cm. Upper contact sharp.

1. (2a) 3.2-4.6 m (thickness: 1.4 m): Fining upward sequence. Slightly resistant medium- to coarse-grained siliceous sandstone with some biotite and muscovite, containing some granule-sized clasts of chert and quartzite in the lower 15 cm. Upper contact gradational.

(2b) 4.6-6.5 m (thickness: 1.9 m): Persistent reddish silcrete zone? Fining upward sequence. Very fine-grained yellowish-gray (5Y 8/1) sand grading upwards into siltstone of mottled grayish red purple (5RP 4/2) and yellowish gray (5Y 8/1). Upper contact sharp.

1. (3a) 6.5-7.9 m (thickness: 1.4 m): Moderate brown (5YR 4/4) siltstone to very fine-grained sandstone with fine light greenish-gray (5GY 8/1) mottling.

(3b) 7.9-9.8 m (thickness: 1.9 m): Grayish-red-purple (5RP 4/2) siltstone with fine light greenish-gray (5GY 8/1) mottling. Upper contact sharp.

1. (4a) 9.8-12.2 m (thickness: 2.4 m): Slightly resistant fine- to medium-grained clast-supported sandstone grading up into pale reddish-brown (10R 5/4) very fine-grained sandstone with light greenish-gray (5GY 8/1) mottling. Upper contact gradational.

(4b) 12.2-14.6 m (thickness: 2.4 m): Pale purple (5Y 6/2) siltstone with light greenish-gray (5GY 8/1) mottling.

1. 14.6-15 m (thickness: 0.4 m): Light greenish-gray (5GY 8/1) very fine-grained sandstone. At the base of the unit is a silcrete layer about 4 cm thick, which is dark yellowish-orange (10YR 6/6) on the outside and blackish-red (5R 2/2) on the inside. The unit is incomplete, and the upper surface is stripped by erosion.
