# Supplementary figures and images for: Revised Lithostratigraphy of the Sonsela Member (Chinle Formation, Upper Triassic) in the Southern Part of Petrified Forest National Park, Arizona
Source: PLoS One. 2010 Feb 19;5(2):e9329. doi: 10.1371/journal.pone.0009329 (PMC2824835; doi:10.1371/journal.pone.0009329)

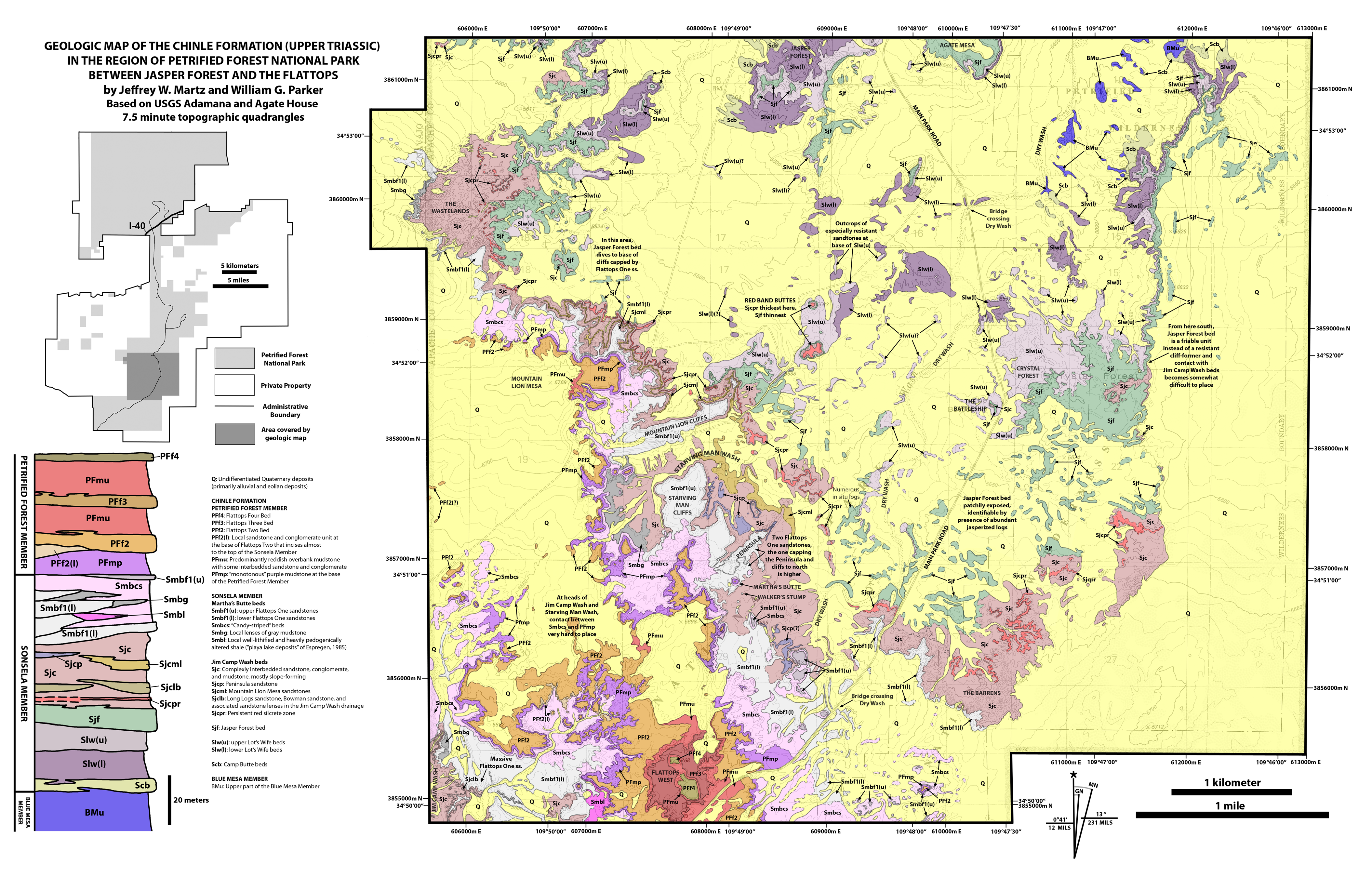

Supplement: Figure S1 — Geologic map of the Chinle Formation (Upper Triassic) in the region of Petrified Forest National Park between Jasper Forest and the Flattops. The location of the map is shown by the smaller park map on the upper left, and unit symbols are explained by the stratigraphic column and key on the lower left. Dashed contact lines indicate where a contact is either arbitrary due to being gradational, or poorly exposed. The contacts for Quaternary deposits, which are often thin layers of wind-blown sand patchily concealing Chinle Formation outcrops, are particularly arbitrary, and should be taken with a grain of salt. (9.47 MB TIF) [file pone.0009329.s002.tif]

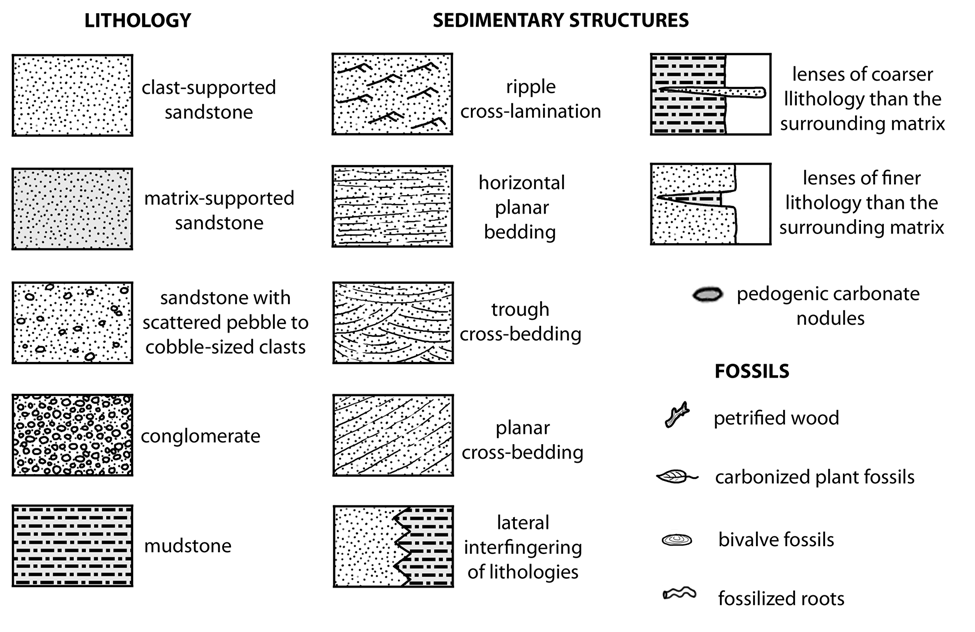

Supplement: Figure S2 — Key to symbols used in measured sections. (0.18 MB TIF) [file pone.0009329.s003.tif]

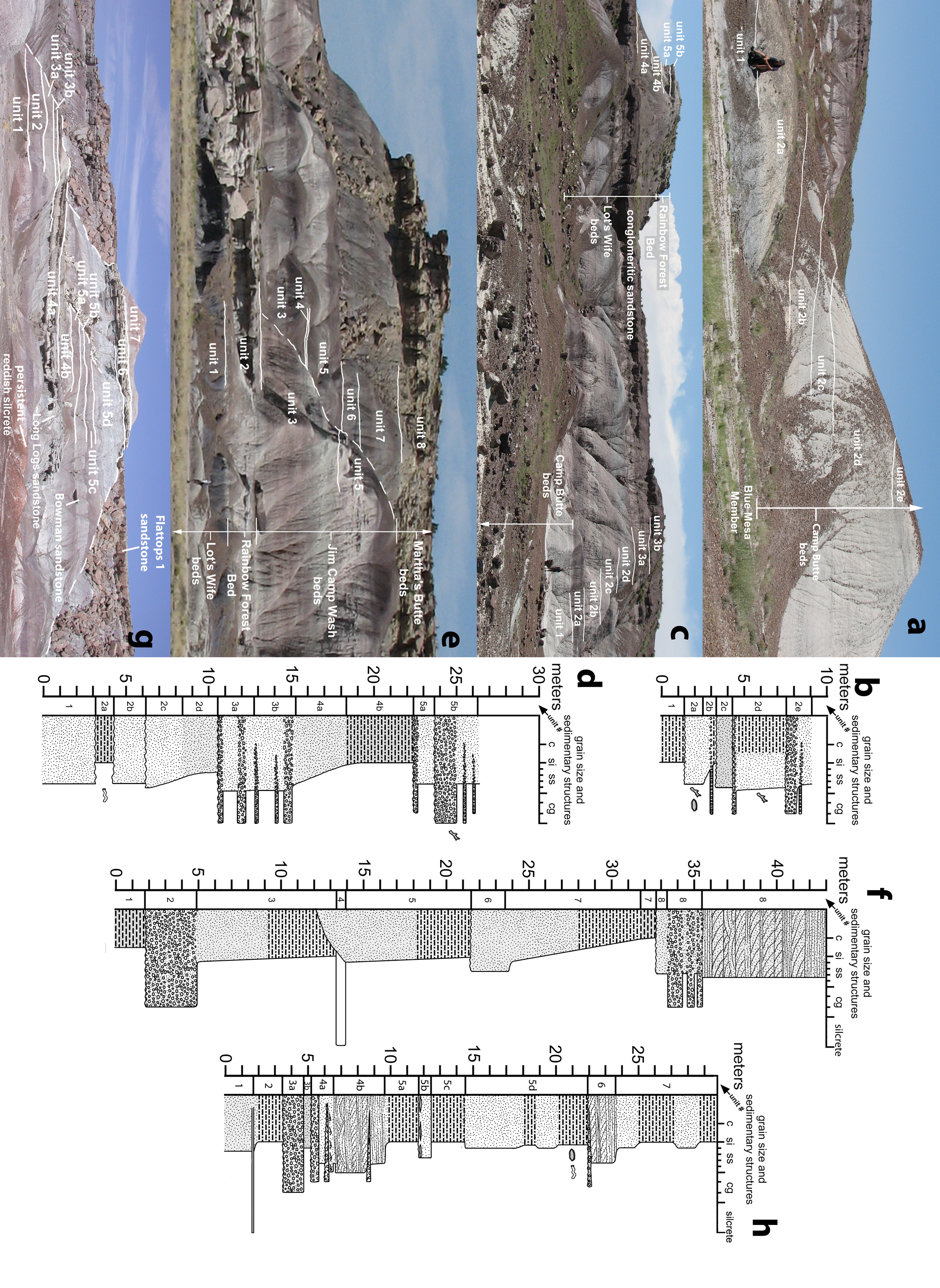

Supplement: Figure S3 — Labeled photographs and diagrams of measured sections 1–4. South End Knob at 12S E0602076 N3851723 NAD 27, photo (a) and section (b); South End Cliff at 12S E0601939 N3851827 NAD 27, photo (c) and section (d); “PFNP-14”/“Giant Logs section” of Roadifer [17] and Heckert and Lucas [11] at 12S E0602800 N3854095 NAD 27, photo (e) and section (e–f); East of Petroglyphs at 12S E0604707 N3854159 NAD 27 photo (g) and section (g–h). (7.55 MB TIF) [file pone.0009329.s004.tif]

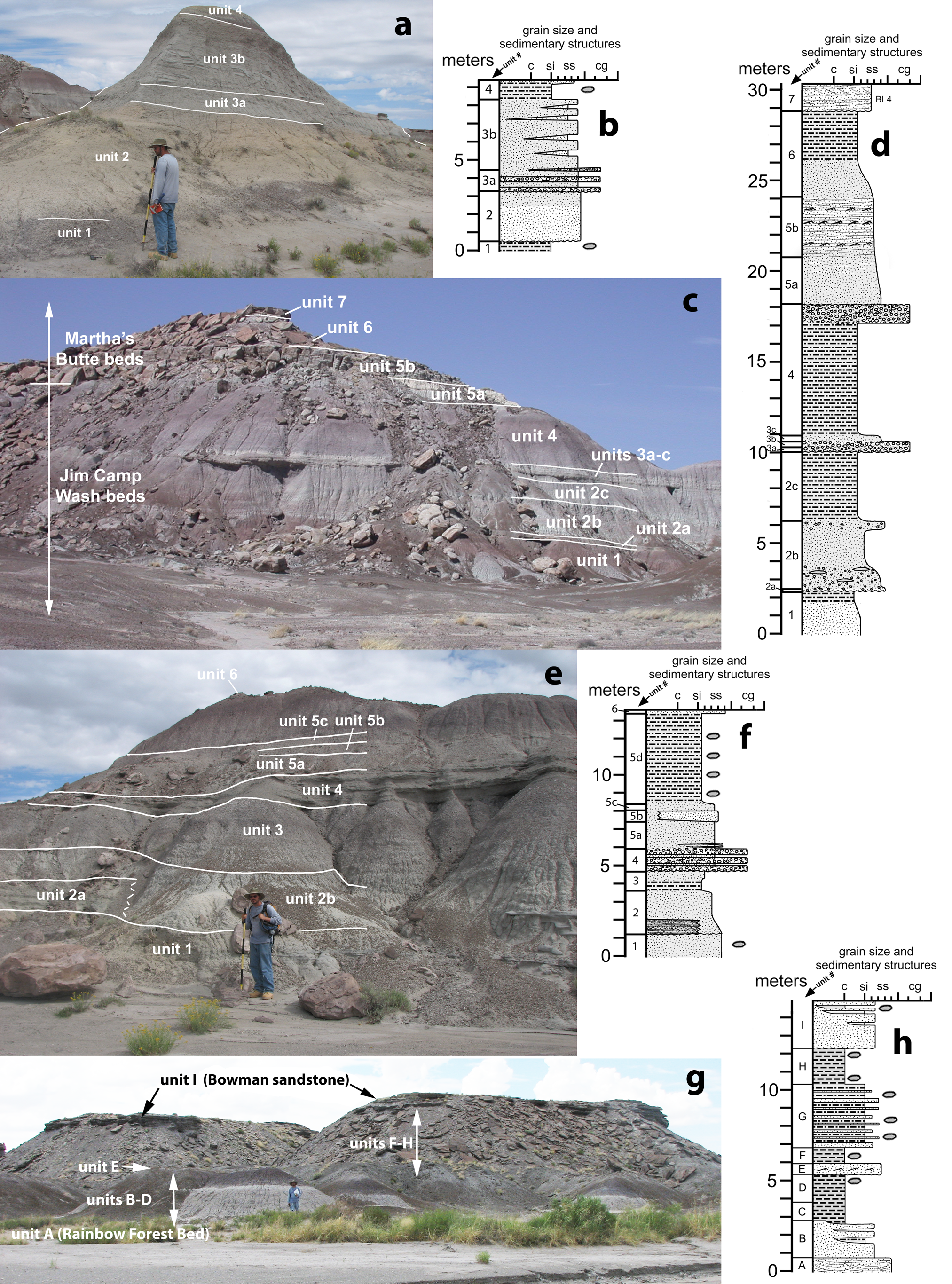

Supplement: Figure S4 — Labeled photographs and diagrams of measured sections 5–8. Bowman 2/Bowman South at 12S E0604866 N3854341 NAD 27, photo (a) and section (b); Bowman 3 at 12S E0604793 N3854410 NAD 27, photo (c) and section (d); Bowman 1 at 12S E0604831 N3854555 NAD 27, photo (e) and section (f); No Name Point 3 of Woody [42] at 12S E0603673 N3854544 NAD 27 photo (g) and section (h). (7.90 MB TIF) [file pone.0009329.s005.tif]

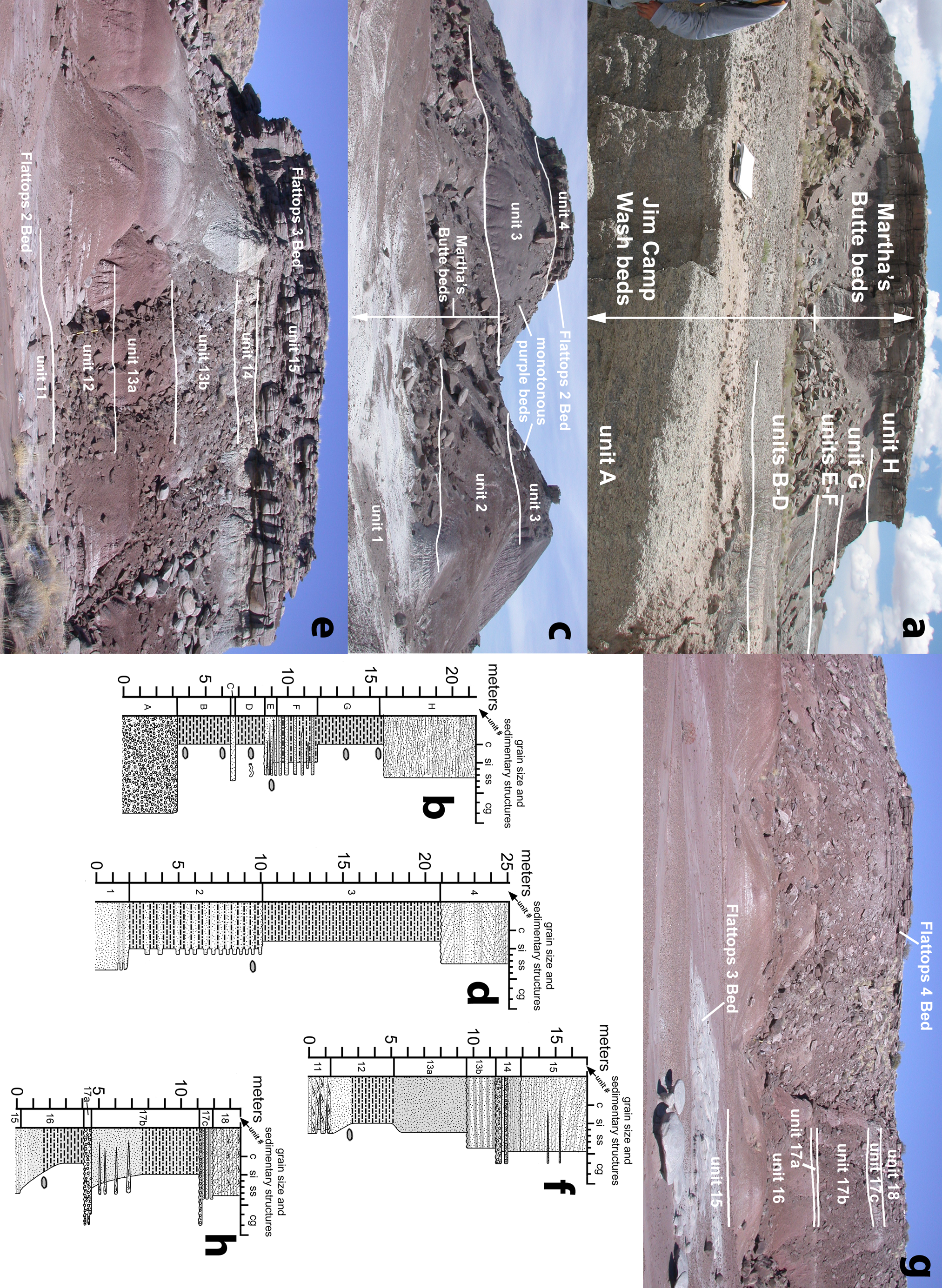

Supplement: Figure S5 — Labeled photographs and diagrams of measured sections 9–12. No Name Point 2b section of Woody [42] at 12S E0606203 N3854676 NAD 27 photo (a) and section (b); Dalton Site at 12S E0606877 N3855141 NAD 27, photo (c) and section (d); Lower “Flattops West” of Heckert and Lucas [11] photo at 12S E0607645 N3854991 NAD 27 (e) and section (f); Upper “Flattops West” section of Heckert and Lucas [11] at 12S E0607767 N3855109 NAD 27 photo (g) and section (h). (10.26 MB TIF) [file pone.0009329.s006.tif]

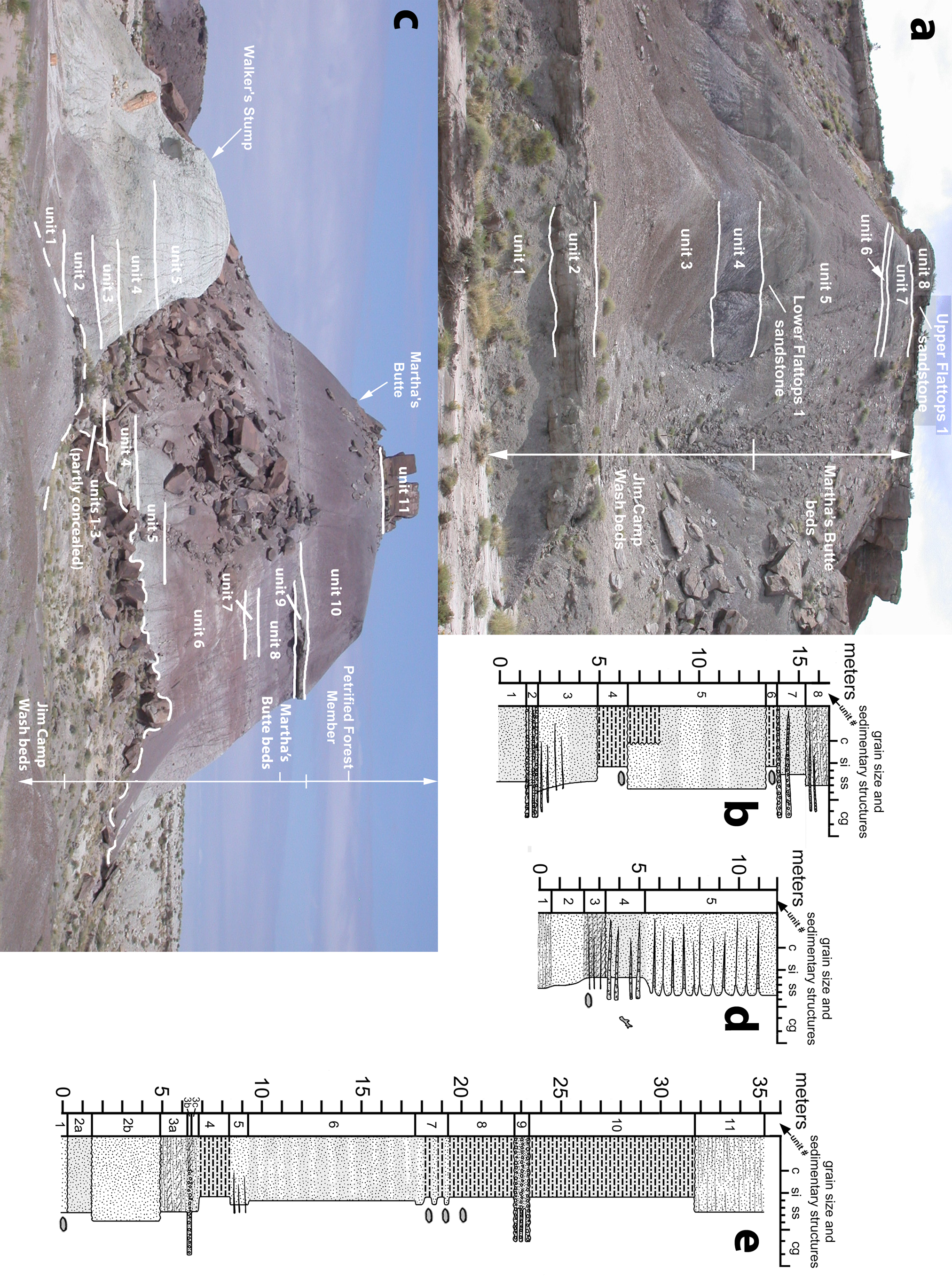

Supplement: Figure S6 — Labeled photographs and diagrams of measured sections 13–15. Dry Wash Bridge East at 12S E0608669 N3856310 NAD 27, photo (a) and section (b); Walker's Stump and Martha's Butte at 12S E0608292 N3856717 NAD 27, photograph (c); Walker's Stump section (d); Martha's Butte section (e). (7.75 MB TIF) [file pone.0009329.s007.tif]

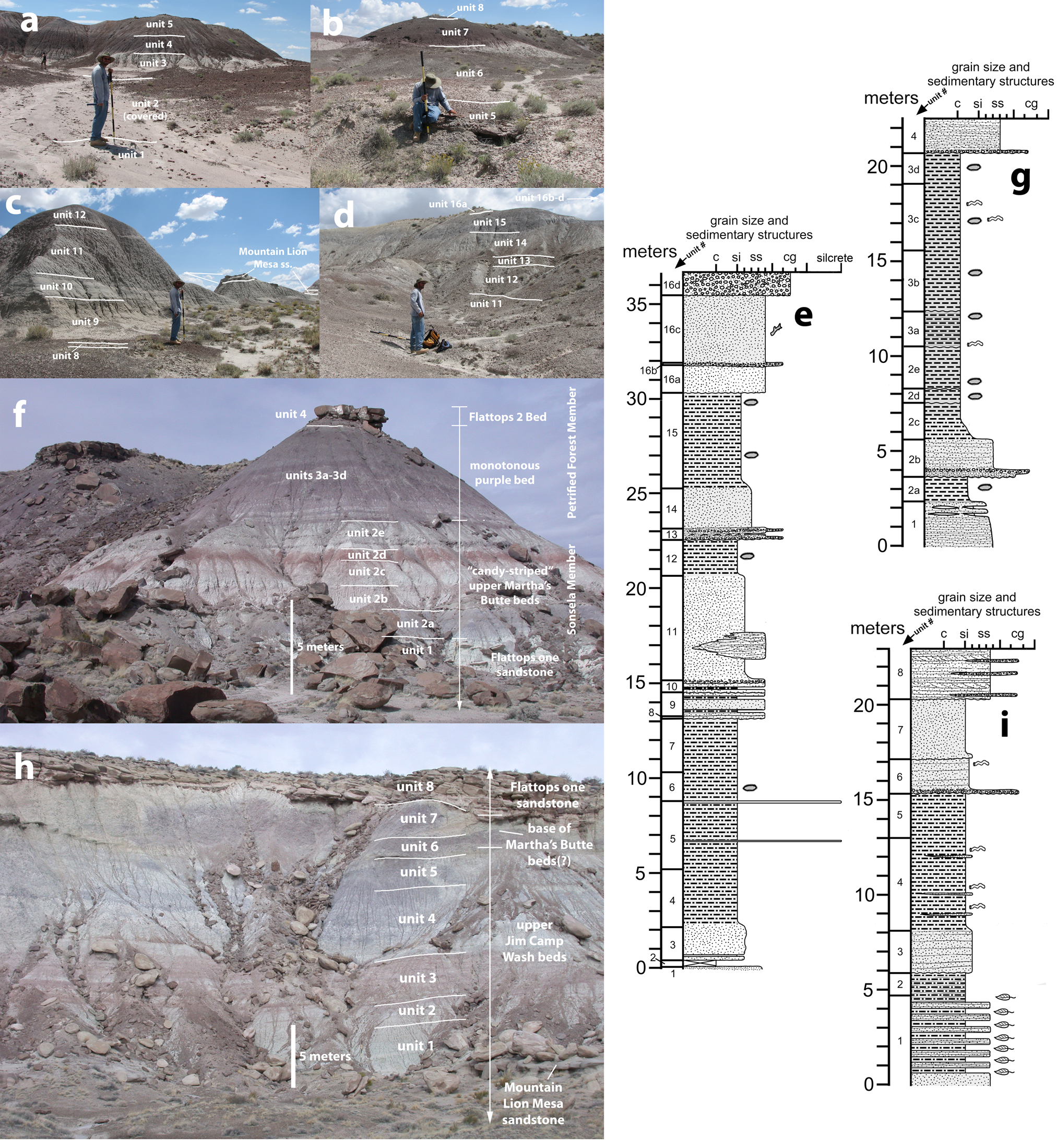

Supplement: Figure S7 — Labeled photographs and diagrams of measured sections 16–18. Photographs of micro-sections used to create composite section for the Peninsula at 12S E0608872 N3857800 NAD 27 (a), 12S E0608701 N3857648 NAD 27 (b), 12S E0608644 N3857522 NAD 27 (c), 12S E0608489 N3857317 NAD 27 (d), composite Peninsula section (e); “Gatesy's Plunge section 2” of Herrick [52] at 12S E0607320 N3858600 NAD 27, photo (f) and section (g), “Gatesy's Plunge section 4” of Herrick [52] at 12S E0607655 N3858302 NAD 27, photo (h) and section (i). (6.59 MB TIF) [file pone.0009329.s008.tif]

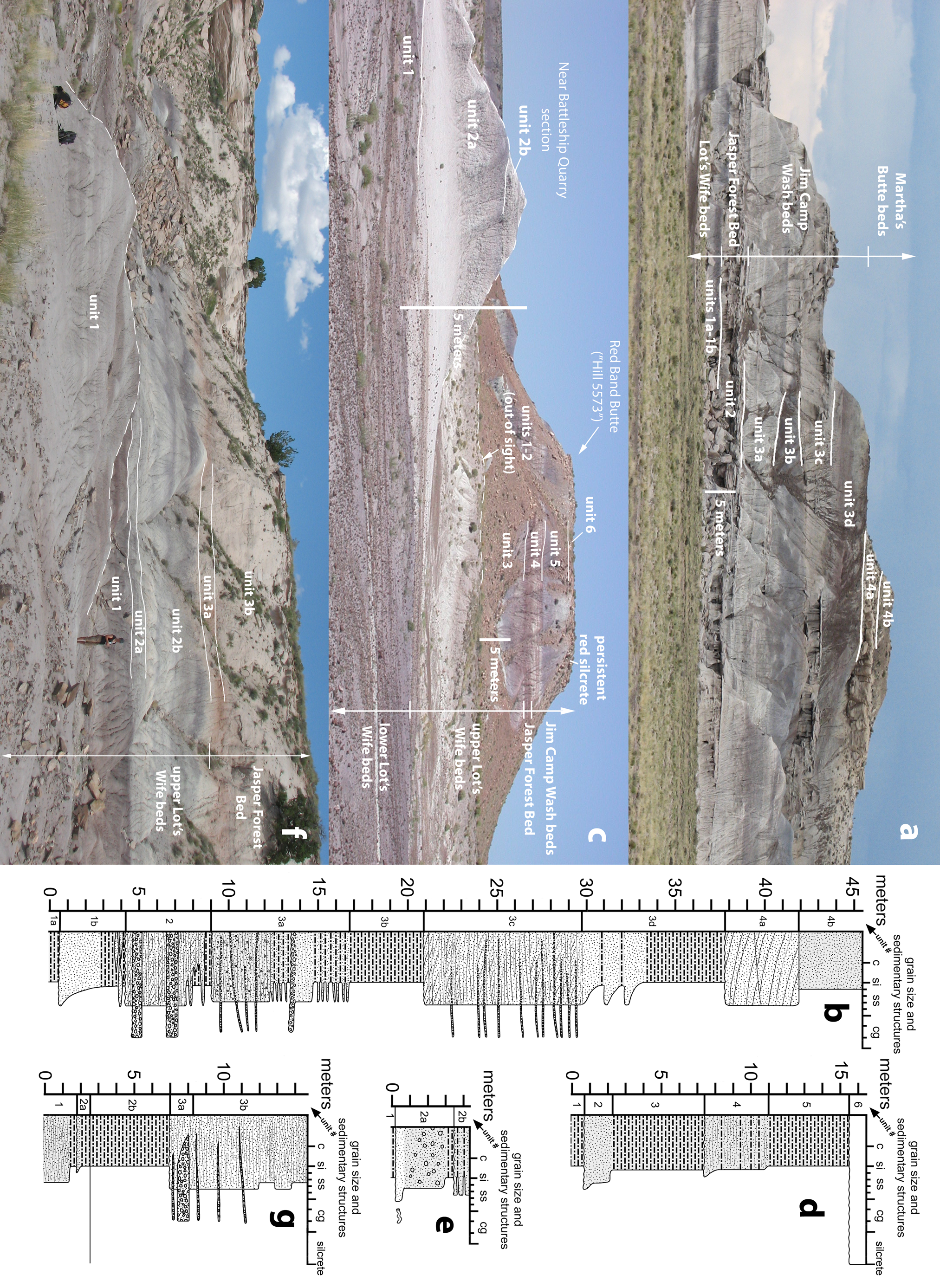

Supplement: Figure S8 — Labeled photographs and diagrams of measured sections 19–22. Mountain Lion Cliffs at 12S E0608065 N3858693 NAD 27, photo (a) and section (b), Photograph of more northerly Red Band Butte and Near Battleship Quarry photographed at about 12S E0609097 N3859146 NAD 27 (c), Red Band Butte section (d), Near Battleship Quarry section (e), Flag Canyon at 12S E0611631 N3859786 NAD 27, photo (f) and section (g). (9.01 MB TIF) [file pone.0009329.s009.tif]

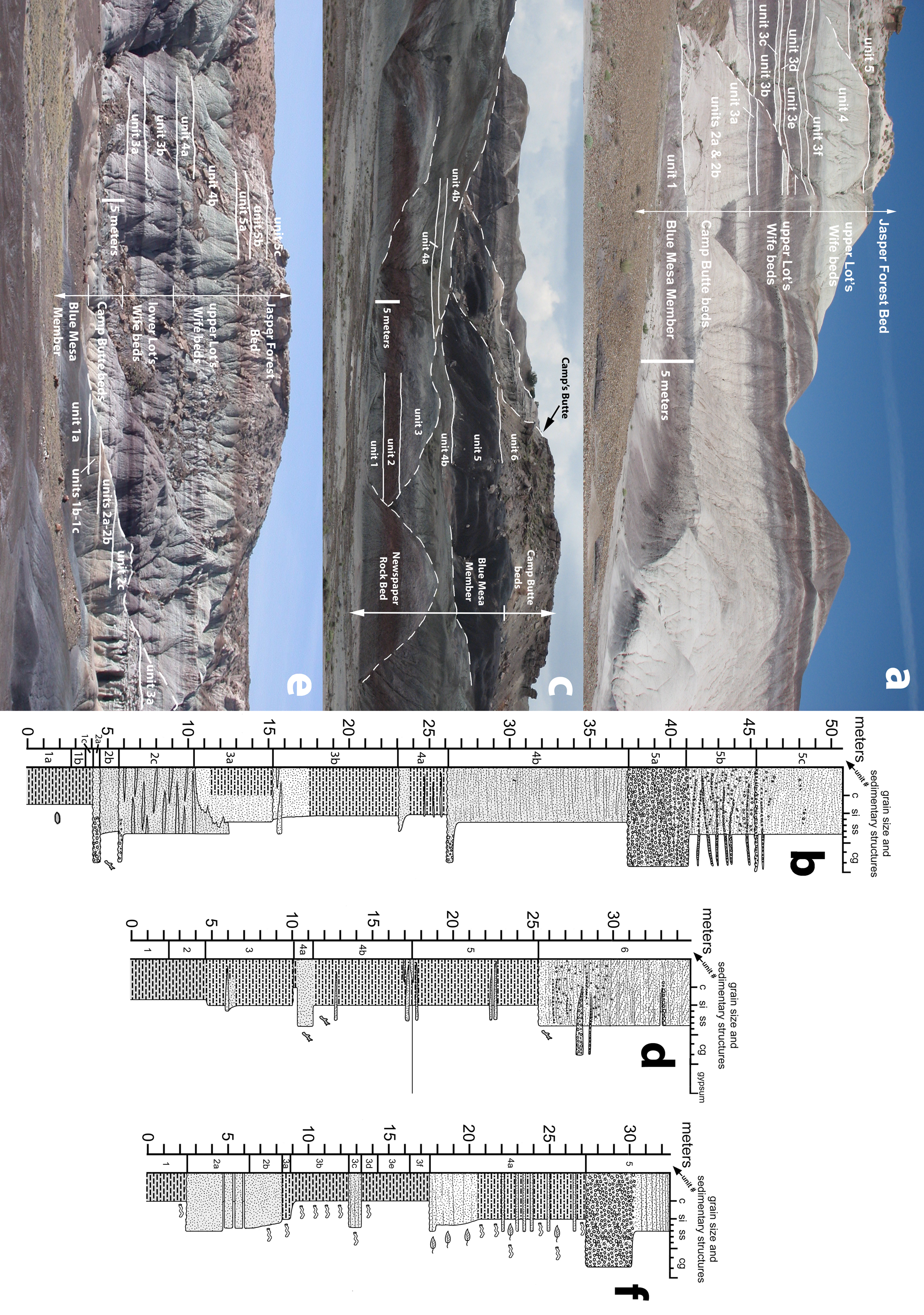

Supplement: Figure S9 — Labeled photographs and diagrams of measured sections 23–25. “Lot's Wife section 3”/“PFNP-5” sections of Herrick [52] and Roadifer [17] at 12S E0609915 N3862732 NAD 27, photo (a) and section (b); Tepees to Camp's Butte, photo with foreground at 12S E0612452 N3867253 NAD 27 (c) and section (d); Blue Mesa Pronghorn Trail at 12S E0614297 N3866933 NAD 27, photo (e) and section (f). (7.92 MB TIF) [file pone.0009329.s010.tif]

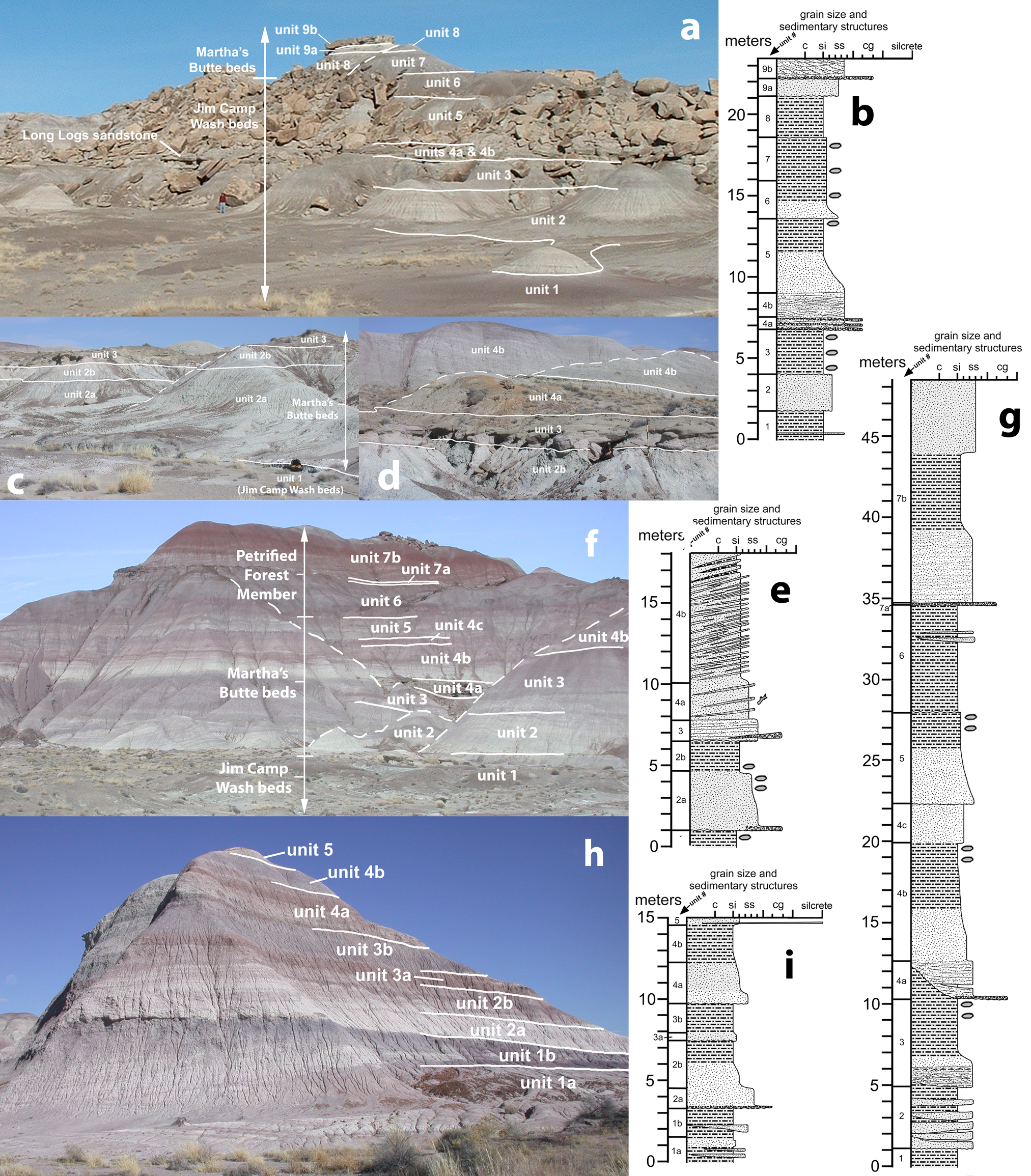

Supplement: Figure S10 — Labeled photographs and diagrams of measured sections 26–29. North of Long Logs at 12S E0605581 N3852976 NAD 27, photo (a) and section (b); Near Little Battleship, photo of lower part of section at 12S E0606518 N3853673 NAD 27 (c), photo of upper part of section at 12S E0606462 N3853772 NAD 27 (d), section (e); Stemwedel Site section at 12S E0607365 N3853104 NAD 27, photo (f) and section (g); Near Milkshake Quarry at 12S E0605069 N3850861 NAD 27, photo (h) and section (i). (6.81 MB TIF) [file pone.0009329.s011.tif]
